# Demand and supply side factors that drive delayed referrals from traditional birth attendants to public primary healthcare facilities: Insights from three states in Nigeria

**DOI:** 10.1371/journal.pgph.0003886

**Published:** 2024-12-02

**Authors:** Chinelo Ifeoma Okeke, Prince Agwu, Enyi Etiaba, Obinna Onwujekwe

**Affiliations:** 1 Department of Community Medicine, University of Nigeria Teaching Hospital (UNTH), Enugu, Nigeria; 2 Health Policy and Research Group (HPRG) University of Nigeria, Nsukka, Nigeria; 3 School of Humanities, Social Sciences, and Law, University of Dundee, Dundee, United Kingdom; 4 Department of Social Work, University of Nigeria, Nsukka, Nigeria; 5 Department of Health Administration and Management, College of Medicine, University of Nigeria, Enugu Campus, Enugu, Nigeria; University of Cape Town, SOUTH AFRICA

## Abstract

Despite global efforts encouraging institutional deliveries with skilled attendants, many pregnant women in developing countries such as Nigeria continue to rely on traditional birth attendants (TBAs) for child delivery. Attempts at weeding off TBAs have been firmly resisted by their clients who have developed confidence and trust in their services and herald them as first and trusted responders to child delivery cases. Unfortunately, recent evidence has shown that TBAs in servicing public trust and for other reasons, often do not timely refer their clients to the closest source of formal healthcare–primary healthcare centres (PHCs). Looking into the motivations for these reasons is the crux of the current study. Data were collected through qualitative interviews with 85 respondents across three states in Nigeria. The qualitative data comprised of 73 in-depth interviews and 12 focus group discussions**.** Respondents were TBAs and other informal health providers, formal health workers, policymakers, community leaders and service users. Data were analysed thematically to explore the demand and supply determinants of referrals from TBAs to the formal healthcare providers. Demand side factors that constrain referrals from TBAs to PHCs were community preference for TBAs, and cultural and religious inclinations of the consumers to TBAs’ practices of infusing religious teachings and rules into the process of child delivery. Supply-side factors were infrastructural deficits, staff shortages and lack of sanctions for poor practices in PHCs. Demand and supply-side factors constrain the referral of pregnant women from TBAs to the PHC facility for child delivery. These factors should be addressed, and innovative interventions used to link the TBAs to the formal health system for increased institutional deliveries with skilled birth attendants. The interventions could include incentives to TBAs to encourage early referrals.

## Background

Low- and middle-income countries (LMICs) continue to record alarmingly high rates of infant and maternal deaths, with almost 800 deaths occurring daily in 2020 from preventable causes related to childbirth [1]. Sub-Saharan Africa alone accounts for about 87% of global maternal deaths, with most of these women dying from complications during pregnancy and following childbirth [1]. In 2022, Sub-Saharan Africa had the highest neonatal mortality rate with 27 deaths per 1000 live births [2]. Approximately 75% of neonatal deaths occur within the first week of life and up to one million babies die within the first 24hours [2].

The World Health Organization (WHO) advocates for having a skilled birth attendant at delivery as a key intervention for reducing infant and maternal morbidity and mortality [3,4]. However, skilled birth attendants are typically available only in health facilities, leading to the propagation of institutional maternal care, especially during delivery in LMICs [4].

Globally not all women seek skilled care during delivery, with some opting for home births[5]. Several studies have reported factors responsible for this preference, including a mismatch between the needs of the women and the healthcare system, the desire for autonomy and control over the birthing process, limited economic and geographical access to health facilities, lack of transportation and delivery supplies, financial constraints, negative perceptions of healthcare staff, shortage of health workforce, poor attitude of healthcare workers, preference for traditional birth attendants (TBAs), traditional beliefs and lack of coordination of referrals from traditional birth attendants [4,6–9].

Evidence indicates that in LMICs such as Nigeria, TBAs, often with little or no formal training, provide a good proportion of maternal health services, especially to the poor and vulnerable women in local communities [10,11]. The strain on formal healthcare workers in Nigeria caused by staff shortages, long working hours and poor financial resources has led community members to increasingly rely on informal providers such as traditional birth attendants (TBAs) [12–15]. These TBAs are well known and trusted within their communities and attempts to phase them out have been met with strong resistance as the people have developed significant confidence in their services [16]. This realization has led to several interventions in the past aimed at improving maternal health indices, some of which include collaboration with, and training of TBAs despite ongoing concerns surrounding their usefulness and quality of services [10,17].

These training programs often involve partnerships with non-governmental organizations, government agencies, and community-based organizations in a bid to ensure acceptability by the TBAs and sometimes, incentives are provided as motivation to the TBAs to encourage prompt referrals [18]. The capacity development of the TBA is aimed at enhancing their skills in basic obstetric care, with a focus on the early recognition of high-risk pregnancies, and complications and prompt referral to the nearest primary health care (PHC) facilities [19,20].

Despite these efforts, delays in seeking and receiving appropriate care for complicated childbirth cases persist. Consequently, approximately 512 women per 100,000 live births and 72.2 infants per 1000 live births still die from pregnancy-related causes in Nigeria [21,22]. Evidence shows that delays in seeking and receiving quality treatment in health facilities under a skilled attendant contribute to these deaths[22,23].

Delayed referral is defined in this study as not referring immediately at the earliest sign of any high-risk pregnancy or complicated labour [19]. It refers to any lapse or postponement in the decision to refer a client who exhibits symptoms or signs indicating a potential risk or complication. Immediate referral is crucial to ensure prompt medical intervention and reduce the risk of adverse outcomes [19].

It has been argued that delayed referrals by TBAs to PHC facilities significantly contribute to the burden of pregnancy-related deaths in Nigeria [24]. Referral delays from TBA homes are reported to occur commonly, resulting in poor clinical states of patients at referral, reduced chances of survival and recovery, and increased mortality [24]. TBAs exposed to fewer training sessions have also been reported to have poorer referral practices with more delays compared to those exposed to more training sessions [19].

Other consequences of delayed referrals range from obstructed labour and birth asphyxia to infections in both mother and child, contributing to the poor infant and maternal mortality indices in Nigeria. Some causes of referral delays cited in LMICs include factors such as male trust and confidence in the TBAs, disrespectful maternal care in health facilities, infrastructural deficits, and cultural preferences [25–27].

This paper contributes to existing knowledge on the determinants of delayed referrals from TBAs to primary health care (PHC) facilities in Nigerian communities.

## Methodology

### Study design and setting

The study was a qualitative cross-sectional study, undertaken in three states from three different geographic zones in Nigeria. These were Kano (Northwest zone), Akwa-Ibom (South-South zone) and Anambra (South-East zone) states. The states were selected based on evidence of their having successful community health initiatives.

### Sampling and data collection

Two local government areas were selected purposively from each of the three states. To ensure contextual differences in perspectives obtained, one rural and one urban community were selected by balloting from each of the two local government areas sampled in each of the three states. A purposive sampling technique was used to select study participants who could provide rich and diverse perspectives on referral delays from TBAs. Participants were recruited and interviewed until saturation was achieved.

The study population comprised the health sector policymakers, religious and community leaders, and health workforce within the community health system in the communities studied. These included the formal health providers working within the primary health care system, and the informal care providers working in the local communities. Formal healthcare providers include doctors, nurses, community health extension workers (CHEWs) and other health professionals trained and recruited by the government such as lab technicians. The informal providers comprise TBAs, bone setters, patent medicine vendors (PMVs), herbalists and native doctors, and the intermediary health workers comprise community members chosen by the government from within the communities who have been trained to deliver primary health care services as community health influencers, promoters and service (CHIPs) agents. Service users (women and men) were also sampled for the study.

The participants were recruited and interviewed from the 10^th^ of October to the 28^th^ of October 2023. Participants included 73 respondents for IDIs and 12 community groups (of eight to ten service users each) of men and women for FGDs across the three states. The 12 community groups comprised six groups of men and six groups of women. Two FGDs (one in the rural area and one in the urban area) were conducted for men and another two FGDs were conducted in similar localities (rural and urban) for women in each of the three states. The research team was split into groups to achieve data collection concurrently in the three geographical areas. Table 1 summarizes the respondent category and interviews conducted by state.

**Table 1:** Summary of respondent categories and interviews by state.

Respondent category	Akwa Ibom	Anambra	Kano
Health program managers	2	2	1
Formal healthcare providers	4	3	4
Informal healthcare providers	13	7	11
Intermediary health workers	‐‐‐‐	‐‐‐‐	3
Private health sector	‐‐‐‐	4	‐‐‐‐
Community or Religious leader	7	5	7
(**FGD)** Community groups/ Service users (Women)	2	2	2
(**FGD**)Community groups /Service user (Men)	2	2	2
*Total (Males)*	*16*	*15*	*19*
*Total (Females)*	*14*	*10*	*11*
***Total per state***	***30***	***25***	***30***
***Grand Total***	***85***		

Data on type and cadre of health worker or role of the community member, patterns of child delivery in the community, extent of patronage of informal health providers, whether or not there were formal connections with informal health practitioners, referral patterns, and factors that promote or constrain referral from the informal health care providers (TBAs) to the primary health facilities, and reasons for delays in referral were collected through face-to-face interviews with respondents using focus group discussions (FGD) and in-depth interviews (IDI). The questions were open-ended and were accompanied by probes to elicit real-life experiences. This study is part of a larger study on community health systems in Nigeria. The study instruments used for the study were designed to elicit and understand the dynamic relationships within the community health system in Nigeria. The instruments were pre-tested for this study by researchers with years of experience in health systems research (S1–S4 Tables).

Written informed consent was sought for and received from the respondents (S1 Text). Interviews were conducted in neutral locations agreed upon by the research team and the respondents. Research assistants who were familiar with the local language took notes. Interviews were audio recorded and carried out in English, Igbo and Hausa languages depending on the preference of the participant. Interviews in Igbo and Hausa languages were translated to English Language.

### Data analysis

**Fig 1:**
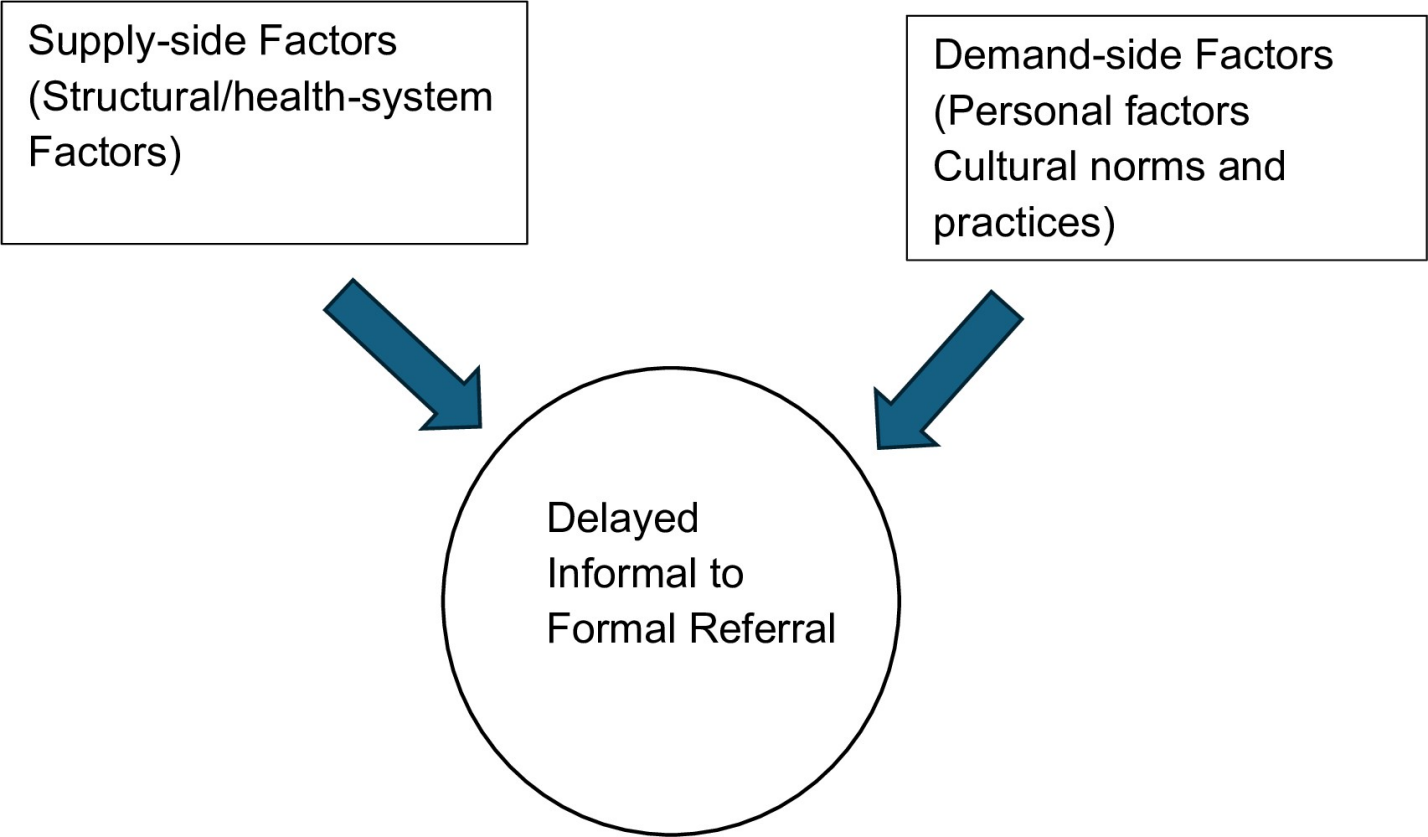
Analytic framework.

All interviews were transcribed verbatim and analysed manually using thematic analysis. Data analysis began by reading through the field notes and interview transcripts. The transcripts were read repeatedly to achieve immersion. Themes capturing key concepts were developed for coding and analysis. A codebook was developed and refined through discussions by team members to ensure agreement between coders. Child nodes and sub-themes were created from each parent node and theme. Analysis focused on exploring factors that led to delays in referrals from informal providers (TBAs) to formal providers. To achieve rigor in coding, the process of analysis involved double coding each transcript by two researchers and discrepancies resolved by meetings to achieve coder agreement and relevance of findings. The analytic framework (Fig 1) shows that there are two broad possible determinants of delayed referrals. These are supply-side (structural /health system factors) and demand-side factors.

The analytical framework was adapted from Ogu et al.’s adaptation of the Three Delays Model which contributes to maternal mortality as developed by Thaddeus and Maine [9,28].

Supply-side (structural factors): These describe those characteristics of the health system that affect the provision and quality of care given.

Demand side factors (Personal): These describe individual-level factors that affect a person’s demand for healthcare.

Demand side factors (Cultural norms and practices): These describe shared beliefs, value systems and customs that can influence a person’s decision to seek healthcare and accept referral for care.

The three delays described by Thaddeus and Maine are the delay in deciding to seek healthcare, the delay in reaching an appropriate source of healthcare, and the delay in receiving adequate and appropriate healthcare [28]. This adaptation focuses on the third delay which is the delay in receiving adequate and appropriate healthcare. This third delay accounts for referral delays. It describes the time a pregnant woman spends under the care of an informal care provider when she should have been referred to a health facility to receive adequate and appropriate treatment from a skilled attendant. The analytical framework was not pre-defined in this study, but rather were emergent themes during analysis which were then structured around the Three Delays Model.

### Ethics approval and consent to participate

Ethical approval was obtained from the Health Research and Ethics Committee of the University of Nigeria Teaching Hospital, Ituku-Ozalla, Enugu. Courtesy visits were paid to community leaders prior to community entry. Written informed consent was sought and obtained from every participant, and this included a detailed account of the study objective, procedure, and anticipated risks, and benefits. The anonymity of the study participants was ensured by removing identifying information. Confidentiality was ensured by proper storage of all data received and accessibility of the material only to the research team. The information on the participant’s right to withdraw from the research at any point of the study without consequences was also shared with the participants. All methods were carried out in accordance with relevant guidelines and regulations (declarations of Helsinki). All the data collected were stored on password-protected computers while the paper-based interview guides and consent forms were kept safe in a locked cabinet.

## Results

### Factors driving referral delays from TBAs to PHC facilities

These are presented in line with the factors that have been presented in Fig 1 above under data analysis.

### Supply-side factors

#### Infrastructural deficits and staff shortages

Referral delays were reported by respondents to have persisted despite training received by TBAs due to limitations such as the absence of a doctor in the PHC facility, locked PHC facilities at times of need due to infrastructural deficits and staff shortages, and lack of transportation to the facility when needed.

“There are several issues including water issues, electricity issues, and security issues. The lack of electricity is particularly concerning, as it prevents pregnant women from accessing the health center at night” [IDI, WDC Chair, Akwa-Ibom]

"Looking at our health center we only have two health care staff… just two, they cannot be here always, so we usually use volunteers."[IDI, community leader, Kano].

“When you get there now, you will only see a nurse there and the doctor might be working in Awka. He won’t be coming all the time. When you get there the nurse will be giving you first aid treatment while waiting for the doctor and the doctor might end up not coming that day. Will you say they are not there? But are you getting the service? So that is the challenge.” [FGD, Participant, Anambra]

“The health center should be improved to make it easier for us to want to go to the health center and we should also have doctors visit the health center on a regular basis. There are also challenges such as lack of electricity and water.” [FGD, Participant, Akwa Ibom]

#### Lack of incentives

Respondent accounts revealed that some non-governmental organizations (NGOs) supported TBAs in providing health services in the communities, by providing material resources and stipends for referrals (including when and how to refer their clients to PHC facilities). However, it was found that the support was not sustained leading to some TBAs resorting to their former ways and practices of handling patients completely on their own without referrals. However, there were accounts of TBAs who chose to support themselves; they formed associations that they used to control some of the practices of their members. One of the respondents revealed,

“I refer women who come to see me with complications, to the health centre; I don’t treat them. I can’t work on complications like if the baby is turned upside down in the mother’s womb; I just refer them to the health centre ….but yes there are some TBAs that don’t refer, let me say they have stopped referring or only refer sometimes.. They just continue to practice their art on their own, but we have association to control what we do” [IDI, informal provider, Akwa-Ibom]

### Demand-side factors

#### Preference for TBAs by community women

Respondent reports showed that TBAs were well known in their communities, and their services showed evidence of success, hence, women in the communities preferred accessing care with them sometimes due to the perception of a better level of attentiveness, preference for native medicines, or fear of injections causing them to refuse referral by the TBA until it is too late, sometimes leading to negative outcomes.

“You know because here people don’t like going to the hospital to give birth. They prefer TBAs, Therefore, these TBAs now have to care for them,”–[IDI, informal health provider, Kano]

“Some of the TBAs are very good and caring but we know some are quacks who cannot handle some cases…. but they are of use to us, mostly for people who are staying inside the cities, where the hospitals is not very close to them. So the TBAs are to be encouraged even though they have been taught when and how to refer”–[FGD, Participant, Akwa-Ibom]

#### Belief in TBAs for successful deliveries

Furthermore, some respondents reported that TBAs tend to have more patronage because they are religious and thrive on the religious biases of community members to improve patronage. Some community members consider pregnancy a spiritual event and hence believe they will only be able to deliver successfully under the care of a TBA.

*“*No, just this their belief, women are highly religious people, they sing songs and pray, with the psychotherapy of prayers,,,, some don’t even believe you can give birth under the care of the nurse, so some of them visit these TBAs, risking their lives and end up rushing to the nurses when it is too late and some end up dying” [IDI, WDC Chair, Anambra]

“I did not want to go to the health center…..I believe that if I go there no response or action would have been taken to assist us, so it’s better for us to stay with the TBA, it is only God who made the delivery possible for me” -[FGD, Participant, Akwa-Ibom]

#### Sense of superiority by some TBAs

Some TBAs were reported to feel more experienced than formal health workers such as frontline health workers in PHC facilities. Participants reported that although the TBAs create awareness of the need for antenatal and postnatal care in the health facilities, they still visit the health facilities to follow up with patients they refer there. They are said to visit the hospitals to monitor their patients and some who prefer to monitor their patients in the TBA homes do not even bother referring them to the health facility at all.

"We do go to the hospital sometimes to contribute with some information about what we know, we are professionals in the field, and we do provide emergency help if it is not beyond our control, and if the help is beyond our control, we refer them to the hospital…‥ [IDI, Informal provider, Kano]

Yes….some of the native midwives don’t even bother to send the mothers who give birth in their place to the hospital for proper care which sometimes results to the loss of the baby, but those of us that refer, the hospital workers use to be happy when we refer our clients to them, and that is why we have a linkage between us and the hospital, to avoid problems."-[IDI, Informal provider, Kano].

#### Male dominance

In some cases, cultural factors were also reported to play a part in referral delays, especially in Kano State where respondents reported that their main challenge with referral is male dominance, with husbands refusing to have their wives referred to health facilities for maternal care, even when the women themselves were willing to go.

"the main problem is noncompliance from the men in the community, in many cases, the women give their full support to us to refer them but their husbands prevent them from going, not even attending, even ante-natal or accepting immunizations for their babies" (IDI, Informal Provider, Kano)

#### TBAs feel entitled to deliver women within their communities

Respondent accounts revealed that some TBAs feel entitled to deliver women of their babies within their communities and sometimes undertake unconventional means to ensure that such women can only give birth to their babies under their service sometimes for financial gains. Such TBAs are not quick to refer women to health facilities when needed and even resort to unconventional means to ensure they get paid for their services. This practice also makes some community members prefer to deliver their babies in the TBA homes to avoid problems. These practices are known by people within the community and sometimes in cases of difficult or prolonged labour, TBAs are invited to the health facility to intervene in such deliveries, and most times, they succeed. A respondent recalled;

*“*…an instance occurred where a pregnant woman was brought into the health center and she was in labor for about two days, then when asked if she visited a TBA, she said yes, then we called the attention of the TBA, when she came, she used a broom stick from the health centre on the pregnant woman and the woman gave birth, we then paid her and that was it. However, we do always visit most of these TBA’s houses and lecture them, monitor what they do, and give out free kits if available to them given by (name with-held), although the free kits aren’t given anymore” (FGD Participant, Akwa-Ibom).

## Discussion

The availability of public PHCs at the grassroots in Nigeria provides last-mile healthcare and referral destinations for surrounding informal health providers. The current study affirms referrals from TBAs to PHCs, but with the caveat that a significant proportion of such referrals are delayed. Our study has provided an understanding around the demand and supply factors that encourage delayed referrals, typically resident in personal characteristics and relationships with TBAs, and systemic failures, respectively. These findings are novel to help health authorities in Nigeria begin to rethink incentives and disincentives that can spur accountable and patient-centred behaviours among TBAs with regard to timely referrals of maternal cases.

A major finding from this study driving delay in referral from TBAs is supply-side factors such as infrastructural deficits, long travel distances, and staff shortages. These situations despite recurring, continue to persist in LMICs such as Nigeria, hence delaying TBA referrals and fuelling the rejection of referrals from TBAs to PHC facilities by community members [29]. This is because TBAs, even when willing to refer, are aware that the facilities are either locked at the time, too far to access at the time of need, or do not have the right personnel in place to receive the patient, resulting in preference to continue managing the patient with limited resources and prayers [30].

The study also revealed that a lack of referral incentives and the need for financial gains hindered prompt referral despite previous training and collaborations. While several studies have reported some positive outcomes from training TBAs, similar interventions in other LMICs have suggested that capacity building alone is insufficient to improve the effectiveness of the health workforce [29,31,32] Strategies that utilize market-based approaches that improve incentives and accountability when combined with training are considered more successful [31,33,34]. In addition, strategies that involve the provision of subsidized drugs, health commodities, and increased regulatory oversight along with supportive supervision were found to be more effective [34].

The demand-side factors revealed in the study, including preference for TBAs, and the belief in their efficacy for successful deliveries, stem from TBAs’ residence within the communities. TBAs have earned the trust of community members by addressing common illnesses and reproductive health issues using prayers and herbs [16,33,35]. They are also easily accessible due to their proximity, comparatively cheaper costs of services, and flexible payment options [10,11,33]. Furthermore, community members generally perceive TBAs to be more attentive to their spiritual needs, which leads to delays or refusals to refer to PHC facilities until it is too late [36].

Male dominance, trust in TBAs, and a sense of superiority among some TBAs, who believe they are more knowledgeable than formal health providers were also identified as factors contributing to poor referral practices. These findings, which align with similar studies in Uganda, underscore the need for proper regulation of TBA health practices and improved health literacy among the populace [25,29]. In countries like Nigeria where some argue that TBAs fill an important gap caused by inadequate financial investments in health and mismanagement of resources, strong coordination and regulation of TBA activities are essential to ensure prompt referrals [10].

The non-conventional practices of some TBAs aimed at ensuring women within their communities are delivered by them were reported to be well-known among people, including health workers, yet, no measures were taken to dissuade such actions. These findings are similar to TBA practices reported in Ghana, where spiritual means were used to care for patients [37]. Although these practices may seem culturally acceptable to the community to allow it to persist, they bear grave health implications and require the intervention of health authorities and policymakers to prevent loss of life. In Ghana, TBAs are often initiated through dreams and revelations or by apprenticeship from family members who were TBAs and other non-family TBAs. They were reported to practice using both spiritual and physical methods, with practices founded on spiritual guidance, spiritual artifacts, herbs, and physical examination [37]. Practices such as TBAs’ delay in cutting of the umbilical cord and disposal of the placenta were associated with beliefs that indicated that improper placenta disposal, would have negative consequences on the child during adulthood. These spiritual beliefs pose threats to both the mothers and newborns [37].

While the qualitative study design employed in this research provided in-depth insights into the drivers of delayed referrals from TBAs in Nigeria, the findings (which may not be easily generalizable to other communities with different cultural, religious, or structural variations in their health system), can apply to communities with similar contexts. In addition, reasons for referral delays reported in this study were recalls from respondent accounts and may have been flawed by recall bias. Likewise, delayed referrals were self-reported by the respondents in this study, and difficult to ascertain if completely true or influenced by desirability bias. Hence, an ethnographic study that incorporates direct observation of TBA practices in the community and a quantitative study (which can be easily generalizable) would be beneficial. Furthermore, future research focused on developing and testing community-based interventions to enhance knowledge regarding the importance of prompt referrals and the development of sustainable strategies to encourage timely TBA referrals are necessary.

The themes around delayed referrals were not defined a priori; however, the emergent themes have also been identified in other contexts.

## Conclusions

This study has demonstrated that despite efforts at training and establishing linkages with TBAs, various demand and supply side factors continue to contribute to the delayed referrals of pregnant women from TBAs to PHC facilities for childbirth in Nigerian communities. It is important to address these factors to encourage institutional delivery with skilled birth attendance and improve maternal and infant mortality rates in the country.

Empowering community members to recognize and adopt behaviours consistent with healthy outcomes is essential [38]. Furthermore, health education in the communities, the provision of incentives to TBAs, adequate regulatory oversight and supportive supervision of TBAs are necessary to encourage prompt referrals and mitigate unhealthy TBA practices that endanger pregnant women.

Policymakers must legislate to address these drivers of delayed referrals through interventions aimed at improving health literacy within Nigerian communities. Moreover, the government needs to increase healthcare financing to address infrastructural deficits and manpower shortages. Policymakers should legislate for funding of undergraduate medical education and provision of attractive financial incentives for doctors who remain in the country and work in rural communities. This would help address manpower shortages in the health facilities. In addition, a formal recognition of TBAs within the health system, establishing a database of the TBA practitioners, providing training and re-training of TBAs in prompt referral practices, providing supportive supervision, and maintaining formal linkages between TBAs and the formal healthcare system can encourage timely referrals. Instituting control mechanisms to regulate and supervise TBA activities along with implementing punitive measures to deter unacceptable health-averse practices, is vital to ensure the safety and well-being of mothers and infants in these communities.

## Supporting information

S1 Table In-depth interview guide for formal providers. (XLSX)

S2 Table In-depth interview guide for informal providers. (XLSX)

S3 Table Focus group discussion guide for community groups. (XLSX)

S4 Table In-depth interview guide for community leaders. (XLSX)

S1 Text Information consent form. (DOC)

S1 Data Qualitative data -manuscript data. (DOCX)
