# Supplementary material for: Demand and supply side factors that drive delayed referrals from traditional birth attendants to public primary healthcare facilities: Insights from three states in Nigeria
Source: PLOS Glob Public Health. 2024 Dec 2;4(12):e0003886. doi: 10.1371/journal.pgph.0003886 (PMC11611091; doi:10.1371/journal.pgph.0003886)
Supplement: S1 Text — (DOC) [file pgph.0003886.s005.doc]

**Strengthening community health systems through multisectoral collaboration for health at the community level in Nigeria**

**Information Sheet and Consent Form**

**October 2022**

Community health systems (CHSs) mean the totality of interests in the healthcare access of communities. It is targeted at ensuring that healthcare gets the support of community members through making decisions, patronage, oversights, enforcing rules, among other functions needed to keep healthcare running in communities .In other words, this means that the community is placed at the heart of healthcare delivery. The structure on which community health systems thrive is the primary healthcare. Which is why most community health programmes work in partnership with the primary healthcare. Community health system and primary healthcare could be used interchangebly. Nevertheless, we realise that to sustain community health could be demanding, hence different sectors could come together to assist these communities. These sectors can include environment, non-government organisations, private individuals, water and sanitation, education, agriculture, power, works and housing, security, etc.

Hence we are carrying out this study to understand how community groups and the different sectors as mentioned can work or have worked collaboratively to strengthen community health. The study has received Ethical Approval from the Research Ethics Committee, University of Nigeria Teaching Hospital, Ituku-Ozalla, Enugu State, Nigeria.

**Informed consent**

The study has been described to me in language that I understand. My questions about the study have been answered. I understand what my participation is free will, and my identity will not be disclosed to anyone. I understand that I may withdraw from the study at any time without giving a reason and without fear of negative consequences or loss of benefits.

[ ] I agree to be audiotaped during my participation in this study

[ ] I do not agree to be audiotaped during my participation in this study

Participant’s name…………………………………….

Participant’s Organization...........................

Participant’s signature……………………………….

Interviewer’s signature

Date………………………………………………………….

Time interview started....................................

Time interview ended...........................................
