# Supplementary material for: Demand and supply side factors that drive delayed referrals from traditional birth attendants to public primary healthcare facilities: Insights from three states in Nigeria
Source: PLOS Glob Public Health. 2024 Dec 2;4(12):e0003886. doi: 10.1371/journal.pgph.0003886 (PMC11611091; doi:10.1371/journal.pgph.0003886)
Supplement: S1 Data — (DOCX) [file pgph.0003886.s006.docx]

**AHOP COMPARATIVE STUDY**

**OVERALL MERGED FINDINGS FOR REPORT AND PAPER WRITING**

| INTRODUCTION |
| --- |

Aloy

The respondents included a volunteer community mobilizer, two patent medicine Vendors, a local government engagement officer, a facility laboratory technician, a traditional birth attendant, an officer-in-charge, community leader, youth leader and a civil servant. And they were from Amawbia, Igbokwu, Okpuno, Gwagwarwa, Aka-Offot communities. Their experiences working in the community fit into different parent nodes.

Chinelo

There were two focus group discussions and 8 in-depth interviews conducted here. One FGD with men was in Kano and the other with females was in Akwa-Ibom. The in-depth interview on the other hand comprised of 6 men. The formal, informal and intermediate health care providers were duly represented from various communities in Anambra, Akwa-Ibom and Kano state.

Okechukwu

The Benefit of an improved health system especially in a developing society like Nigeria cannot be over emphasized, hence the imperative need for researchers to continually seek effective ways of improving and strengthening health systems both in urban and rural settings. While those in the Urban setting may tend to enjoy a considerable amount of improved health system as compared to their counterparts in the rural setting it is not surprising to see studies that target to improve the health systems from the grassroots. The goal of the current study is to find out ways through which health systems may be improved in the community level.The current study involved an in-depth interview with ten formal and informal community health actors across three different states in Nigeria (Kano, Anambra and Akwa Ibom). The study consisted of 7 males and 3 females: 3 respondents (Matron, Kumbotso Comprehensive health center; L.G.A Programme Director, Georgetown Global Health, Nigeria (GGHN); and WDC Treasurer) from Kano state; 3 respondents (Medical doctor, CSO, and policy maker) from Anambra state; and 4 respondents (Assistant Director of Works, Bone setter, OIC, and Red Cross Team Lead) from Akwa Ibom state. Their age ranged from 31 - 52 years.

Chioma

This study focuses on how community health systems can be promoted through multisectoral collaboration for health at the community level. Three states (Anambra, Kano state and Akwa Ibom state) were studied. The study consisted of 9 IDIs (5 males and 4 females): 3 respondents (OIC, CSO and religious leader) from Anambra, 3 respondents (Supportive Supervision Programme Officer, PMV and WDC Chairman Gwagwarwa ward) from Kano state and 2 respondents (State Nutrition Officer and PMV) from Akwa Ibom state. There was also an FGD (10 community women group) from Akwa Ibom state.

Enyi

The ten (10) transcripts analysed comprise a mix of respondent cadres from the three study states, as follows: 3 Community group FGDs (2 women groups in Anambra and Kano, 1 Men's group in Akwa Ibom) ; 4 Informal Providers ( trained Maternity propritress in Anambra, 2 TBAs in Kano and 1 PMV in Akwa Ibom) , 1 Formal provider (OIC in Akwa Ibom) and 2 policymakers (Anambra and Akwa Ibom). Findings from the interviews were thematically analysed using the Expanded Health System building blocks (Sacks et al)

Diuto

These transcripts (ten IDIs) comprise a mix of respondents from the three study states. A summary of 7 males and 3 females with ages 72,39,57,31, 37, 45, 39, 60,58,38. Three interviews took place :3 in Anambra State, 3 in Kano State and 4 in Akwa-ibom State. Anambra : 2 Community leaders (WDC chairmen), 1 private sector provider, Kano: 1 formal provider, 1 Community leader, 1 Intermediary healthworker, Akwa Ibom: 2 Informal providers (TBA, PMV), 1 Community leader: Chair person Health facilty committee chairman and 1 cordinator/head of Community and Social Development Project

James (by Tochukwu)

Transcripts covered include 01_AK_UB_FGD_CGW; 02_AN_RU_ID_IP-Anambra-Igboukwu-Native doc 2; 03_AK_UB_IDI_CL_WDC; 04_KN_RU_IDI_IP; 11_AN_RU_IDI_IP_informal lab; 14_KN_RU_IDI_CL; 18_AK_UB_IDI_CSO_FHI 360; 20_AK_RU_IDI_CL_Village Head; 20_AN_UB_IDI_IP; 26_KN_UB_IDI_FP

Tochukwu

Transcripts covered include: 06_AN_RU_IDI_CSO_ Peoples Mandate; 11_KN_RU_IDI_CSO; 17_AK_UB_IDI_CL_Village head; 20_AK_RU_IDI_CL_Village Head; 23_KN_UB_IDI_IP; 33_KN_UB_IDI_CL; 35_KN_UB_FGD_CGW.

Coding for these first batch of transcripts reveals a broad range of community health (CH) activities as well as multisectoral players and linkages. As the health system building blocks guided the coding discussions of salient points are identified.

Prince--

Casmir--

Mimi

Majority of the respondents were females for both IDI and FGD. Their ages ranged from 20 to 58 years with majority being in their thirties. They were a good mix of people from different occupations including both formal and informal health providers, public servants, business men and women, farmers, temporary workers and permanent staff of a company in the community, CSO, vigilante, and even a women leader. They had all held their position for at least three years for IDI, while for FGD, they all lived in the community at least 20 years.

Ugenyi

Respondents were a good mix of common occupations found in the respective communities spread across the three states studied as follows:

Akwa Ibom state – 3 Informal Health Providers (1 PMV, 2 Herbalists, 1 Bone setter) and 1 Women Leader.

Anambra state – 1 Informal provider (TBA), 1 Community Leader and an FGD comprising 10 males, aged between 29 and 60 years of age.

Kano state – 1 Intermediary Health provider (VCM with UNICEF), and 1 Bone setter

| PREAMBLE |
| --- |

Mimi

The general consensus belief is that health is important to the community and being able to own, control and direct health activities targeted at respective communities is considered optimal.

Ugenyi

There is on-going mobilization of the community members by informal providers, and this serves to provide sensitisation to health-related issues such as, compliance to routine immunization for vaccine-preventable diseases, malaria, typhoid, and high blood pressure. They also serve to dispel fetish beliefs as to the causation of diseases.

*“I help in mobilizing women towards acceptance of polio vaccine and routine immunization and other child killer diseases that affect infants that (are between) zero to 5yrs. So, we are here mostly to mobilize women because women are at the receiving end.......mobilize women because they are in the community, and the people know them.”* - 28_KN_UB_IDI_IHW.

| NATURE OF ACTIVITIES & SPONSORS IDENTIFIED |
| --- |

Aloy

The **government funded** most environmental sanitation, subsidized healthcare treatment and immunization activities. NGOs backed the state government on immunisation activities. The multinational agency were involved with sensitization programs on hygiene and nutrition, especially for under 5 children. They also sponsor and employ volunteers for routine immunization; ensure free antenatal and postnatal services for pregnant women. The multinational agency also organises pieces of training for healthcare workers. A particular OIC masterminded the revamp of the health facility in the community. Some committees on the other hand, collect donations and use them to support health facilities.

The **community** also organizes health programmes in which they invite medical personnels to assist their members. They provide free drugs and free medical care for those who need them. Some of these programmes could be sponsored by private individuals or philanthropists. The youths can also be organized to sanitize dirty areas every month.

Chinelo

***Government funded :*** The government-led initiatives could be in the form of distributing free health commodities and health promotion programmes. They can also be organized around politicians who intend to contribute to their community's development. However, some of the participants interpreted their initiatives to be politically motivated.

***Multinational agency funded:*** We recorded much evidence of multinational-funded projects at the community level. These projects include funding from Global Fund, WHO, and UNICEF. The funding of village community mobilisers (VCM) and Co-group by UNICEF and WHO is common. Their major responsibility is creating demand for health services.

***Private/Philanthropic:*** As for private and philanthropic-led initiatives, we found that private individuals organize health providers to conduct free medical care to community members as well as donate free medical products when the need arises.

***Community-based:*** At the community level, WDCs and other community leaders reported mobilizing their members to engage in sanitation, drainage cleaning/control and other health promotion activities. This is mainly championed by the village heads. For example:

*There’s what we call community sanitation in our community, I was part of it almost seven months ago in our area. They organize it under our village head at that time. They bring us out saying that those that are selling akara in the street near the convent should stop selling there, including the cooking and selling of food along the gutter (KN_FGM_UR_CGM).*

Okechukwu

Most of the community health system activities were mostly funded by the government and the NGOs. There are government funded health activities across various communities studied in Kano and Anambra states. The government funded health activities in Kano state include the distribution of free drugs; Government funded health activity in Anambra state include: national health insurance scheme, sickle cell program, distribution of medical products, immunization programme, nutrition programme, and family planning initiatives.

Health activities in various states were also funded by Institute of Human Virology, Nigeria (IHVN), World Health Organization (WHO), United Nations Children's Fund (UNICEF), United Nation Fund, United States Agency for International Development (USAID), Family Health International (FHI), The Challenge Initiative, United Nations Population Fund (NNFPA), Marie Stopes and Clinton Health Access Initiative (CHAI) etc. The funded community health programs by the NGOs include: the prevention of malnutrition, malaria and anemia in pregnancy program, immunization programme, HIV based intervention programmes, free health work force training on HIV and family planning, family planning initiatives, free distribution of drugs and medical equipment. However, Community health activity like the building of the antenatal and immunization center was also funded by a philanthropic individual in one of the communities studied at Kano state. A community-based organization in Anambra also funded the distribution of sanitary pads for the adolescent girls in the community.

Chioma

Most of the community health system activities were mostly funded by the government. The government funded community health activities in Anambra state include: immunization program, distribution of treated mosquito nets and family planning initiatives. In Akwa Ibom state, the government funded the Maternal New Born and Child Care Week program, and the expansion of the hospital at the community level was funded by the government in Kano state. Community health system activities in various states were also funded by WHO, UNICEF, Red cross, International Family health (FHI) etc. The funded community health programs include: Sessional Malaria Chemoprophylaxis (SMC), TB anti-process control program, Infant and Young Child Nutrition program. However, in Anambra state, some philanthropic/private individuals funded the breast cancer screening programme.

*Many people are involved in funding of community health activity like State ministry of health; WHO; UNICEF and many others. Even there are some individuals and some charity organizations that do help in funding some of community health* activities *(17_AN_UB_IDI_CSO).*

Enyi

*Government funded community health activities*

The government were involved in Health education, Nutritional education, Monitoring, Provision of grants to the poor and vulnerable,

*"I benefited from SURE-P. I had a baby that time. I didn’t really spend much during my antenatal, delivery and post-natal. I was given baby kit here. Sometimes they share malaria drugs." (14_AN_UB_FGD_CGW).*

Other government funded activities include, provision of free mosquito nets, medications, Sharing of Mectizan, Provision of injections, contraceptives and Construction of hand washing equipment using local materials. In Anambra state, there is this initiative by the government, ASHIA for sharing of Mectizan and polio immunization. In Kano, immunization, and distribution of malaria medication. In additon, the government has also implemented communty health activities in partnership and collaboration with Development Agencies like UNICEF, World Bank, WHO. Others include the CARTER Centre (for NTDs), AFRINET. The World Bank sponsors the Community Social Development Project (CSDP) in Akwa Ibom state,They embarked on community led projects in Health, Water and infrastructure. The UNICEF sponsors majority of the immunization programmes across the study states, and in Kano the Volunteer Community Mobilizers (VCM) programme.

*Multinational agency*

VCMs were sponsored by NGO (UNICEF). In Nassarawa LGA,Kano, Mobilisers move from household to household creating awareness on the dangers of delivering babies at home, enlightening and educating pregnant women about the importance and benefits attached to attending ante-natal care. In cases where the pregnant women could not make it in time to the hospital during labour and deliver at home, they give immediate care/help to both mother and baby before transporting them to the hospital for further examination. Also Mobilisers are involved in the upcoming hepatitis vaccination. In Akwa-Ibom, communities were supported by the World Bank to ameliorate the effects of the pandemic on the lives of people.

*Private and Philanthropic organisations*

Some private organizations and individuals (politicians predominantly), have also individually or in partnership, and as part of their corporate social responsibility. Politicians in Anambra State have sponsored a number of community projects , including health insurance enrolment of community memebers. In Akwa Ibom state , the Oando Oil company sponsored an Education and empowerment programme, focusing on women, children and out of school girls.

*Community based:* A community -driven initiative involved awareness creation for personal hygience during the COVID-19 pandemic, was implemented in Akwa Ibom state during the pandemic.

*NGOs:* A number of NGOS have also sponsored a number of other community health activities, especially in Akwa Ibom State. In Aka-Offot, Uyo, Akwa-Ibom, they provide health education and measurement of blood pressure.

Diuto

*Govt. funded:*These include immunization, community drug distribution, COVID-19 vaccination, COVID-19 palliatives, targeted measles vaccination campaign in Anambra State. In Kano state, there is the COVID-19 vaccination, HIV programme. There is also a health insurance contributory programme, distribution of mosquito nets. These are all government funded programme

*Community based* activities in Kano state to improve health include Cleaning of drainages, spraying of insecticides and fumigation and general environmental sanitation . Also, Immunization, Community mobilization and awareness creation, Free medical outreach- treatment of ailment and Provision of free drugs

Multinational agency funded- Provision of Mectizan, mebendazole, Immunization/Vaccination, NGO led community health programs in Anambra state include drug administartion for tuberculosis and leprosy .

NGO led programmes in Kano state include HIV programme ( collaboration of government and NGO) offering free HV treatment, other free outreach programmes.

The VCM programme in Kano which encourages and mobilises pregnant women to access facility based maternal services was initiated by and NGO in collaboration with UNICEF

In Akwa Ibom the FHI has provided free HIV treatment to communities and the Red Cross, in the process of catering for Cameroun refugees have contributed to the community health through various activities

The health projects were sponsored by the WHO, UNICEF, ASHIA

Private/ Philanthropic- there was a time E. O through the Rotary Club sponsored some projects in the community

James (by Tochukwu)

Different health systems building blocks for community health systems were highlighted across transcripts including health workforce (both formal and informal), medicines/drug supply, community participation and engagement, financing, leadership and governance as well as multisectoral engagement.

In Akwa-Ibom, women expressed appreciation for the free malaria treatment and eye check-up programs, which were beneficial to the community. They also reported personal support offered to fellow community members to direct them to formal facilities for care. CSO/NGOs contributed to HIV and Tuberculosis prevention and treatment programs in Akwa-Ibom, educating communities on adherence to medications, record drug intake and monitor treatment, and organize adherence counseling for clients who are not faring well. They also follow up with clients to ensure that they are retaining their drugs. In Kano, antenatal care and tuberculosis were some of the health conditions targeted. Sometimes philanthropists or politicians float and support programs: “*someone in the community, the philanthropies who now have the will to help the community in terms of their health and wellbeing. He will now gather a program or sponsor a program, health program that will now benefit the community… The eye program is about screening the community in search of those with eye problems. Some will do screening if they have simple glaucoma a drug will now be prescribed for them. Some may be prescribed reading glasses to improve their sight…*”[IDI, formal provider, Kano]

Tochukwu

CH programs were largely discussed from an experiential point of view. Respondents highlighted several programs that originated from diverse sources: government, multinational agencies, philanthropic projects and communal actions that align to health goals. CH programs included: “*distributing mosquito nets, and contributing money and resources to building a small clinic in the town*” [IDI community leader, Kano]. Other key points was the organization of programs and outreach events to educate people about health issues and providing traditional medicine for certain ailments.

Conversations also suggests that the community's attitude towards health care is positive and that they are willing to participate in community health care activities. The community's involvement in healthcare has been made possible through the efforts of the ministry of health, which provides awareness and support for health initiatives. Respondents mentioned several programs on different diseases, including tuberculosis, some culture specific syndromes ‘Tundurmi disease’, dog bites, and breast cancer. The programs are initiated by the government and other organizations, and there are both health workers and volunteers involved in almost all of them. The members describe the initiatives as having yielded positive results, including reduced mortality rates among pregnant women and under-five children.

Prince

The government was found involved in community health projects. Four major programmes most mentioned include free health interventions for elders, even though not regular, family planning, nutrition programmes for malnourished children, and immunization. The frequency of these programmes was not extensively discussed, but participants highlighted reasonable extent of community participation. Also, notable organisations were found doing community health programmes, leveraging supports from community people. These organisations included WHO, UNICEF, Red Cross, among others that participants could not mention. The programmes they supported traversed free health services to displaced people and scaling up uptake of formal health services by liaising with informal providers, community leaders, and the general community populace.

Some individuals were found involved in community health. Informal providers provided free health services for the indigents and other members of the communities, politicians rolled out free eye screening and treatment services and equally built health infrastructure, and some members of the communities knowing the importance of education to hygiene and health, helped to sponsor the education of children of indigent parents.

Casmir

NGOs and government collaborated to fund health activities in Panshekara community Kumbotso Local Government Area of Kano State. The multinational agencies like WHO, UNICEF, United Nations funded health related projects in Kano and Akwa Ibom states in the areas of free drug and medication for refuges, provision of water, renovation of health centres etc. Specifically in Akwa-Ibom, the Red Cross was seen providing healthcare to refugees from Cameroun. There were visible records of private/philanthropic supports towards healthcare through rendering of free medical services in Igboukwu community, Aguata LGA, Anambra State and sponsoring of health programmes in Gwagwarwa community, Nasarawa LGA, Kano State. There were noticeable community based health activities going on in Gwagwarwa community, Nasarawa LGA Kano State such as the TBAs educating women on the importance of antenatal care etc. In Anambra, we found a community-based health insurance scheme ongoing, where well off community members contribute finances to provide healthcare for the community people. A programme managed by an NGO in Kano identified and treated tuberculosis cases through the use of PMVs. Finally, some PMVs in some of the communities provided free drugs to community members, usually during festive periods. The roles played by informal providers in community health education were discovered as well.

Mimi

***Government funded:*** There were some government-funded health activities going on at the community level. Informal providers such as TBAs are also being engaged more in "formal" linkages with the existing health system. In one of the communities, the introduction of VCM for immunization, antenatal outreach, and family planning were credited to government interventions. As well as provision of materials. Another community praised the government for doing a good work as is expected of them and also collaborating with multinational agencies for the benefit of the citizens. Another community mentioned that immunization activities and sharing of mosquito nets were also funded by their government and the process was very transparent.

***Multinational agency funded:*** Multi-national agency funded projects can still be found in some communities. Examples of these agencies are the UNICEF and FHI-360. Activities range from distribution of mosquito nets to deployment of VCM. It was also noted that VCM is sponsored by UNICEF which made the community more receptive to their services. Multinational agencies also collaborate with state governments to carry out CHS activities. Apart from UNICEF's VCM, another group is the CO - group and the dietary nutrition also sponsored by UNICEF. The later provides education to the community on nutrition and balanced diet, and growing food from their backyard. In another community, HIV counseling and testing activity, TB and measles campaigns, immunization and sharing of mosquito nets, were some of the activities credited to NGOs like WHO in collaboration with UNICEF, Red Cross, FHI and Alpha Net.

***Private/Philanthropic:*** Informal providers were found to be mainly privately-funded and some philanthropist funded community health activities were also identified. Some private individuals like doctors were also noted for providing free medical services in one of the communities. Another community was privileged to have a philanthropist who organizes medical outreach on a yearly basis in collaboration with the Anglican church in his community. Some other churches equally organize outreach programs once in a while.

***Community-based:*** The community-based interventions/activities appeared predominantly driven by indigenes of the respective communities and were mainly organized to take place annually at about the same time of the year. Some community-based interventions included providing health and sex education for women in particular, courtesy of the women leader in one of the communities. Informal providers were also responsible for providing traditional medicine for malaria and pile cases which are endemic illnesses in the community. They also refer to the hospital if the sickness defies treatment. Another community had environmental and health unit tasked with health promotion, as well as a yellow fever campaign activated by the community in order to vaccinate community members. Another respondent in one of the communities mentioned that their community pays maximum attention to community health and constantly organize programs for health education, outreach, etc.

Ugenyi

***Government-funded:*** Government-funded initiatives in the communities are mainly carried out by staff of the PHC and volunteers selected from the community. These initiatives were however, not found to involve the informal providers, such as the bonesetters and herbalists.

*“There was a time a group of people use to come, they were neatly dressed with their ties well knotted and carry bags like your own. They said they came from the Anambra state (ministry of) health, registering people from the health center for immunization.”* - 04_AN_RU_IDI_CL.

*“....polio immunization, measles immunization, sharing of mosquito nets and fumigation, HIV/AIDS Programme, and sharing of condoms with free HIV/AIDS tests, just to mention a few. R1:We just finished the River Blindness project. Mectizan was given and it lasted for seven (7) days. The nurses went to schools, churches, village square, even to household to administer it”.* - 13_AN_UB_FGD_CGM (R1)

*“There is an activity we just concluded on river blindness disease.”* - 13_AN_UB_FGD_CGM (R2)

***Multi-national agency-funded:*** The multi-national agency funded health initiative identified was targeted at improving the acceptance of routine immunization against vaccine-preventable diseases among mothers in the community.

*“(UNICEF) helps in mobilizing women towards acceptance of polio vaccine and routine immunization and other child killer diseases that affect infants that (are between) zero to 5yrs. Yeah, there are so many organizations like WHO doing something like what we are doing.”* - 28_KN_UB_IDI_IHW.

***Private/Philanthropic funded:*** Private/philanthropic funded initiatives were mainly funded by the respective community's members in diaspora associations and involved periodic health visits by a team of medical personnel to perform health checks on members of the community.

*“Some of this medical treatment is offered by the community members abroad. There was a time that one of our sons came back from oversea and was sharing face mask that was during the time of covid-19. Sometimes in December, they normally announce for free medical checkups sponsored by A*** People in Diaspora and A*** Development Union. They give people drugs for eye problem, rheumatism, cataract and so on.”* - 22_AN_UB_IDI_IP.

***Community-based-funded*:** Community-based initiatives ranged from alliance/collaboration with the diaspora associations to utilising community structures such as the women's group to organise health talks for members of the community.

*“..... including organizing meetings for women in order to give them good advice and talk about their problems. I also organize contributions from women for anyone who can help a person financially or otherwise........We mostly talk about health to get to know people who are sick and advice people to check their blood pressure regularly at any pharmacy or health center. I also mention the health center, where they can go for treatments, tests, and to have their blood pressure checked. This community is surrounded by three health centers: A******, E*******, and B******. I always make it a point to advise them to go to the nearest health center for regular checkups and treatment” -* 07_AK_UB_IDI_CL_Women leader.

**FINDINGS ON ANALYTIC THEMES (PARENT NODES) BASED ON THE SACK’S EXPANDED HEALTH SYSTEM FRAMEWORK**

| Household Production of Health and Social Determinants of Health |
| --- |

Aloy

-Some groups or households came together to contribute money to assist those that are not able to afford treatment. There were also discounts for health centre furnitures by the carpenter.

Chinelo

Households frequently engage in sanitation and drainage construction as well as the clearing of bushes as a way to promote community health. Community health workers are sometimes contacted to give health advice to households whenever they are sick.

Okechukwu

Findings shows that the most engaged activity was found to be on sanitation (3 of the 4 responses) which was observed majorly in Kano State and the remaining was on security which was observed in Anambra State.

Chioma

In Anambra state, borehole construction was funded by the politicians and in Kano state, the communities were involved in environmental sanitation such as cleaning of the drainage and some were further involved in donation of nutritional items to malnourished children. *We do sanitation, we come and do some self-hand work to clean the drainage (33_KN_UB_IDI_CL).*

Enyi

Households produce health through engaging in healthy practices learnt from community health education e.g safe water drinking, accepting immunization, proper nutirtion etc.. the community acknowledges that these practices improve health and other social determinants of health ; “...*But when you teach them through health education or even through immunization, that this is what causes this sickness don’t do it. And when the person doesn’t fall sick and healthy, the person can go anywhere (farm )and be productive ..... Yes, I mentioned nutrition because, in the course of giving them health education you will also tell them the nutritional value of eating healthy food (that’s balance diet) and not eating one particular kind of food.” (26_AN_UB_IDI_PM).*

Community health education has also helped in dispelling some cultural nutironal beleifs, fpr example denying children egg, to prevent them from stealing. In communities where the government has taken over their lands, they are no longer able to cultivate and this negatively impacts household resources; *“Back in the days, we could farm palm trees as a community and use the proceeds to cater to our needs. But we don’t have the lands again because government has taken over them .” (02_AK_UB_FGD_CGM-R2* )

There is a high level of awareness by communities on what SDH impacts household and community healthand usually identify this when engaing with various potential funding authorities. In Akwa Ibom state, *“Three communities have identified water and also healthcare. The health centre goes with complementary water and equipment....” (11_AK_UB_IDI_PM_CSDP)*

Some communities also feel that lack of good access roads impact the health of their pregnant women during labour, with the result that some have delivered on the road to the hospital when they couldn’t get to the hospital on time.

Diuto: Spraying of insecticides, Environmental sanitation

James (by Tochukwu)

Community members especially women reported supporting and directing their fellow community members where they could access adequate care. In Akwa-Ibom a woman reported that “*I recommend that the baby be brought to the health center for immunization after birth. Sometimes I bring the pregnant women by myself to the health center for her to be attended to especially during odd hours of the night, I also look out for people who are sick and make them to visit the health center for treatment*.” [FGD, female, Trader]

*Actors:* Different actors were identified within the community health systems space including formal health workers in health centers, doctors who come to educate and give care to women, patent medicine vendors, traditional medicine practitioners. Also, community leaders, such as village heads, youth leaders, were identified to play active roles in contributing to the local health system. NGOs/CSOs were also reported to provide and support health activities in the community.

Prince

Households indulge self-medication as reported. This could be because of unavailability of formal care close to them or that the PMVs around them do not demand for diagnostic reports from medical laboratories before administering care to them. Self-medication has evolved into a norm in the communities. Also, informal healthcare practices were resident within households. Children learn the occupation from their parents, and they sustain the practice by passing it down to the next generation.

Casmir

It was observed that households indulged in self-medication. This could be as a result of unavailability of formal health provision in the communities of the study states.

Mimi

Enforcement of environmental sanitation and hygiene was the most common form of community production/social determinants of health. In one of the communities, the community leader was responsible for ensuring that people adhere to environmental sanitation regulations.

Ugenyi

Behavioural Change Communication interventions were carried out at the community level and were geared towards immunization, environmental sanitation, health seeking behaviour and appropriate preventive practices against malaria and STIs.*“We change their behavioural thinking to accept vaccination and to immunize their child as at when due.”* - 28_KN_UB_IDI_IHW.

*“....in terms of environmental sanitation, which is health issue as well, because prevention is better than cure.”* - 02_KN_RU_IDI_IP.

*“Talking about sanitation, during the previous governor’s regime, he provided container to dispose wastes in street, but now, instead of the present governor to continue, he is going house to house to share sanitation bills, we know that clean environment is good.” -* 22_AN_UB_IDI_IP*.*

| Health Workforce |
| --- |

***Formal providers***

Aloy - The various cadres named were the OIC, CHEW, food science officer, health attendant. There was also a respondent that described them as temporary and permanent staff. The mode of recruitment recorded here involved the use of a statement of result to analyse the school and ensure it is a genuine school and to assess if the individual is qualified. They also attend health education training. The mentioned available resources to work with were the doctors and nurses that volunteer for free, though they were given stipends for transport. But it is rarely enough so staff are not recruited on a permanent basis but as volunteers, and to receive meagre salary or in some cases, are contracted for free.

The engagement with the communities involved the use of mobilizers in each settlement. The village town criers also help to disseminate information to the community. This is also backed up by the WDC. The aim of these is to ensure awareness and avoid resistance by the community**.** One common health programme at the community level that formal health providers help in is the immunization of children. Formal providers use this opportunity to not only immunize children but identify malnourished ones among them and educate their parents on proper dieting.

Chinelo

*Types and cadres of health workers*

Community health extension workers (CHEWs) who are formally trained to provide health services are the common formal category of workers in the community health system.

*Mode of recruitment*

They are engaged in PHCs and must have been certified by the primary healthcare board to provide health services.

*Available resources to work with*

There were report of inactive staff at the PHCs and report for them to be sanctioned may not be adhered to.

*Engagement patterns*

They receive trainings from government and other multinational agencies to help them in the discharge of their duties in primary health centres. An example of the training is the HIV and FP services but we found that most times it is not all the staff that is trained.

*Engagement with community*

Though CHEWs are primarily in PHCs, they are also in health promotion at the community level. An example of their activities at the community level is promoting awareness about diseases like Covid-19. They sometimes work closely with community-based health groups like VCM.

Okechukwu

Different types of formal health providers (Nurses and health supervisors) were involved in the community health programs***.***

Chioma

In this study, we found different formal health workers (health supervisors, OICs, LGA nutrition officers and family planning provider) who promote community health activities. In Anambra state, we found that some of these formal health providers were recruited by the NGOs from the National down to the local government level. The formal health providers worked with the members of the communities to facilitate communities’ health. For instance, in Anambra state, it was found that the formal health providers worked with various groups in the community (like the Ward Development Committees (WDC), Community-Oriented Resource Persons (CORPs), Community Engagement Focal Persons (CEFPs) and CHIPS in order to promote community health. In Kano state, the community members often invite the formal health providers whenever they want to carry out meetings or seminars related to health. While in Akwa Ibom state, the formal health providers work with members of the community as that is the only possible way for the people to accept any health program initiated at the community level like the family planning programme. The formal health providers across the various states studied received proper training before they could render services to the communities which promoted efficiency as regards to community health service delivery.

*We have people in the communities we work with like the WDC. We have the community-oriented resource persons, the corps, that we use to carry out family planning activities. We have the CHIPS in the wards we work with. In this ward, we have ten CHIPS and two CEFP that divides the activities. We have their supervising roles and the officer in charge (09_AN_RU_IDI_FP).*

Enyi

Types and cadres of health workers of formal providers include Medical Doctors, Nurses, community health workers, health assistance, midwifes. They are recruited by the government. For a number of government driven programmes for community health, which are predominantly immunization and distribution of drugs and medical supplies, activities a mix of providers, doctors, nurses who are facility based and community health workers, assisstants (trained and untrained) who are mainly based in the community. Formal providers include doctors, nurses, midwives, CHEWs, JCHEWs.

These are usually *recruited, trained and deployed* by the government

***Health workforce Engagement patterns with communities***

The workforce engages the community throgh differents patterns and avenues. For community entry, it is not uncommon to approach the traditional head through the president general (PG) of a community, to seek approval and buy in for a given community health programme.

Programmes are then carried out in the health facilities. In addition, where feasible, outreach programmes are taken to churches, schools, village squares and markets, as deemed appropriate.

The annual women August meeting in Anambra state has also become a key engagement avenue for health education and other activiities. The following long qoute from a policymaker, reflects a typical community engagemnt pattern in Anambra state, before and during community health programmes ,

“...*Everyone knows their limitations and boundaries, that’s how it is be done. And on the side of community, the Igwes and PGs also know their own. Like the measles programme that is coming up, we have reached the Igwes, the policymakers at that level, the market women. By advocacy visit, meetings, the OICs conduct meetings with them, we have just concluded the meeting of communities with the OICs. The OIC in charge of a particular community will have the meeting with the community heads to involve them in any campaign that’s about to happen before the town crier goes into the community to announce that this programme is coming up from this date to this date, also the radio jingles is also involved, but before that the Igwes and PGs are informed that such programme are coming up so time, so that when the immunization people come in for supervision of something they wouldn’t say that they didn’t know about the programme. They are fully involved*

Diuto

*Formal providers;* Across the three states, these include doctors, nurses, OIC, lab. Scientists, hygienne staff, CHEWs. They are usually recruited by the government but occassionaly by NGOs for specific programmes. They are all usually trained prior to employment but also recieve on the job training and programme specific trainings when available, to guide them on the application of medical items and services.

*Engagement with informal sector:* In Akwa Ibom state , formal health providers invite and train TBAs at the facilities and engage them regularly on appropriate and hygeinic practices, because they (TBAs) have high patronage from the community women. They also advise them on the importance of encouraging their clients to attend the health facility for deliver.

In Kano state, health workers encourage the women who attend facilities to educate and mobilise other women

*Community engagement*: A typical engagement pattern for community health programme in is as reflected by this WDC chairman, *“when there is any health programme, the OIC will call me as the WDC Chairman, I will then meet here to discuss, after that I will call the town crier who will announce to the community members and all of us will work with community to ensure the programme is done. That is it. (15_AN_UB_IDI_CL_WDC*). Additonally, , health workers also engage the communities through various community heads (traditional and religous)

James (by Tochukwu)

Formal health providers were acknowledged across transcripts, especially doctors and PHC workers. Former providers in PHCs superintend over activities in PHCs and are critical in anchoring and implementing programs originating either from the government, NGOs/CSOs or even private individuals. During the distribution of the TB cough medication and others, the formal health workers attached to the community health centre, as well as workers from the ministry worked together that provided health services in the Kano communities. There were however concerns that formal health workers were in short supply: "Looking at our health center we only have two health care staff… just two."[IDI, community leader, Akwa-Ibom]. In Kano a formal health worker identified the shortage in health workers and identified that majority of the staff in his facility are volunteers: “*Actually, the ratio of workers to patients is low actually. Even though, because most of the workers are volunteers, they are volunteers*…” [IDI, formal provider, Kano]. This creates need for the mobilization of resources to improve health. Health programs/packages from the government ad CSOs/NGOs come in to fill in community health system gaps.

The conditional preference for some informal providers were highlighted. The common practice of using patent medicine vendors as a first response without undergoing tests; and the preference for traditional medicines because of needle phobia were highlighted - practices they acknowledged to be dangerous was highlighted in Akwa-Ibom “*Most of them use traditional medicines such as a mixture of lemon grass water or soda water or palm wine too with Lipton tea to keep them going for a while. Also most persons prefer the native medicines because they are afraid of syringes in a case that they are administered injections*.”[FGD, female, trader, Akwa-Ibom].

Tochukwu (merged formal, intermediary and informal)

Formal, informal, and intermediary providers were reported across transcripts. Formal health workers in PHCs supported by the WDCs are recognised to be highly regarded as a place to receive care: “*first we have the nurses who are in charge of health-related problems, we have WDC they see to the welfare of the hospital and seek help from the community for hospital progress*” [IDI informal provider, Kano]. Sometimes, in some programs, the formal and informal providers collaborate in health programs. As was the case in Kano where birth attendants were trained by multinational agencies to complement the activities in formal health provider settings. Volunteers and TBAs were trained by experts from the donor agency during the CHILD and other health related programmes to reduce stillbirth and mortality rate in Kano State.

Although community members were not always clear about the category of formal workers involved in CH programs, they were often able to situate them within their work spaces, or identities in the community. The health workforce building block is represented by the doctors, nurses, and other professionals who are brought in for medical outreach programs. The conversations also highlight community members who are known for providing traditional medicine and treating dog bites. Formal providers included, majorly community health workers in primary health centres in communities who were often agents in CH programs sponsored by government and multinational agencies. One unique pool however was the nature of health workers attracted and utilized by Philanthropic funded CH programs; who were unique in the sense that they were often able to pull human resources for health across places and especially indigenes in diaspora as well as health care providers in communities. This was particularly common in rural Anambra communities.

The WDC was identified in Kano to work hand-in-hand with the health workers in the health facility to assist them in providing health services to the community. They also engage with traditional birth attendants to ensure that pregnant women and children receive routine immunization: “*the community, the village head, WDC,…I think WDC, and VCM. Then also in attendance are the village head and some community members….host meetings…every month. Any day we have combined meeting with WDC and TBA, the in charge is always there or vice or focal person*.” [IDI, informal provider, Kano]

Sometimes, in communities health activities are carried out on a voluntary basis and are free of charge. The community is motivated by a sense of responsibility to contribute to the well-being of their fellow citizens. They work closely with government health services and non-governmental organizations to ensure that the activities are carried out effectively. The community's efforts are focused on creating awareness and providing resources to help combat health problems in their area.

Some concerns raised about the formal health workforce include the issue of health worker shortages. The government, through the ministry of health, was seen not be doing enough to address the issue. Community leaders raised the issue with the ministry and even wrote to the permanent secretary to draw attention to the problem. However, the shortage of manpower remains unresolved.

*Formal connections with informal practitioners*

There was a connection between formal and informal health providers in Kano where TBAs move from house to house to encourage pregnant women to go to hospitals for medical attention. **I**n Kano State, VCMs register childbirth and death; also created awareness on antenatal care; PHI and Podium monitor tuberculosis and malaria cases and provide free drugs, nets and TBAs create awareness on antenatal and post-natal cares to women. Anambra and Akwa Ibom have just youths who participate in sanitation only.

Informal health providers in Kano all refer patients to the formal health providers. However, records from Akwa Ibom and Anambra do not indicate any form of referral.

Prince

Community health extension workers (CHEWs) and community health officers (CHOs) are the most common formal workforce within the community health system. Whenever external health programmes are brought to communities, the health workers within the communities are recruited to join the health workforce brought by the programme. Also, fresh graduates as CHEWs, CHOs, or even Nurses are taken as volunteers, with plans for future permanent employment. In Kano, to be recruited as a volunteer, one needs to apply through the primary healthcare development agency. This was not the practice in other areas, as recruitment of volunteers rest within the powers of the OIC. In the event of external community health programmes needing specific kinds of outputs, those that are recruited are provided specific kinds of trainings, and are given stipend. WDCs could be asked to recommend persons that can work with the organisers of external health programmes. Usually, they recommend such persons from within the communities.

Casmir (formal, intermediary, informal merged)

In Kano and Anambra states, there are trained and untrained formal and informal workers who render health services along with the OIC to the community members. The Kano State government through ministry of health invites the health stakeholders for briefing on new health programs. This is done in Panshekara community, Kumbotso LGA, Kano State.

There is a laid down criteria for selecting and recruiting volunteers by the NGOs in Kano State. There is also engagement of community members in screening of patients to know if they are malaria positive in their community. There is presence of health intermediaries such as volunteers in Kano State. The health intermediaries are recruited through the primary healthcare coordinator’s office and cleared by the Nasarawa Local Government Area, Kano state. These are specifically visible. In Gwagwarwa community, Nasarawa LGA, Kano state, there are traditional and spiritual healers providing community health services. Community volunteers and mobilizers are recruited through the community leaders. TBAs meet regularly and attend to community members. In Kano, Anambra and Akwa Ibom states, there were trainings organized for the informal health providers such as TBAS, and others on surveillance. Bone setters also train members in the skill. In Gwagwarwa community, Nasarawa LGA, Kano state, local midwives are selected to work with the formal health system. Informal providers are being engaged with formal providers in Anambra and Kano states through offering of free medical outreach and doing volunteer works.

A common trend found in communities is the sustenance of the craft of informal providers. For most, especially the bone setters, herbalists, and other traditional practitioners, the craft resides within families, and usually not transferred to those outside the families. The TBAs had an apprenticeship system, and so the PMVs. Usually, the TBAs learn how to undertake deliveries of children from more experienced TBAs or from retired nurses and community health extension workers that are resident in the communities. Doctors were said to be present at the level of primary healthcare in the communities, unlike other study sites where there is reliance on doctors provided by ongoing programmes. That means, if the programmes end, the doctors will leave.

Mimi

*Types and cadres of health workers:* Cadres of health workers that dominated the health workforce for formal providers were mainly nurses, and CHEWs, with a very small number of doctors. The health workforce consisted of CHEWS/JCHEWS for immunization and antenatal in one of the communities. A respondent in another community complained about the issue of manpower shortage leading to community members taking up some of the work. However, this issue of staff shortage seems to have become a thing of the past in another community courtesy of the primary health care development board, who recruited more staff and even volunteers. This was also the case in yet another community were the staff mix included both health workers in the facilities and community-based ones.

*Mode of recruitment :*Formal providers were mainly found in the government-owned health facilities and as such were recruited by the government and distributed according to their specific area(s) of expertise/specialization. The respondents acknowledged that health providers are well trained and doing their best, and they had no problem with that, and sometimes NGOs offer retraining programs to them. The community-based health workers were selected based on wards in the community.

*Available resources to work with:* Three FGD participants in one community all acknowledged the improvement in hospital infrastructure and available resources like wards, laboratory, labor room, treatment room, etc.

*Engagement patterns:* Strong engagement existed among the formal providers and the informal providers as well as members of the respective communities. An FGD participant was impressed by the improved engagement pattern through outreaches for immunization making it easier for them to access the services.

*Engagement with community:* The women leader in one of the communities noted that they are always asked to visit the health centers for health lectures, which most women do not attend. A respondent in another community noted that the weekly outreaches have been beneficial in decongesting the health facilities.

Ugenyi (formal, intermediary, informal)

***Formal providers***

**Types and cadres of health workers:** The formal health care providers present in the communities consist mainly of the OiC and other staff employed at the PHC.

***Health Intermediaries: Mode of recruitment*:** The health intermediaries were mainly community members nominated by the rulers or community into their respective positions of service.

***Informal providers-Mode of recruitment*:** The informal providers were found to have either been born into the lineage for their respective professions, rather than recruited.

*“It’s not a kind of job where training is necessary. It must be bestowed upon you. So, if it is not bestowed upon you, it won’t work.” -* 05_AK_UB_IDI_IP_Herbalist

*“Mostly we inherit the work ...... apart from inheritance people don’t use to get into the work just like that.”* - 02_KN_RU_IDI_IP.

***Community-based health groups:*** Although, informal providers generally enjoyed a good relationship of trust with the community leaders and members, a few of them found that they were better appreciated/valued by people outside their respective communities.

*“They (the community) welcome me warmly because I do my job well. So many people, both inside and outside the community, are referred to me by my community members or people I've treated. I get along well with them, and they get along with me because I was a village council secretary, and my village respects me because I'm submissive.”* - 01_AK_RU_IDI_IP_Bonesetter.

*“I don't have many people to treat in the community; I mostly treat outsiders, and I treat them well.”* - 22_AK_RU_IDI_IP_Herbalist.

***Available resources to work with***

Resources utilised by the informal providers were either found growing with their immediate environment, for the herbalists, or drugs bought in bulk from outside the state, as in the case of the patent medicine vendors.

*“I use herbs and leaves. .....I get them from the bushes around me, not from a long distance away.”* - 01_AK_RU_IDI_IP_Bonesetter.

*“I also buy my drugs in bulk from pharmacies outside of the state, so that is why I can sell at cheap rates.”* -24_AK_RU_IDI_IP_PMV.

***Trainings***

The informal providers largely did not have formal trainings available. However, the few times that trainings were organized, they were mainly government-funded and involved staff of the PHCs as trainers.

*“No, I haven't (attended any government-sponsored bone setting training). I don't believe there are any meetings for bonesetters Yes, I will gladly participate (if the government holds these trainings).*

*I am not training anyone because it's a family heirloom, so it's just my children to inherit."*  - 01_AK_RU_IDI_IP_Bonesetter.

*“I began by receiving training from West African KENDRACS. I went to the government to gain more knowledge to supplement what I already knew, such as measuring the medicines that you give; they don't show or tell you which leaves to give, but they do teach you the dosages to use. They also give a brief lecture on the roots that I bring to them to experiment and test these roots and leaves before giving them to people, it was also confirmed to be good.”* - 22_AK_RU_IDI_IP_Herbalist.

**Linkage to the formal health system**

A good linkage exists generally between the informal providers and the formal health system, as the providers expressed no hesitation/reservation with referring appropriate cases requiring further/expert management to the PHCs.

*“Of course, I refer to hospitals all the time when I encounter problems. No, I don't (refer to co-bonesetters). I only mean hospitals. No, they don't (refer patients to me). In some cases, if a wounded patient comes to me for treatment, I send him to the hospital first to have the wound treated, and then when the wound is healed, I will work on the bone from the injured area”* - 01_AK_RU_IDI_IP_Bonesetter.

*“I think that one of the major works that the community representatives do is to always look out for pregnant women and sick people, in order to refer them to the right places. I personally follow them up for pre-natal care, ante-natal care, and post-natal care. So, I always refer them to either the PHC or to the General Hospital, as the case may be.”* - 13_AN_UB_FGD_CGM (R1).

***Engagement with the formal providers:*** Most informal providers expressed that they were in constant interaction with the formal providers, although in some instances, their interaction had only been limited to mosquito-net sharing.

*“Yes, I do refer some injuries that are beyond my ability to a health center because I believe there are some injuries that I can cure and others that I cannot and must refer to health centers.”*  - 22_AK_RU_IDI_IP_Herbalist.

*“No role at all. I only collected net when they came.”* - 22_AN_UB_IDI_IP.

***Health Intermediaries***

Aloy-Volunteer workers are recruited at the community level to assist health workers in PHCs. Additionally, there are organisations who recruit community members to mobilize health seekers for the health facilities. Community mobilisers and volunteers are not paid like regular formal providers. Sometimes, they are not paid at all and this is a huge setback to healthcare delivery**.** But they benefit from community health programmes as described by one of the participants:

*For example, the mobilizers, they are community members, and always willing to work with us. Like what I use to entice them is that whenever we are doing anything, I used to involve them, like this measles campaign we are doing now, the distribution of the net, mectizan, mebendazole and other programmes, so that the little thing they are paying, they can also benefit from the stipends****.(provide source****)*

Community mobilisers are huge contributors to healthcare. They are well known in the community and they use this to identify and refer women to health facilities and communicate health information to expectant mothers.

Chinelo

*Types and cadres of health workers*

We found that community volunteers play critical roles within the health workforce space. They most times act as intermediaries between the community and the formal health system.

*Mode of recruitment*

Community volunteers are recruited by NGOs and state governments to mobilise community members for health. It is open to those that are interested but they would have to indicate by writing to the leaders or representatives of the group.

*I really can’t say but we all wear white and we hold meetings in Gwagwarwa, police station and ‘no man’s land.’ When anyone is interested to join us the only thing he has to do is write officially to our leader****.( provide source)***

*Engagement with community*

Volunteers are community members that are recruited to contribute to the community health system. They may not have experience in healthcare delivery but they are taught how to mobilise community members for health.

*Yes, there are some people who are in the community, they studied at the health institution. They are the indigenes of this panshekara, but they are not working in the government health facility. They used them for mobilization and sensitization* ***(provide source)***

*Training*

Volunteers receive training from medical personnel like medical doctors. Some of them are graduates or hold certificates from secondary schools.

Okechukwu

In this study, different informal health providers were actively involved in the promotion of community health across various states studied. They include the Community Health Extension Workers (CHEWs), Community-Oriented Resource Persons (CORPs), community volunteers (CVs) and WDCs who assisted in the promotion of health programs through sensitization and mobilization.

Chioma

In the various communities studied, there were community volunteers (CVs) and WDCs who assisted in the promotion of community health system activities through sensitization and mobilization. In Anambra state, the CVs were involved in the sensitization of the people to access family planning services.

In Kano state, the CVs and WDCs were involved in the sensitization of the people in the community in order for them to have access to health care, accept the SMC (Sessional Malaria Chemoprophylaxis) program and even Covid-19 vaccination. The female community volunteers engaged in house-to-house sensitization and mobilization of the people about health issues and programmes. While the male community volunteers visited different constituencies and mosques to sensitize the male members of the community. In Akwa Ibom state, volunteer workers and even casual workers were recruited for community mobilization when there are programmes like immunization.

According to this study, the CVs were mostly selected by the elders in Kano state, while in Akwa Ibom, only literate individuals who may either be students or NGO workers were recruited as CVs during any community health program. Furthermore, in Kano state, the community volunteers and the WDCs are often trained by the government on how to do the health work. While in Akwa Ibom the selected community volunteers are trained, and screened further before their engagement with the communities.

*We have the community volunteers (CVs) or community mobilizers that work with them (CORPs) to sensitize people to access family planning especially the injectable, the ORX pills and the barrier methods (09_AN_RU_IDI_FP).*

Diuto

Types and cadres include volunteers , mobilizers. These group are not health providers bur are very impactful in mobilizing community members to access health services. In Kano, they are the Volunteer Community Mobilsers (VCMs). VCMs are recruited through community leaders, they select eligible persons/candidates from the society and forward their names.The criteria for recruitment is that a person must be literate, above 20 years old, a resident of the area and has good inter-personal relationship with members of the community. They are formally trained.

A typical VCM engagement with the community is thus described, *“...we act like advertisers in the community hoping that whatever intervention/development we bring, the people accept wholeheartedly. In the course of our work, we first observe the surrounding of an individual, their personal hygiene and carefully make remarks politely where necessary, we inform them about the importance of keeping their bodies and surrounding clean... The next thing we observe is whether there is a pregnant woman in the house, if indeed there is, we encourage her to be attending antenatal care, we go to the extent of accompanying her to and fro journey to the hospital so as just to motivate her”.* *29_KN_UB_IDI_IHW*

Prince

To bridge gaps between communities and health facilities, there are local midwives and the Community Health Influencers and Promoters (CHIPs), that are employed to be on the lookout for community health concerns and to encourage community people to utilize the formal health services closest to them. Those that perform intermediary health services are usually indigenes that live within these communities. The health-focused committees that comprise of religious and community leaders, are usually asked to return to their wards and look out for and recommend those that can serve in intermediate positions.

Local midwives serving in intermediate capacities are provided with materials that can be used to offer first-aid services to pregnant women in urgent situations, before they are referred to the health facilities. The local midwives and community mobilizers working under the UNICEF are given monthly stipend. But before they are engaged, it must be confirmed that they are residing within the communities.

Overall, the intermediaries engage with communities in terms of offering emergency health services, scaling up knowledge and utilization of formal health facilities, and dealing with concerns around vaccine hesitancy that could affect immunization outcomes. This is why most of these persons recruited to act in such capacity must be respectable individuals or are backed by the community leadership to be respected. That way, the community people will listen to them.

Mimi

*Types and cadres of health workers:* Health intermediaries are an essential part of health interventions in the communities notably the VCMs (deployed by UNICEF) and CHIPS responsible for mobilization, house to house collection of information and passing on of information. The traditional rulers were also involved, as well as community influencers, recorders and town announcers.

*Mode of recruitment****:*** The health intermediaries were respectable members of the community, recruited mostly on a voluntary basis with stipends or some form of incentives being offered in some cases. They were recruited by traditional rulers, WDC and community heads based on ability to do the job well - patience, understanding, communication skills and basic education were some key requirements. They also had to be indigenes of the community living in the community. The volunteers apply through the primary health care coordinator's office and if they qualify for the position, they would be recruited.

*Available resources to work with*: One of the respondents noted that mobilizing new people to work with on the campaigns was becoming difficult due to lack of renumeration for previous job(s).

*Engagement patterns****:*** The major engagement pattern for the health intermediaries include regular meetings with representatives (WDC) from the community.

*Engagement with community* :The health intermediaries engage with the community through house to house visit to monitor and register births, and assisting pregnant women and nursing mothers to go to the hospital.

*Training*: In one of the communities, UNICEF, WHO and health facilities were all involved in their training.

***Informal providers***

Aloy-There are many informal health providers in the communities and they include traditional doctors such as bonesetters, healers, TBAs. They have little resources to work with. A TBA attested to contributing money to buying equipment to ensure that her patients experience the best care.

Aside from their apprenticeship position, they also get additional health education and how to handle patients better. The state sometimes improves their capacity through trainings. They are trained to provide basic healthcare information and health management to community members. Informal providers like Chemists and bonesetters can be recruited and trained by the PCN. They act as community informants on disease surveillance as described below:

It is through pharmaceutical counsel of Nigeria that’s PCN which you’re introduced to through a form. And after you fill then they will send a message to you that you are invited to the program at so-so venue at so-so time. So that’s how they recruit people.

Chinelo

The informal health workers include traditional health providers and herbalists, commonly regarded as semi-doctors. They provide home-based treatments to community members that are in need of care.

*Mode of recruitment:* They are recruited through the office of the medical director to assist in providing health care services. However, their duty is mostly to create demand in healthcare for the benefit of community members.

*Community-based health groups:* The informal healthcare providers were the PMVs, TBAs, herbalists, semi-doctors (traditional healers), direct treatment observers and uhwruzuma

*Available resources:* Besides the provision of uniforms and identity cards, most informal providers who collaborate with the formal health system do not have many resources to work with. Therefore, those who provide healthcare services continue to do so using local herbs and materials.

As a herbalist, I make different medications from organic sources (herbs and roots) to treat patients and we package them neatly.

*Trainings:* Although trainings are not regular, some reported that they receive training and support from medical personnel. But we also had a report that some herbalists pay as much as N80,000 to be trained.

*Linkage to the formal health system:* Informal providers that have been engaged by multinational agencies help to identify and refer complicated health cases to the formal health system. It was found that those who are linked to the formal health system also provide them with referral cards which are recognized by the health system.

*Engagement with the formal providers:* Informal providers engage regularly with formal providers. However, this is most notable among those that have been properly engaged by multinational agencies and state governments as mobilisers. They work hand in hand with medical doctors, health workers and the officers in charge.

Okechukwu

Traditional Birth Attendants (TBAs), Bone setters were the two care providers in this category.

*Recruitment, Training, engagement (all health workforce)*

Factors that aided the engagement of the workforce generally with the communities were the use of community groups like Imam, the village youth heads (in Kano) and use of community resource persons, community health workers and other volunteer community members (Anambra).

The main mode of recruitment were through volunteering as observed in Kano and Akwa Ibom States while in Anambra, they were already recruited by the government in consultation with the communities.

Their engagement pattern centered on: offering financial assistance to the poor patients, door-to-door sensitization, community project supervision and offering of medical products for free. The pattern of engagement for the workforce with the communities was facilitated through the help/collaboration of community representatives such as TR, President general, youth leaders, market women leaders CHEWs, Traditional leaders, faith-based organizations as observed in Kano and Anambra.

With regards to training of the intermediary health workers, Anambra State was found to be the only state that reported training the workers before they go into the field.

Chioma

In this study, different informal health providers were actively involved in the promotion of community health system activities across the various states studied. In Anambra state, we found the existence of different informal providers such as the Ward Development Committees (WDC), Community-Oriented Resource Persons (CORPs), Community Engagement Focal Persons (CEFPs) and CHIPS. In Kano state, the PMVs were involved in the Tuberculosis anti-process control program and the TBAs were also actively involved in the education of pregnant women as well as maternal and child care. The TBAs were also actively participating in the promotion of health programmes in Akwa Ibom.

The study further revealed different modes of recruitment of the informal health providers for the community health programs across the various states studied. Under Anambra state, informal health providers were mostly selected by the community people, government and NGOs; In Kano state, the PMVs were selected for the TB program based on the training they received from any health institution. They were also selected based on the number of deaths recorded in their settlements.

The study demonstrated that informal health providers have several resources they work with in order to promote community health. In Anambra state for instance, the CHIPS have the Malaria rapid diagnostic tests (RDTs), Artemisinin-based combination therapy (ACT), oral rehydration salts (ORS), Zinc and paracetamol at their disposal. In Kano State, the PMVs were also provided with RDTs. It was found that most of the informal health providers (CHIPS, CEFP, the PMV, and TBAs) in the various communities studied were well trained either by the government or by the NGOs. However, some inherited the skill from their parents. In addition, this finding indicated that some of the informal health providers had linkage with the formal health system in order to promote community health.

Generally, the engagement pattern of the workforce was centered on meeting with community representatives and the PGs, who in turn inform the community members about the health activities; attending community month end meetings, sensitizing the community members in their houses and meetings, and carrying out family planning activities; “*Yes. I said they (CHIPS) have ACTs, RDT, they have ORS, Zinc, they have paracetamol (09_AN_RU_IDI_FP).”*

Enyi

Informal providers include TBAs.Other informal providers include Patent medicne vendors (PMVs), traditional bonesetters. They are recognised in communities and their services are valued. As a respondent reflected, *“.. Some of them are very good but we know some are quacks who cannot handle such cases..... they are somehow of use to us, mostly people who are staying inside the cities, where the hospital is not very close to them. They are to be encouraged and at least taught when and how to refer.” (02_AK_UB_FGD_CGM-R4)*

Informal providers and ad hoc community members are recruited into programmes set by the formal providers in the programme; *“...Yes, there are criteria because, a village woman can’t be a vaccinator, the vaccinator must be a trained nurse or CHEW. The village people will be recruited as town criers, house to house mobilizers or crowd control people. We recruit them according to their abilities” (26_AN_UB_IDI_PM).*

***Training of informal providers:*** Following recruiment into community health activities, the various mix of workforce are usually given appropriate training, and sometimes re-training. In Kano, for instance, *"based on qualification, our leader selectively brings local midwives from the society and then the selected ones undergo training on how to conduct deliveries"(25_KN_UB_IDI_IP).* Also, in Pankeshera Community, they undergo training and retraining for TBAs leading to reduced maternal and infant mortality.

***Linkages between formal and informal health providers:*** There appears to be some of integration of TBAs into the formal health sector in Akwa Ibom and Kano States*; “As you can see we (TBAs) are in the antenatal station so we work with them” (25_KN_UB_IDI_IP [TBA]).* These linkages between the TBAs with the formal sector in Kano state, have been strengthened through periodic training of the TBAs. Speaking during an FGD, a TBA reflects *“...we’re really enjoying the training because the training is encouraging us, and like before most of the women, when they give birth at home they don’t care to go to the hospital but now as a result of our work they do come to the hospital, and all happens as a result of the training, and all these are among our work, and now even giving birth at home is very rare...” (18_KN_RU_FGD_CGW-R6)*

For referral to informal health workers, there are no direct referral though they believe the TBAs are doing well. So they meet with them, refresh their skills, and visit them. Because they believe the patients trust them more than they trust them but in they refer mental cases to informal sector in Kano *"Yes, we do bring patients to the hospital but never to traditional doctors/herbalists. We are even involved in educating people about the dangers attached to taking traditional medicines excessively. The only exception where we refer people to traditional medicines is when the sickness has to do with the brain (mental problem). "(25_KN_UB_IDI_IP)* There undergo periodic trainings.

In Kano state, the VCMs, interface between the formal , informal providers and the community*, “.Yes, we have linkage and from the ward head up to the district head. We the VCM, normally hold a meeting every month with district heads, to discuss about the health-related problems and if there is need for further assistance the ward head will also report to district head.” (18_KN_RU_FGD_CGW-R5)*

They even hold monthly meetings where they discuss health-related problems and also manage patients jointly. The informal health workers even visit the hospital to monitor their *patients "we are going to the hospital to see how the works is going"* a respondent in Panshekera community, Kano said *"We do go to the hospital sometimes to contribute with some information about what we know, we are professional in the field, and we do provide emergency help if it is not beyond our control and if the help is beyond our control, we refer them to the hospital.....Yes, there is, because they even use to talk to us in the hospital because some of the native midwives doesn’t even bother to send the mothers that give birth to the hospital for proper care which sometimes result to the loss of the baby, and also the hospital workers use to be happy when we refer our clients to them, that is why we have a linkage between we and the hospital to avoid problems."( 03_KN_RU_IDI_IP*).

An FGD respondent also added*," Yes, we have linkage and from the ward head up to the district head. We the VCM, normally hold a meeting every month with district heads, to discuss about the health-related problems and if there is need for further assistance the ward head will also report to district head."(18_KN_RU_FGD_CGW-R5)*

Diuto

These include mainly TBAs, PMVs and some traditional bonesetters. A respondent said they are self-funded and do not get help from the government. There are community informant training as well as TBAs training at the health center that is being organized by the government. For referral, there is also linkage from TBAs to PMVs to hospital workers.

*Training:* TBAs, through some NGO led programmes in Akwa Ibom and Kano states have been receiving formal training at health facilities, by attendance at seminars and workshops, *“I go to community informant training as well as TBAs training at the health center that is being organized by the government where expert doctors are brought in to lecture on pregnancy and childbirth and how to go about ensuring the safety of mother and child, including the knowledge about tools to use during this process. I also attend Family Health Initiative (FHI 360) training to broaden my knowledge.” 06_AK_UB_IDI_IP (TBA)*

This has strengthened linkages with the formal sector and encourages referrals by the informal providers to the formal providers

James (by Tochukwu)

Informal health providers were diverse across communities. Some who ran private maternity facilities provide preventive and curative services for minor diseases. She also immunize children and helps pregnant women deliver babies, immunizes children and helps pregnant women deliver babies. included those who claimed to offer medical laboratory services, native/traditional doctors, and spiritualists. In Anambra, a respondent who described himself as a medical lab operator purported to offer laboratory services and medication prescription for a range of ailments including arthritis, stroke, and orthopedic issues. Interestingly another traditional/native healer in Anambra indicated that he offered services malaria, typhoid, sexually transmitted diseases, rheumatism, using both orthodox and traditional medicine. The native healer claims to be introduced to the practice by his father and that there is a linage pattern in propagating the practice. Although he enjoyed patronage and recommendation within the community, he acknowledges that traditional healers are not always valued in their communities. He claims that traditional healers who come into the practice with doubtful backgrounds are often the ones not recognized. In Kano, a traditional dentist described the space he operated and how he offered services in his community. This lab operator both claims orthodox training which could not be verified. Informal providers are also able to recruit ‘trained’ health workers to work with them.

Prince

Looking through the responses across other themes, there is the presence of bone setters, PMVs, traditional birth attendants and other traditional practitioners in the communities. Community health programmes recruit them through community leaders. The informal providers are well known in their communities, and their services have evidence of success. That is why a particular group in Kano is working to achieve linkage between the formal and informal health providers, in the sense that each of the providers could have unique roles to play in treating illnesses. There is the recognition of informal providers by the formal health system, and there are ongoing trainings for them, some of which are sponsored by the WHO. Areas of trainings include disease surveillance, referrals, handling emergencies, among others. Some of the informal health providers mentioned being provided with certain consumables like gloves, test tubes, cleaning agents like detergents and soaps, etc. However, they said they no longer receive such items regularly, as they now try to purchase theirs.

Mimi

*Types and cadres of health workers:* The informal providers include PMVs and TBAs and are seen in majority of the communities. The women leader in one of the communities noted that PMVs are beneficial since they provide first aid but had some reservations towards the TBAs. Although in some communities, TBAs do not attend to pregnant women. While in others, the women prefer going to them leading to both positive and negative outcomes, according to one of the respondents. In another community, TBAs take part in the routine immunization since they cannot take deliveries at home. Another type of informal provider is the traditional healers who use unorthodox medicine to cure diseases but also refer to the hospital if the illness persists.

*Mode of recruitment****:*** Informal providers were mainly born into the job i.e., inherited from their parents/grandparents and a few underwent an informal recruitment process. One of the respondents, a PMV explained that she worked as an apprentice for years where she was taught how to sell and administer drugs, before opening her own personal store.

*Community-based health groups****:*** The traditional healers have community-based health groups like the Islamic chemist and others that they work with.

*Available resources****:*** Traditional healers prepare their medicines using herbs and native practices handed down by their forefathers.

*Trainings:* Some informal providers had the opportunity of attending trainings or receiving guides from their predecessors. The traditional healers were trained by their masters who also provide advice, guidance, medicine formula and business secrets from time to time in their meetings and classes. Sometimes, TBAs are trained by state primary health care agencies and NGOs on hospital practices. The director of health in one of the communities mentioned that if they were not trained, they would keep on spreading infection because they would not be health conscious and you cannot stop them from practicing their job. There are also seminars held for PMVs to educate them about the job.

*Linkage to the formal health system:* In some communities, these informal providers have linkages with the formal health systems through referring their patients to the hospitals for medical attention. Also, linkages exist between the VCMs and the health system through the ward heads and the district heads and the health facilities.

*Engagement with the formal providers:* Informal providers, that is, the traditional healers engage with formal providers by referring patients to them.

| (Societal) Partnerships |
| --- |

Aloy-The existing partnership was between NGOs and providers; NGO, Government and community. Partnership was recorded between health institutions and community representatives who organize health programmes for their people. Community structures that aid partnership in health programmes could be the religious institutions and sociocultural groups like the Umuada. Others include existing means to communicating members using local news carriers like the town criers. They serve as instrument for mobilizing community for action but they are usually directed by community leaders. In addition to community structures, the will of donor agencies on community helps provide the needed support to leaders and mobilisers.

Community also has their resources in terms of informal health providers. It was therefore easy for donor agencies and state governments to see who to mobilize. Moreover, because of trust that community members have for them, recruiting them into health programmes contributes to success of the programmes.

Chinelo

*Existing partnership/ collaborations*

The types of partnerships that exists includes government and NGO sponsored, NGOs working closely with community leaders, partnerships among media houses and community leaders, informal providers working closely with formal providers and private individuals working with medical personnel. There were also existing collaborations were NGOS and health providers, government and community and organisation and organisation.

*Community structures that aid partnerships:* The community structures that aid partnerships rest on the ward development committees, village heads, and religious heads who make themselves available by working with government and multinational agencies for health.

*Factors that constrain partnerships:* Although community structures like the WDCs support collaborations, they are without finances and therefore may not help meet programme objectives. Similarly, the state may work with NGOs, and may even support them by providing manpower, but they don’t contribute money to help achieve their objectives.

Okechukwu

The major type of partnership observed was that between NGOs /Communities and NGOs/Health Providers. The community structures that aid partnership as observed in two states were the imam, youth head and traditional rulers (for Kano) and for Anambra State are: traditional rulers, women/men organization, respected members of the community, religious leaders and age grades.

Chioma

Both the government and the NGOs understand clearly that the importance of community involvement and participation in the promotion of community health can never be overemphasized. In virtually all the health programs documented, there is clear evidence of community involvement in the selection of key players and determination of programme direction. The community members partnered with the government and even NGOs in mobilization, sensitization and the distribution of medical products and vaccine. They were also actively involved in the data collection. In most of the health activities, health workers and volunteers were further trained and utilized. In the community health system, there were also existing partnerships between the NGOs and the health providers, government and NGOs, and government and Health providers.

*We are partnering with Community Power Network to trace Covid-19 and HIV victims in the communities, when we discover them, we will engage philanthropists and well to do members of the communities to assist in their treatment (17_AN_UB_IDI_CSO).*

Enyi

In Anambra state, the government partners with individual philantropists to enrol community members into the state health insurance scheme.In Akwa Ibom state, communities have also partnered with the govt. through donating community land for the building of health centres. They have partnered with development agencies (World Bank) where communities were given reins to drive community development projects funded by the World bank. The communities also partnered with the government and the Red Cross to integrate and re-settle refugees from neighbouring Cameroun into the community and providing free health care for them.

*Facilitators and Constraints to partnerships:* Community members provide accommodation and security, and any other local assistance the partners may require during the programme. Town criers assist to disseminate information, Communal life was also a helpful structure. Also, people living in the community assist to direct them or do one chore or the other when called upon.

Constraints include, commmunities and development partners, not meeting their various commitments to the partnerships, poses constraints. For example, where community memebers are promised Stipends but eventually not paid. Communities who are unable to match the development funding with counterpart project funds (10%) have also constrained these partnerships in Akwa Ibom State. Other constraints are Cultural beliefs; In Anambra state for instance, "*Again is what people say about ntutu (a native malicious charm characterized by the mysterious injection of tiny metal pin like objects into the victim’s body)- all these things contribute to the disruption of health care. Because when someone has serious typhoid and malaria, or serious illness inside the person, the person will focus on going to remove the ntutu. By the time they will come to hospital, things have gotten worst."(10_AN_RU_IDI_IP)*

Diuto

There are existing partnerships and collaborations. Those identified include between government and communities, and provate sector and communities, for a number of health programmes (COVID-19 sensitization, Vaccination; HIV contact tracing).partnerships also exist for drug distribution. Partnership with Carter, German, UNICEF, SACA, FHI, Oando and other NGOs. Community structures that aid partnerships include Constant meeting as well as contact tracing.

James (by Tochukwu)

In Akwa-Ibom, CSOs reported that they work collaboratively with the permanent staff employed in facilities and have collaborations with informal providers. They believe that informal providers are important because the government has failed to provide the necessary resources needed for facilities to function optimally. “*We have collaborations with them. We see that the TBAs have meetings in facilities monthly and we know their capacities are built and they are encouraged to refer their clients to facilities for ANC*." [IDI, CSO, Akwa-Ibom]. They constantly remind informal providers during their meetings and even visit them too, to find cases and refer to them. They also ensure that counsellor-testers are residents in the local governments where they work. "Even right now, we train them to know how to test for HIV, and they find cases and report to us." [IDI, CSO, Akwa-Ibom]

Prince

Partnerships existed in several forms, but all geared toward improving the delivery of formal health services in communities. Government, Organisations, and Providers partner with community structures to improve reception and delivery of health services in the communities. It was widely believed that if the community leadership structure does not buy into health programmes, there is the likelihood of failure. So, organisations have been deliberate in setting up community structures such as the co-groups and the Voluntary Community Mobilizers (VCM) in northern-Nigeria to bring about the necessary community presence needed for the penetration of health programmes and interventions. Providers in health facilities could play supervisory and coordination roles over community representatives of the health programmes. As part of the partnerships, Community Based Organisations (CBOs) such as the youth organisation, etc., were seen playing assistive roles to health facilities, especially in the areas of footing the bills of indigent patients and in sanitation.

Existing structures in the communities that aided the penetration of community health programmes include Faith Based Organisations (FBOs), Ward Development Commission (WDC), Health Facility Committees (HFCs), broader community leadership, and the informal providers. We found cases of health programmes targeting the numbers that turn up for religious gatherings, as well as health programmes announced in churches and mosques. The authority of the religious leaders was seen to be respected, as their pronouncements could either mean the success or failure of health programmes. Informal providers also helped with organising their clientele base, preparing them to be receptive and accept formal health interventions. And the WDCs and HFCs together with the community leadership are usually at the forefront of receiving donors, external providers, monitoring facilities, and seeing to success of health programmes in communities.

Casmir

There were existing partnerships/collaborations in Anambra and Kano states such as partnerships between individuals and informal providers and NGOs and health providers respectively**.** PMVs were trained, supported, and incentivized by KNCB to identify and refer tuberculosis cases to a central facility for treatment in Kano. Also, the TBAs said they receive items such gloves, cleaning agents, calendars, etc., from the local government health authorities and NGOs, as a way to show them some support in attending to cases within their capacities and incentivizing them to make quick referrals. Further, communities were seen to show willingness in accepting health-related progammes and interventions. Some of the communities donated lands to set up health-related structures, they provided security and guidance to those who implement interventions in their communities, and they were always willing to connect the interventive organisations to their leaders. We noticed that communities in highly urbanized regions might be overlooked by organisations, as they might be said to be developed. This is why in some of such communities, especially in Akwa-Ibom, we hardly found ongoing donor-funded community health interventions.

Mimi

The kinds of partnerships that exist in the different communities are between: organization and individual; government and communities.

*“Yes (the Anglican Church supports him and use that platform for him to come and do his work)*” - 06_AN_RU_IDI_CSO_People’s Mandate

*“They (facility based workers and community based workers that are involved in the health activity) are relating well, because if they aren’t relating well, they wouldn’t be working together”* - 24_AN_UB_IDI_IP

***Community structures that aid partnerships:*** The community viewed some informal providers as being helpful and accorded them their full cooperation. The community structures that aid partnership include respect for the VCMs and CO - group members allowing them to do their job efficiently, and wearing color coded uniforms. Also, mutual respect for each other's role between the local government chairman and the PM. In another community, well respected and responsible persons from the community were used as mouthpiece for these health programs.

***Factors that constrain partnerships:*** Funding was identified as a big challenge, owing workers their renumeration was a big constraint against future partnerships.

Ugenyi

***Existing partnerships/collaborations*** identified were mainly limited NGO & providers, and the government & community. Some of the informal providers expressed lack of in any form of partnerships, while some appeared open to the idea.

*“Yes, there are people that we work together with for example like the traditional herbalist, religious leaders, spiritual healers and we also refer a case that is not related to us.”* - 02_KN_RU_IDI_IP.

Identified existing partnership structures include,

1. Organisation and organisation – *“There are organizations like WHO doing something like what we are doing. We have co-group. We have the T base too those ones too have their own wards where they work. Yes, WASH too is under us. A separate department (in our organization). They (nutrition) used to give food for this infant against kwashiorkor.”* - 28_KN_UB_IDI_IHW.

2. Organisation and individual – *“house-house mobilisers they are popularly known as the VCM- that’s voluntary community mobilisers.”* - 28_KN_UB_IDI_IHW.

3. Government and providers – *“Even the T base, we have the T base too those ones too have their own wards where they work. They (T base) mobilize, they are for the state government. They are birth attendants; they are traditional birth attendant you know because here people don’t like going to hospital to give birth. So, these TBAs now have access to hospitals.”* - 28_KN_UB_IDI_IHW.

***Community structures that aid partnerships*:** These partnerships were aided by community structures such as appropriate community entry through the community head/ traditional ruler, and the presence of medical personnel at meetings to give health talks.

*“You know, before these programmes start, they must have met the chief and the chairman of the community, which I believe will organised from the Igwe’s Palace, once these people are aware, nobody disturbs them. We have the men’s group, women’s group, age grade groups, other ones exist on the town level; A*** Development Union, A*** Diaspora Group, A*** Youths, ....and Umuada too.”* - 22_AN_UB_IDI_IP.

***Factors that constrain partnerships:*** One of the major factors that constrain partnerships include inaccessibility of the PHCs by members of the community.

*“The health facilities should be improved to make it easier for clients to visit the health center, and we should also have doctors visit the health centers on a regular basis. There are also challenges such as a lack of electricity and water, which must be addressed.”* - 07_AK_UB_IDI_CL_Women leader.

| Community organisations & Community participation |
| --- |

Aloy -Key actors here include the Federal government, state government, NGOs (WHO, UNICEF, Red cross, SMC, USAID), CHEWS, doctors, nurses, mobilizers for each settlement, community leaders, community organisations, pharmacists, PMVs. The programs are health education and sensitisation to ensure active community participation. The various health programs ensure that the communities are actively involved with the state of their health.

Chinelo

*Key actors/players/groups:* They include women groups, community leaders, ward development committees, health facility committees, youths, religious leaders, private individuals and community volunteers such as co-group and VCM.

*Nature of action/programme:* The community volunteers go from house to house to mobilise community members to seek healthcare in the health centres. The WDCs and HFC work closely with other community leaders to identify health challenges, call for support and ensure that their members benefit from community health programmes.

*Enabling community structures:* The enabling community structures are the recognized and centralized form of authority. Communities that have recognized leaders that are passionate about healthcare easily accept and support health programmes.

*Approaches to community engagement;* The approaches to community engagement could be top-down or bottom-top. For top-down, multinational agencies and governments who intend to pioneer a project usually engage community leaders to help create awareness in the community. Community groups like volunteers usually play critical roles by mobilizing those in need of health services. As for the bottom-top, community leaders reach out to government and stakeholders to drive their health agenda.

*Constraints:* The community-based groups are mostly headed by men. In the southern parts of the country, insecurity continue to pose threat to the activities of community-based groups.

Okechukwu

Findings from the current study showed that the key actors for community participation in Kano were: Imams, traditional rulers, women leaders, youths, patient groups, rich people in the society, town criers, elders, organizational chairmen, philanthropists and farmers. For Anambra they are CHEWS, President Generals of the communities; traditional leaders; faith based organizations; Market Women Association; the youths, Age Grade meetings and other respected members of the community.

Additionally, in the three states studied, the communities were self-organizing when it comes to community health. In Kanu state, the community members participated actively in environmental sanitation and the rich among them contributed money for the supply of free drugs. They were involved in net distribution, focal persons in the society led the distribution, WDC and town announcers coordinated the distribution and ensured that people cooperated. Some politicians among them sponsored free eye surgeries for their community members. Also in Anambra, the community members contributed buckets and handwash during the Covid-19 lockdown era saga while in Akwa Ibom the community members gave the land for the building of hospital quarters.

A major constraint to community participation in CHS activities in Kano was found to be family root negative belief about the service. For example a woman believing that her daughter-in-law will not deliver in a formal health facility because she delivered her the husband in an informal setting. Another constraint across all the state was finance. For instance, in Kano state, the community-based organization mostly lack the adequate fund to carry out health programmes in the community. Also, they were mostly set back by nepotism.

Chioma

The findings of this study show that community health system in Anambra state, Kano state and Akwa Ibom have drastically evolved to the point of community members being fully represented and carried along especially in Anambra and Kano state. There are different community-based groups and organizations that are participating in healthcare delivery. The WDCs are in charge of community health activities; the CORPs are involved in family planning services; the CHIPS provide services such as counselling, health education, antenatal care, immunization, curative and preventive services, and refer complicated cases to the nearest health facility; The CEFPs oversee the activities of the CHIPS; the CVs sensitize the people on family planning. There are also others who are playing significant roles in community health, like the traditional rulers, religious leaders, president generals, community representatives, youth group, women group, National Association of Private proprietors of Schools. However, their major challenge has been insufficient fund to execute planned health activities at the community level. Also, most of the volunteers were working with little or no incentive which mostly discouraged them. Furthermore, some of the community organizations were mostly excluded from the community health activities especially in Akwa Ibom state.

In addition, the study shows that the communities were self-organizing when it comes to community health, for instance in Anambra state, the community members were involved in the payment of some of the hospital bills of their members who were sick and could not afford the bills for their treatment while in Kano state, the people participated actively in environmental sanitation (cleaning of their drainages).

Enyi

Community groups assist during programmes but are neither formal, intermediary or informal providers. They include the village health committess, traditional heads and president general (PG) of the community, town unions, religous unions, market union, youth groups, town criers. These are recruited on an ad hoc basis and on programme requirement. Other groups in Kano state include the Volunteer community mobilisers (VCMs) and CO-groups, who mobilise community members for UNICEF driven community programmes. The VCMs are considered intermediary health workforce, since they are neither formal nor informal providers, but have a crucial role in the communities as mobilisers. Various community groups and organizations across the study states include men, women groups, age grade. Market unions, informants, religous groups, youth groups, town unions, diaspora women group, political and community development groups. In addition there are also ad hoc community members who are mobilised to assist during community health activities in various ways.

***Actions and activities/ impact of community groups :*** These groups are involved in various health activities and other non-health activities in the community, ranging from capaital projects like erection of new health centres, after which they hand over to the government to manage. They organise community empowerment, sensitization, healthn education activities etc. Through regular meetings, convening at community ceremonies like weddings and church/mosque. All groups ideally are expected to participate in decision making for community activities.

And these are very impactful on community members, *“.. just , just like when a religious leader tells his members don’t take this drug that’s going round in the community, I bet you that campaign is a failure because, you will see that no one from the group will touch the drug. But when these people are involved and sensitized, once they go and announce it to their people at church or market level that, there are people that come to give drugs stays around and take your own and the drug is safe. You will see that the programme is on the move.” 26_AN_UB_IDI_PM.*

In Akwa-Ibom, the committee project management committee. In another community in Akwa-Ibom*" There are many groups like Age grades, market unions, the informants, religious groups, women organization, village health workers, youth group, men group and Town unions. These organizations can affect the progress of any health activity in the community."(26_AN_UB_IDI_PM)*

The presence of the WDC is a key enabler to community engagemnet and participation, *“ they are always there,... anytime you call them they render their support” (30_AK_RU_IDI_FP_OIC)*

*Enabling community structures include***:** Community Participation-Involvement of all the community and, *“ We prioritize the voices of the vulnerable among them, like the older adults, women, physically challenged, etc"(11_AK_UB_IDI_PM_CSDP)*

*Constraints* include**:** No incentives, no payment. "*We went to many places over six months of volunteering. When we come back in the evening, we feel pains everywhere. But we get discouraged by the government and how they handled the funds for COVID. Some of us got just jackets and caps. No incentives, even for transport. And so, till today, no payment"(02_AK_UB_FGD_CGM)*

*Approaches to community engagement:* Each community group has a leader who coordinates and moniotrs group engagemnt and activities. Engagement between groups and wider community takes place at village meeting and other gatherings. Some community groups organise seminars and invite health workers to give health talks. These approaches are used when also engageing groups/ stakeholders external to the community. Others include, Organization of men meeting, August meeting by women, Umuada meeting, Age grade, community engagement and sensitization

Diuto

Key actors or players or groups include the ten man committee set up by the Igwe(traditional head)’s cabinet comprising of the youth, age grades Okpuno Progressive Union (OPU), members of Igwe's cabinet, town unions, WDC, , the age grades, the market women association, Men’s group.

Enabling community structures and nature of action: The nature of action or programme include: Immunization eg measles injection, Creating awareness, Community sensitization and mobilization. They create awareness during programmes which motivates community members to access these services. As a respondent reflects, *“Creating awareness in the community is of utmost importance. There are different social platforms from which people can obtain information at the comfort of their homes but having people they know and trust to come to their houses physically to educate/enlighten them about a certain topic/program encourages them to be compliant more than getting information from social media does.” 29_KN_UB_IDI_IHW*

They also provide security for programme staff as required.

Enabling community structures include the Coronavirus, the WDC and FHC participated in enlightening the people on the importance of the vaccine, to come and collect the vaccine against the Coronavirus.

The major *constraint* across various groups, is the lack of remuneration of Stipends after they have engageg in a health programme. Also, cther constraints include: Irregular payment of stipends, lack of community participation, and Only one labour room being available.

James (by Tochukwu)

Communities could tacitly support health workforce. In Akwa-Ibom, the WDC chairman mentions the importance of education in improving hygiene and discusses how the community used to use money from oil palm produce to support their children's education, including those who went into health fields. ‘Educated’ members of the community were said to rally to support the community in times of need, including health needs: “*since they are well educated people among them like the doctors, professors and others, so whenever they notice a problem, they observe and bring solution to the problem*” [IDI, informal provider, Kano]. In PHCs, community members who are qualified health workers volunteer to assist in the PHC: “…*Somebody who is qualified in health issues like a medical doctor and other nurses may volunteer, they come on their own and say that they want to help out. So, we allow them, it’s this way we can be proud of community involvement*.” [IDI, formal provider, Kano] Community structures such as the women development committee in Kano reported that they supervised health centres to ensure adequate service is provided: “

Communities also form groups that help monitor the activities of health workers within the community. In Kano: “…there is also a committee in the society that monitor the work of the medical workers and make sure they are doing the right work." . Communities also mobilize to fix infrastructure in communities. In Kano, communities provided WASH and other infrastructure that facilitates care in health facilities: " …there was even no light at the hospital but the community people contributed lamps. Again, we also tried to repair the taps to provide the water…"[IDI, community leader, Kano]. Still in Kano, Baban Zaure is also a community organization which serves as watch ,and the Youth community organisation equally contributed one hundred naira (#100) each to sponsor orphans in schools in Kano State. Baban Zaure provides some drugs for ANC, and also buys pampers for new born babies in admission as well as give financial gifts to them in the hospital. They move round the facility to ensure everything is working.

Sometimes informal providers can have some acceptance and influence in the community. An informal lab operator in Anambra indicated that he was allowed to give free medical program in the community. Formal health providers in Akwa Ibom trained TBAs monthly on how to detect HIV and TB victims, testing kits and consumables were given to them with the view to report contact traces/cases back to the formal providers. The epileptic presence of government presence in CHS in Akwa Ibom popularized TBAs

Regarding community participation and engagement, women in Akwa-Ibom raised concerns that they are not able do not hold regular gatherings to discuss their well-being or support each other's health. However, community leaders receive NGOs or government representatives and inform the town crier about health programs to ensure that men, women, and children can attend. Women recognize the importance of health centers and prefer them to traditional medicines, but they also mention the challenges of accessing healthcare in the community and the need for community engagement to raise awareness and support health programs.

Tochukwu

Overall, the conversation suggests that the community has a strong sense of responsibility towards their own health and is willing to work together to promote health in the community. A CSO in Anambra described “*We live as a community, so what we do mostly is to enlighten our people that this one is good and this one is not good, when it comes to health*…” [IDI, CSO, Anambra].

In Kano, the theme seem to be dominated by community's efforts to promote health and cleanliness in their area. The community has initiated various activities to create awareness of health issues such as drama performances, where they educate people about malaria symptoms and how to seek proper treatment. They also participate in cleaning up the drainage and removing waste with the help of trained health workers.

Community members describe a positive perception of community ownership and participation in healthcare as a way to improve community health. They explain that this approach helps to manage community health more efficiently and that health representatives are closer to the people in the community and have a better understanding of their problems.

Overall, the transcripts show communities’ commitment to promoting health and cleanliness and their willingness to collaborate with government and non-governmental organizations to achieve their goals. They rely on voluntary contributions and support to carry out these activities and work towards a healthier community. Community members also contribute to community health by giving their maximum cooperation to the health workers, adhering to their advice, taking their children to the hospital when they are sick, encouraging others to do so, ensuring that their clothes, food, and drinking water are clean, and sharing the knowledge to their family and friends.

Some challenges in the community healthcare system, such as women's reluctance to visit the hospital, rude behavior by nurses, and a preference for traditional medication were highlighted. Self medication was also reported to be common: “…our people whenever they fee headache, they pick Paracetamol and swallow without knowing their health implications…” [IDI, CSO, Anambra]. TBA group's activities was reported to have improved healthcare in their community, including increased hospital visits, better vaccination rates, and better nutritional practices.

Key actors within communities vary. In Kano, WDC actors were mentioned to be key links between health facilities and community members. Other actors identified in Kano to directly or indirectly contribute to health systems in communities These groups include: Gauna association, Gaos for the youths; Gauna women association and Gaous for the women, Jagule women organization, Jihar Kano Ina Mafita, TBA, Podium for Health and Community Development, Hisbah and Vigilante groups. They helped in awareness creation about the community health care; support in keeping the environments clean and protect the health facilities with the help of the vigilante group. Traditional rulers intervene when there is a conflict, and they ensure that women come to health facilities. The TBAs are encouraged to bring pregnant women to the hospital, even if they deliver at home. Formal health providers also provide lectures to men to allow their wives to come to the hospital. The referral system is discussed, and it is noted that the community is informed when there is an outbreak, and the hospital workers go to the location.

Prince

Important actors in the organisation of community health include the youth, women, religious groups, religious and community leaders, informal providers, formal providers, WDC and HFC, and in the north, the co-group and VCM. These persons were said to play vital roles in enlightening people about healthcare and referring cases. The youths and women participated in cleaning the surroundings of the health facilities, and they see it as a responsibility. Informal providers were instrumental in providing first line treatment for emergency cases before referring to a health facility, and the formal providers were useful in providing coordination of the different actors toward improving standards of service delivery in health facilities. And in the north, the VCM and co-groups provided the encouragement to patronize formal health facilities, and in times when facilities were congested, they helped to maintain orderliness.

The nature of programmes organised by the communities to promote healthcare included helping indigents with paying medical bills and providing those that are critically ill with solidarity and hope that they will be fine; assisting in sanitation of the surroundings of health facilities and improved awareness creation related to formal health services; in addition to creating awareness, several community groups mobilized the community populace to participate in health interventions, and in times of emergencies at the health facilities like those of security concerns, the community members that oversee the wellbeing of the facilities were called upon. Trust is built in health interventions when the community structures are involved.

In addition to the foregoing, the media and teachers are also important. While the media helps with publicizing health information and interventions, teachers as enlightened and respected persons in the communities were useful actors in terms of health promotion.

Most of the health interventions or programmes we found in the communities were either self-organising or top-bottom. By self-organising, the community people came together to create and carried out initiatives that will benefit them, such as sanitation, improving health infrastructure, and doing minor repairs of items in the facilities. The health workers tried to link up with informal providers in areas of training and strenghtening referrals. On the other hand, top-bottom organisation meant that governments cascaded health interventions and programmes to the communities without necessarily demanding their input or feedback. In communities were the top-bottom approach held sway, the community members hoped for more involvement in future.

Casmir

In Kano and Anambra states, there were visible evidence of women and youth groups involvement in community organization and participation as key actors. Creation of health awareness, free medication for the elders, eye screening and cleaning of the health facilities are some of the nature of action/programmes noticeable in Kano, Anambra and Akwa Ibom states. The enabling community structures were in place in Gwagwarwa community, Nasarawa LGA and in Panshekara community, Kumbotso, LGA of Kano state. There are many approaches to community engagements observed in Kano and Akwa Ibom states. In Kano State, there were more of top-down approach with minor community self-organizing approach while in Akwa Ibom state, it’s more of community self-organizing approach.

We found communities that were self-organising themselves to improve their health. Enlightened community members could write to international agencies for help. For instance, the Red Cross intervention in Akwa-Ibom came about as a result of a community member who is a refugee writing to the United Nations and requesting for health-related instance. Also, in Anambra, we found wealthy individuals and groups establishing community health insurance schemes, which encouraged a lot of persons to patronize formal health services at no cost. A story was narrated about Akwa-Ibom, where in the past they sold proceeds from palm plantations and could address various community needs, including health. Unfortunately, the government took over the palm trees and they became wholly dependent. It shows that when communities are economically viable, they can take up some of their health challenges and effectively address them.

Mimi

***Key actors/players/groups:*** The women leader educates the women group on the importance of visiting the health centers for treatment and regular checkups. There are equally other organizations in the hospital committee, as well religious groups, youths, organization of blacksmith, retired mid-wives, community leaders and even the entire community as well, all participating in the community.

***Nature of action/programme:*** The programs come as health education, vaccinations, and other medical outreaches.

***Enabling community structures:*** Some enabling structures are general monthly meetings and supervision, willingness of the community to receive the health interventions, and gathering to participate in these CHS activities.

***Constraints:*** In one of the communities, the problem of transportation was a huge constraint because the community is of the opinion that it should be provided by the government. In another community, some perceive CHS activities to be a waste of resources and believe the money should be used for other things. Another community had the problem of community members’ indifference, but it is now a thing of the past.

***Approaches to community engagement:*** The identified approaches to community engagement in the communities were community self-organizing, top-down approach and bottom-up approach.

*“… We do these by organizing programs to help our people health-wise. So, we are trying very much to ensure that our people have good health”* - 06_AN_RU_IDI_CSO_People’s Mandate.

*“Programs come and then we take to communities. So, it is mainly from top”* - 12_AK_UB_IDI_PM_Dir_of_Health.

*“If the community members invite me into their meeting, at least I will take health education to them including family planning, nutrition, good hygiene…”* - 18_AN_UB_IDI_FP.

Ugenyi

***Key actors/players/groups*:** The community members are more inclined to be receptive if activity was first introduced to the traditional ruler and his council and it is found to be acceptable by them.

*“You know, before these programmes start, they must have met the chief and the chairman of the community, which I believe will organised from the Igwe’s Palace, once these people are aware, nobody disturbs them. We have the men’s group, women’s group, age grade groups, other ones exist on the town level; A*** Development Union, A*** Diaspora Group, A*** Youths, ....and Umuada too.”* - 22_AN_UB_IDI_IP.

***Nature of action/programme:*** Behavioural Change Communication interventions were carried out at community level geared towards immunization, environmental sanitation, health seeking behaviour and appropriate preventive practices against malaria and STIs.

***Enabling community structures:*** The community members are more inclined to be receptive if activity was first introduced to the traditional ruler and his council and it is found to be acceptable by them.

***Constraints:*** One of the major factors that constrain partnerships include inaccessibility of the PHCs by members of the community.

***Approaches to community engagement:*** The approach to community engagement was found to be mainly the top-down approach.

| Service Delivery |
| --- |

***Formal Service delivery-A***

Aloy -Service delivery pattern involved offering health services in the health facilities and going for outreach programs. They refer mostly to Teaching hospitals. There was also a tentative referral to informal practitioners. The formal health care providers refresh the skills of the informal providers. The major constraints involved going for outreach to get the children that did not come in for immunization. And also still getting parents to immunize their children when a child in the community has abyss from immunization.

Chinelo

*Patterns of delivery :* Service delivery is mostly within the health facilities and through outreach programs and is run by community health extension workers, and nurses, and may be assisted by a medical doctor. They participate in providing healthcare services in PHCs at the community level.

*Referral pattern:* Formal providers in PHCs may refer patients (i.e. patients diagnosed with HIV) to general hospitals so that can have access to better care.

*Formal connections with informal practitioners:* There is evidence that sometimes, formal providers refer patients to informal providers. This is done when providers perceive that informal providers are in a better position to handle it. For example:

*“They do it occasionally, but not always. Some illnesses look like electric ants wrapped from the front of your body to the back and can kill if not treated properly, so I have leaves and herbs that I use to treat such illnesses.”* ***(provide source)***

*Factors that promote service delivery:* The available human resources and the training provided for them were found to promote service delivery in the community health system.

*Factors that constrain service delivery:* One of the major constraints is lack of trained personnels. Also, some communities do not cooperate with formal health providers especially when they engage them in their respective communities. There were examples where sanitary officers received backlash from community members:

*“Yes, then, they will quarrel with any officer of the village council about why you allowed the sanitary officer to enter their compound, and that no environmental officer has to enter into their house****.”(provide source)***

Okechukwu

Patterns of service delivery in Kano centered around: offering Antenatal, immunization, eye clinic, dental clinic services and offering of drugs where patients have to come and receive these services in the hospital. Others include: providing volunteers who help in sweeping and cleaning the hospital, and also assist patients that cannot walk by carrying them on their backs or wheel chairs.

In Anambra, the patterns centered more on going into the communities to sensitize, counsel, acting as pressure groups and advocacy groups.

In Akwa Ibom the pattern centered on offering routine immunization, family planning, antenatal care, delivery, test and treat services, free Malaria treatment all done in the Hospital . Using the Media (radio) to educate and sensitize people, supporting already existing structures at community level (PHCs) to be able to provide most necessary services to the community and the refugees.

Chioma

In the community health system, formal health practitioners promoted health through rendering of services such as counselling, health education, treatment of patients, family planning, immunization, antenatal, maternal and child care. They also conducted outreaches and community programmes like campaign and refer cases they cannot handle to higher facilities. There is evidence of formal connections with informal practitioners in Kano state as the TBAs refer their patients to the hospital for vaccination and further medical checkup which has reduced the deaths of women and new born children in the various communities. There is also evidence of formal connections with the informal practitioners (TBAs) in Akwa Ibom as the TBAs were mostly invited to the health center when there is prolonged labour which mostly occurs when their clients leave them to go to deliver at the health center. Also, the formal health providers often visited the houses of the TBAs, lectured and monitored them to ensure they are promoting community health. Furthermore, the formal health providers also gave out free delivery kits to the TBAs which were donated by the International Family health (FHI). One of the factors that constrain service delivery in Akwa Ibom is lack of supply of ready-to-use therapeutic foods.

*…an instance occurred where a pregnant woman was brought into the health center and she was in labor for two days, then when asked if she visited a TBA, she said yes, then we called the attention of the TBA, when she came, she used a broom stick from the health center on the pregnant woman and the woman gave birth, we then paid her and that was it. But we do always visit most of these TBAs house and lecture them, monitor what they do and also give out free kits if available to them given by FHI although the free kits aren’t given anymore (34_AK_RU_FGD_CGW, R_10).*

Enyi

***Formal service delivery*** is both facility and community based. Community based service delivery include visits to schools, churches, households.

Referrals from formal sector (PHCs) are usually to secondary and tertiary facilties. Formal referrals are not made to the informal sector.

Formal sector service delivery is mainly constrained by financial resources and lack of medical supplies.

Patterns of delivery include Health education and food demonstration.

Referral of formal to informal is unusual except “mental cases.”

Diuto

Patterns of delivery- Services were delivered in the health centres (facility based)and in the community halls as well as fixed outposts in the community when required. There are immunization, vaccines, drugs even nets…. Act like advertisers

Referral patterns- There was referral but to bigger hospitals, not TBAs. However, in Akwa Ibom state, there are cases where TBAs have been invited to facilities to intervene in difficult and prolonged labour, and they usually succeed in delivering the baby.

There was no known formal connections with informal practitioners.

Factors that constrain service delivery were lack of fund and sometimes strict policies by the government and other stakeholders

Prince

Health workers in the communities offered supportive roles to community health programmes. They participated in publicizing the health programmes, conducted screening and/or diagnosis, as well as involved in treatments. Referrals could move upward, downward, or horizontal. Upward referrals involved the primary health facilities referring cases to higher facilities. While horizontally involved primary health facilities referring cases to medical laboratories, and communities referring those who need the attention of a health worker to the facility rather than depending on informal healthcare services or not even using any. The health influencers and mobilizers in the communities could identify diseases, sometimes new ones, and they go ahead to alert the health facility closest to them. Incidentally, there were cases where formal health providers referred bone-related cases to the bone setters in the communities.

In Kano, there are plans to institutionalize the linkages between the formal and informal health providers. One reason for that is to identify cases that can be uniquely handled by the informal and formal providers. It is believed that such linkage will improve efficacy of healthcare in the communities and will prove responsive to the fact that some people still do have preference for informal health providers.

Delivery and reception of formal services in the communities will improve if community members consider the behaviours of health workers as empathetic enough, which is a core reason they could prefer soliciting healthcare from informal providers. Others are improved infrastructure, especially power supply and to increase the quantity and quality of staff at the health facilities, so that they could access quality health services round-the-clock. Responses from community members showed that patronage of informal providers was due to the suboptimal nature of primary healthcare facilities in the communities. Also, community mobilizing groups were encouraged to be proactive by intensifying messages on the usefulness and availability of health services at the facilities. Finally, some were of the view that informal providers tend to have more patronage because they are religious, thus, calling on formal providers to recognise the religious biases of community members as a way to improve patronage of the health services they offer, especially maternal and child health services.

*“No, just this their belief, women are highly religious people, they sing songs and pray, with the psychotherapy of prayers,,,, some don’t even believe you can give birth under the care of the nurse, so some of them visit these TBAs, risking their lives and end up rushing to the nurses when it is too late and some end up dying” [IDI, WDC Chair, Anambra]*

Casmir (formal and informal merged)

Community members are happy with the patterns of service delivery in Kano State. Formal referral patterns were witnessed in Kano State and Anambra State. There is a formal connection with informal practitioners like traditional healers and bonesetters’ referrals to the formal health providers as witnessed in Kano State. In Kano state, manpower shortages constrained service delivery. In Anambra and Kano states, patterns of delivery were well organized. Checking of BP and weight took place in Igboukwu Community, Aguata LGA and collection of free drugs in Gwagwarwa community, Nasarawa LGA, Kano state. There are strong evidences of referrals from informal services delivery to formal health providers such as hospitals etc., and also from formal to informal service providers such as from Orthopaedic hospitals to bone setters, informal lab technician to chemists etc. in Anambra and Kano states. There are formal connections with informal providers as observed in Anambra state and Akwa Ibom State. Lack of government support and subventions constrain service delivery of traditional bone setter in Isuofia Community, Aguata LGA, Anambra state. In Uyo-Itam, Itu LGA, Akwa Ibom state, lack of health personnel and power in the health centre also constrain service delivery.

In Akwa-Ibom where the Red Cross supports the delivery of healthcare to refugees through primary health centres, steps were taken to provide for referrals to secondary and tertiary facilities based on illnesses that cannot be treated at the primary level. Also, the community people believed in a two-way kind of care, i.e., the use of both orthodox and traditional medicines (herbs). Treatment could start from any of the ends. So, when orthodox medicine fails, they resort to traditional medicines, and vice-versa. We found evidence of the efficacy of herbal medicines, and those that administered them were confident in their efficacy as well. This accounts for why there could be suggestions by formal providers to consider the use of herbal treatments, and informal providers could as well advise the use of orthodox medicines. Clients that have been successfully treated by informal providers were helpful in advertorials of the services of the informal providers and referrals – it is the same for those who live or work around the neighbourhood. It means that the informal providers are indeed popular in their communities, and the respect they enjoy encourage them in service delivery. Informal health providers could provide institutionalized care where they provide accommodation and feeding for their clients. The bone setters knew their limits in wound management, and usually referred such cases to the formal providers, while expecting to commence work on the bone after the wound is managed.

Mimi

***Patterns of delivery :*** One of the respondents was impressed with the service delivery in his community and commended the effort of the ES in ensuring that his people provide the best service at all times to the people. Other service delivery patterns can be seen in the form of house to house visits to render these services to the community in their homes. Services include immunization, family planning, treatment of minor illnesses, antenatal, etc. Another community’s respondent praised the formal service delivery in the community because of the numerous amenities/infrastructures being provided for them, making the work seamless.

***Referral patterns:*** One of the formal providers made it clear that she cannot refer patients to the informal providers and she has no idea if the informal providers refer patients to them.

***Formal connections with informal practitioners:*** According to one of the respondents, there was no collaboration between formal and informal providers due to conflict in treatment patterns, only the TBAs may work in the hospitals. In another community, both formal and informal providers worked together to carry out the CHS activities due to shortage of man power.

***Factors that promote/constrain service delivery:*** Complaints about service delivery come up once in a while to be resolved by the community leaders. Other factors include shortage of staff, nonpayment of salaries and allowances.

Ugenyi

***Patterns of delivery:*** Providers are expected to work every working day of the week.

*“They work every day. Every day they have what to do.”* - 28_KN_UB_IDI_IHW.

***Formal connections with informal practitioners:*** There exist some formal connections between the formal and the informal providers.

*“You know because here people don’t like going to hospital to give birth. So, these TBAs now have to take care of them.”* - 28_KN_UB_IDI_IHW

***Factors that promote/constrain service delivery:*** Financial incentives and stipends provided are generally low, providers are driven more by the zeal to offer help/service to humanity.

Promote – *“Most of them have passion for it because the little they get from it is really helping them.”*

Constrain - *“They have a lot of challenges. A lot. Before you get accepted some people scold them when you get to the households some people will not allow them come inside despite, knowing them. (2) Noncompliance, a lot of them. They don’t want they don’t believe in it. They try their best they inform me; I inform the LGA team without the community member we go there we sensitize and sensitize”* - 28_KN_UB_IDI_IHW.

***Informal Service Delivery-B***

Aloy ***-***While there are informal providers that are supported by NGOs to provide health services, some informal providers do not receive the needed support. Those who were provided with medical products reported that the delivery is not consistent and in fact has stopped running. This makes it difficult for them to provide quality healthcare services. Some however chose to support themselves; they form associations and use it to exert control on their members. The associations also influence the activities of non-members, especially those who do not want to conform.

Informal providers also refer patients with complicated cases to the hospital.

*I refer women who come to see me with complications to the health center; I don't treat them. I can't work on complications like if the baby is turned upside down in the mother's womb; I just refer them to the health center…yes but there are some that do not refer or let me say they have stopped, but we have association to control what we do….*

Chinelo

*Patterns of delivery*

They have house-based treatment and facility-based treatment. Informal providers provide services using herbs and other local substances. They make powder and liquid stuff for the treatment of typhoid and malaria. However, there are those who make use of supernatural methods to treat ailments.

*Referral patterns:* The referral pattern is mostly from informal providers to the formal health system. That is, informal providers, refer complicated cases such as injuries arising from accidents to the hospital for treatment. One of the informal providers mentioned that the formal providers refer patients to them too.

*Formal connections with informal practitioners:* Some informal providers are trained by medical practitioners to provide basic services and they are also taught how and when to refer patients to the hospital. Some are provided with cards to refer patients to the hospital.

*Factors that promote service delivery:* The factor that formal providers sometimes send patients to informal providers acts as one of the factors sustaining their practice. Secondly, some informal providers cooperate to the extent of having a union that serves to regulate their practices. In these meetings, it was found that they share ideas about how to promote their services.

*Factors that constrain service delivery:* Although some multinational agencies and the state government have been doing a lot to incorporate informal providers into the formal health system, some have been left out and have therefore not benefitted from state-sponsored training.

Okechukwu

In Kano, the CHEWs were involved in the distribution of drugs while Bone setter in Akwa Ibom delivers services like provision of accommodation, treatment of burns, orthopedic and physiotherapy services. There is a linkage between the formal and informal health providers in the different states studied. The informal providers like the CORPs and the Bone setters refer the cases beyond their capacities to the doctors or nurses at the community facility. Additionally, in Akwa Ibom state, some serious accident victims are further referred to the Bone setter by the medical doctor at the community health center. There is also evidence of formal connections with formal practitioner in Akwa Ibom as the bone setter is often assisted by the nurse who helps to give injections to his patients. The nurse also helps to take the blood sample for some laboratory tests. Additionally, community health center also helps in some medical tests like HIV tests and other sickness.

Giving TBAs stipends when they refer to formal was observed to reinforce more referrals from the informal and reduce more casualties. In the Informal health care provision, referral/collaboration with formal providers helped ensure maximum results. Collaboration among both the formal and informal was reported to be more effective in improving CHS. This collaboration was observed mostly in Anambra and Akwa Ibom.

*Findings from this study shows that the factors constraining CHS delivery were:* in Kano finance; in Anambra; Finance, language barriers in the sense of translation of some data collected in vernacular; lack of human resources for data management, inability of the government to support NGOs, water scarcity, corruption (embezzlement), continuous patronage of quacks. In Akwa Ibom; attachment to prophesies, poor security, water scarcity, self-diagnosis, refusal to test before treatment, inability of government to provide enough accommodation for staff, and lack of security.

Chioma

According to this finding, in community health system, different health services are delivered by the informal health providers (CHIPS, CORPs, PMV, TBAs). These services include: health education, family planning services, immunization, treatments of the sick members, maternal and child care services.

There is a linkage between the formal and informal health providers in the different states studied. The CHIPS and CORPs in Anambra state mostly refer the sick people they could not handle to the formal health providers. In Kano state, the PMV often refer their TB patients to the hospital for subsequent treatment. They also refer their other sick clients whom they could not treat to the facility. The TBAs also refer their patients and their children to the hospital for vaccination and for the checkup. In Akwa Ibom, the respondents reported that there is a strong linkage between the informal health providers (PMVs and the TBAs) and the formal health practitioners such that the hospitals sometimes refer their patients to the TBAs. However, the referrals were mostly coming from the informal to the formal health providers.

With regards to factors that constrain service delivery; the following were identified: Inability for the community members to seek health advice from experts, poor health seeking behavior, quack patronage, tendency to take services from people that are so close and from people they know rather than professionals, inability to go to the hospital on time, poor remuneration for the field workers, lack of funds for nutrition officers, lack of a CMAM center (Community-based Management of Acute Malnutrition) in Akwa Ibom where acute malnutrition can be managed, lack of ready-to-use therapeutic foods, lack of government counterpart funding, the problem of owing workers, patients fighting heads of facilities due to inability to pay for services, limited, expensive price of licensing for PMV, corruption on the part of elites in the society, societal spiritual stereotypes attached to health, political and fetish interferences, lack of community awareness and low number of workforce.

While health workers creating a relationship between the health system and the community members was seen as a major factor that promotes service delivery. Also, youths’ involvement; making some treatment free such as malaria, support from Red Cross, and support from TBAs were also reported to facilitate CHS delivery. Additionally, the PMVs who worked with the TB anti-process control programme were often motivated which facilitated their service delivery in their various communities.

Enyi

Patterns of delivery include Health education, and role playing. informal health workers refer to the formal health workers. They have linkages to the formal sector for referrals and also inited for meetings and trainings, occasionally, especially in Kano and Akwa Ibom states.

Factors that promote/constrain service delivery include Male dominance in Kano State *"the main problem is noncompliance from the men in the community, in many cases the women give their full support to us but their husbands prevent them from attending ante-natal or accepting immunizations for their babies"(25_KN_UB_IDI_IP)*

Diuto

Patterns of delivery- NGOs hire mobilisers, provide vials, nets etc

Referral patterns- There is a good referral system to labs and hospitals

Formal connections with informal practitioners- No associations except meetings

Factors that constrain service delivery include delay in payment of our monthly salary, unskillful manpower, not being valued (underappreciated)

James (by Tochukwu)

Informal providers provided a range of services, from claims of addressing malaria, typhoid, dental problems, and sexually transmitted diseases to esoteric concerns of spiritual problems. One informal provider claimed to offer laboratory services in his community. Although he claimed to have had education, his qualifications were not verifiable. Another birth attendant in Anambra distributed medical supplies “condoms, injectables and orals” from the government.[IDI, informal provider, Anambra]. The community somewhat gains from owning a healthcare facility because it saves them from traveling too far in seeking small medical assistances: “I can say that the community is gaining a lot from owning a healthcare facility because, it saves them from traveling too far in seeking small medical assistances."[IDI, informal provider,, Anambra]. There is a referral system in place between maternity homes and higher hospitals for complicated cases. The primary healthcare refers patients to the maternity homeowner in the absence of the person in charge of delivery.

Some issues were reported to obstruct the health services. In Akwa-Ibom, several issues that a health center was facing, including water issues, electricity issues, and security issues were highlighted by the WDC chair. The lack of electricity is particularly concerning, as it prevents pregnant women from accessing the health center at night.

Tochukwu

In Akwa-Ibom, the village head states that the community practices herbal medicine, and this traditional medicine is an essential part of their health care. He also claims to support the health center 100%, and his contributions include donating the land for the center and disseminating health information.

Prince

Service delivery from informal providers was largely considered as important, especially as they filled the gaps round-the-clock when the formal health facilities were not functioning, and that they provided first-line and emergency treatments to community people. Further, informal providers were hailed for granting credit facilities to clients, paying more attention to care and health recovery over money, and their involvement of divinity in treatment. There were concerns about late referrals and mistakes, which sometimes could be fatal.

Referrals could happen among the informal providers. For instance, bone setters could send clients to PMVs to purchase medicines or to herbalists for some divine interventions. A case was found in Anambra were an informal provider referred a client to a church to be prayed for, with the thought that the client needs divine interventions to take care of the spiritual causes of the illness before the main treatment procedure can be carried out. Some of the informal providers were mindful of safety, and quickly made referrals to the formal health facilities. Examples included first-time pregnant women, pregnant women with excessive bleeding, etc. In bone-related cases, it was reported that downward referral from the formal to informal was obtainable, especially in times when there is the likelihood of a patient being amputated.

An exceptional case was seen in Anambra where an informal lab operator employed nurses to conduct associated health services like checking blood pressure, BMI, and minor treatments. In different scenarios informal providers like bone setters could call on the services of formal providers like nurses and community health extension workers to help with administering injections, arrest bleeding, etc. Also, health facilities could allow TBAs to work with them in the facilities, particularly providing assistive services, and the TBAs could be sent into the communities to attract and route community members in need of health services to the facilities.

The suboptimal conditions of formal health facilities characterized by poor quality staffing, consumables, and equipment lead to more patronage of the informal providers. The attitude of the health workers also drive people to the informal providers who are considered to be empathetic and understand the plights of clients, including granting them credits and focusing more on their recovery even when they do not have the finance to pay for services. Concerns were raised about wrong prescriptions from PMVs, and that community leaders can help to deal with that. The media was said to be hostile to informal providers amidst claims that they are useful. So, in addition to addressing media hostility to informal providers, tangible and intangible incentives for the informal providers was discussed as important in creating an enabling community health system environment.

Mimi

***Patterns of delivery:*** Informal patterns of delivery include treatment of malaria and pile, TBA services, treatment of common illnesses including dog bites using herbs, and provision of drugs to the community by the PMVs.

***Referral patterns:*** Some of the PMVs mentioned that they refer infants and pregnant women to the health centers for proper treatment, and also some chronic cases to the hospital or laboratory for tests.

***Formal connections with informal practitioners:*** The formal connection that exists is in the form of the referrals, and in another community where both formal and informal providers work together due to staff shortage. Health centers can also train informal providers.

***Factors that promote/constrain service delivery:*** Trainings are a way of promoting utilization of health services. A PMV noted that her services were highly appreciated by the community. In another community, the director of health noted that informal providers can spread infections in the course of their job, although she mentioned that they also play a positive role in terms of helping pregnant women. In another community, introduction of new health care systems gave the impression that people no longer patronized traditional healers but in reality people were subscribing to it more than before.

Ugenyi

***Patterns of delivery:*** Informal providers offer services every day of the week.

***Referral patterns:*** There exists a smooth line of referral from informal providers to the PHCs (government-owned health facilities) for cases beyond their scope of expertise. The informal providers also reach out to fellow providers in some cases.

*“If I have any difficult situation, I ask my colleagues, we meet at Oji River on the last Sunday of every month or any other time they call for emergency meeting. I bring up the case there and they will give me solution. In case of emergencies, I refer them to A**** Teaching Hospital.”* - 22_AN_UB_IDI_IP.

***Formal connections with informal practitioners:*** Some informal providers are organised into a registered union of similar professionals, and they have regular meetings and exchange ideas and provide support.

*“Yes, there are people that we work together with for example like the traditional herbalist, religious leaders, spiritual healers and we also refer a case that is not related to us.”* - 02_KN_RU_IDI_IP.

***Factors that promote/constrain service delivery:*** Some of the promotive factors that influence service delivery include being affordable to community members, ability of work without commensurate enumeration and being able to source medicinal products/plants within immediate environment.

| Medical Products |
| --- |

Aloy

Medical products at the community level are domiciled in the health facilities and formal health providers are used as distributors. They work with community leaders and community mobilisers to ensure that they reach those who need them. However, the community do not have direct control over drugs and medical products. These products may include malaria drugs and ITN, provided by donor agencies like WHO, UNICEF, and ASHIA. Other times, they are provided by the state government.

We recorded instances where philanthropists donate medical products to their community members. They locate them within pharmacies and ask them to provide it free to those who could not afford it. For illustrative:

*Yes, there’s so many people who keep on assisting their people to come to pharmacy. We have pharmacy in different facilities. They just come and donate money when they donate that money they will say this money is for those poor people who don’t have money to pay when they come for treatment****….( please provide source****)*

Chinelo

*Sources of medical products :* The medical products for the community health system are sourced from the government, multinational agencies like WHO and private individuals. PHCs also contribute using service charges to procure medical products and other infrastructures that are needed at the centre.

*Types of medical products:* The medical products that are delivered include delivery kits, drugs and ITN. Other items include eyeglasses, sugar monitoring equipment and high blood pressure equipment.

*Storage:* Community storage of medical products is not mentioned so medical products are stored in PHCs.

*Distribution:* We found that community mobilisers and pharmacists were used to distribute medical products like insecticide-treated nets and drugs. Community mobilisers are well known in their communities and usually act as the link between health initiatives and the community.

*Use of medical products at the community level:* Medical products are distributed by agencies for health but are coordinated by the state government. They are used for the treatment of common community diseases like river blindness.

*Constraints:* Medical products that are distributed to the community can have two challenges: they may not be enough or are of substandard quality. Some health centres sell the free medical products to the community members.

Okechukwu

Most of the respondents reported that medical products and vaccines were mostly supplied by the government and the NGOs like the Institute of Human Virology, Nigeria (IHVN), Marie Stopes, United Nations Population Fund, International Development (USAID) and Family Health International (FHI). However, some drugs and medical products were also supplied by the philanthropists and other members of the community who were wealthy enough. Medical products and drugs supplied during the different community health programmes include: prophylaxis, hematinics, mebendazole, TB drug, HIV drugs, condoms, sanitary pads, and mosquito nets and these were observed to be seen in Anambra and Kano. In Kano, the medical products were distributed at the Hospitals while in Anambra they were distributed into the communities. The study further shows that during the community health programmes all the medical products and drugs were freely distributed to the communities even the family planning service was delivered free of charge.

Chioma

This study shows that in order to promote the community health system, medical products, drugs and equipment were majorly provided by the government and the NGOs. The government freely supplied drugs, and medical products (rapid diagnostic tests, mosquito treated nets for the prevention of malaria, and condoms for family planning, face masks, sanitary pads, delivery kits). The NGOs were also involved in the distribution of free drugs, medical product and food supplements in the various communities studied. The community members were not exempted as they fully participated and coordinated the community health activities. For instance, in Anambra, the community organization partnered with the government and supplied medical products like mosquito nets, condoms, sanitary pads, and face masks during the covid-19 pandemic. And in Kano state, the WDCs contributed in building of the facility and purchasing of medical equipment as well as drugs for the community. However, financial challenge remains one of the major constraints as it has delayed the supply of medical products during some of the community health programmes especially in Akwa Ibom state.

*I already talked about a programme we had last year where we identified 8000 malnourished children, and no provision was made even up till this moment. Once, the state government in 2018/2019 bought cereal lac to help malnourished children that were identified. They owed the people that supplied these products for over three (3) years. So, nobody wants to get involved again. As a result, we have not had any supply, so we can't be talking about utilization (15_AK_UB_IDI_PM).*

Enyi

*Sources* of medical products are mainly from government and then donors (NGOs and philanthropists). Faciltiy staff sometimes also procure supplies from the open market.

*Types of medical products* include antimalarials, mosquito nets, HIV kits, maternal delivery kits, condoms, Antihelminthics, different types of Vaccines including COVID-19. These are usually **stored** in health facilities and distributed under supervision of the facility OIC, but occassionally have been stored in the home of community leaders (Kano state). Community members are not usually involved *in storage and managemnt of drugs.*

*Drug management* also involves the drug revolving fund, which enables facilities to re-stock when they begin to run out of free supplies.

*Distribution and uses of medical products at the community level:* The community patronize both PMVs and the hospitals, pharmacists and chemists to get their drugs.

*Sources:* are usually from government, some from state government through ASHIA, and donors (WHO, UNICEF, NGOs,Cartter) and philantropists

*Types of products:* drugs (premetizine, mebendazole), vaccines, mosquito nets, condoms and other family planning supplies, delivery kits, some laboratory equipments, some consumables

Usually *stored* in the health facilities (pharmacy store) but in Kano sometimes, also stored in the community leader's house

*Distribution*- The drugs are usually distributed in each household within the community, at certain times. Distribution is usually supervised by facility managers but also mbilises community members to be part of the distribution. Distribution can be facility based or community based. When it is community based for instance, “*a town announcer move around and inform the people to come to the community head’s house to receive them . We participate during the distribution of these drugs in order to increase compliance because people trust and respect us. There is transparency during such distributions, all eligible residents of the area have equal right to receive them.” 29_KN_UB_IDI_IHW*

Use of medical products at the community level- We participate during the distribution of these drugs in order to increase compliance because people trust and respect us. There is transparency during such distributions, all eligible residents of the area have equal right to receive them

No Constraints were noted.

Diuto

*Sources:* are usually from government, some from state government through ASHIA, and donors (WHO, UNICEF, NGOs,Cartter) and philantropists

*Types of products:* drugs (premetizine, mebendazole), vaccines, mosquito nets, condoms and other family planning supplies, delivery kits, some laboratory equipments, some consumables

Usually *stored* in the health facilities (pharmacy store) but in Kano sometimes, also stored in the community leader's house

*Distribution*- The drugs are usually distributed in each household within the community, at certain times. Distribution is usually supervised by facility managers but also mbilises community members to be part of the distribution. Distribution can be facility based or community based. When it is community based for instance, “*a town announcer move around and inform the people to come to the community head’s house to receive them . We participate during the distribution of these drugs in order to increase compliance because people trust and respect us. There is transparency during such distributions, all eligible residents of the area have equal right to receive them.” 29_KN_UB_IDI_IHW*

Use of medical products at the community level- We participate during the distribution of these drugs in order to increase compliance because people trust and respect us. There is transparency during such distributions, all eligible residents of the area have equal right to receive them

No Constraints were noted.

James (by Tochukwu)

Informal providers source their health products from the bushes and natural surroundings. A traditional dentist in Kano indicated that "We get it[medications] from the bush, some close to river... while we buy some because of the scarcity since the bush is almost occupied now and sometimes, we order it from neighboring state."[IDI, informal provider, Kano]. In Anambra, an informal provide also reported procuring medical products from the “open drug market in Onitsha” [IDI, Informal provider, Anambra].

There were suggestion that self-medication was common in communities, leading to misuse of drugs which could adversely impact health: "In this community when someone is sick, they like to take self-medication on their own... They don’t even know if it is typhoid, malaria or fever."[IDI, informal provider, Anambra]

An informal provider reported receiving medical supplies and consumables from the government an distributed them in the community.

Tochukwu

The government; WHO, NGOs and philanthropists or wealthy members of the communities in Kano State. Anambra and Akwa Ibom had philanthropists who helped in paying for medical services and took care of eyes problems. Malaria drugs, deworming drugs, mosquito nets, vaccines for polio, meningitis, measles, yellow fever, and tuberculosis. In Kano, drugs were reported to be stored in hospitals. Though, there was no record of storage in Anambra and Akwa Ibom.

Communities may generate funds to purchase drugs for outreach programs. The community generates funds through philanthropy, and community members purchase drugs from the hospital pharmacy. The community has a member attached to the pharmacy to monitor the distribution of drugs to ensure that the drugs are used appropriately. In Kano, the WDC assists the health facility with drug shortage and advocates for the community to support the facility by contributing to its development.

Prince

In some cases, malaria and deworming drugs were donated to governments by international bodies, and the governments liaise with the primary health systems and community leaders to ensure distribution across households. For the informal providers, they buy their drugs from the open drugs market. And in Akwa-Ibom where the Red Cross is supporting a particular facility to provide health services to displaced people resident in surrounding communities, drugs are bought through a Drug Revolving Fund (DRF) package. When drugs are donated to communities, they are stored in the closest primary healthcare facilities. Drug distributors are found among community people. They work together with the health facilities to distribute donated medicines to households in the communities. Distribution is done house to house.

Casmir

In Isuofia community, Aguata LGA, Anambra state, the sources of medical products of the bone setter are from the pharmacy shop. In Gwagwarwa community, Nasarawa LGA, Kano state, the sources of drugs are from the Local Government Area and also from philanthropists. Free malaria drugs and RUTF were observed as type of medical products distributed in Gwagwarwa community, Nasarawa LGA, Kano state. In Gwagwarwa community, Nasarawa LGA, Kano state, the drugs are stored in the hospital. Some organizations like Christian Relief Services distribute drugs in Panshekara community, Kumbotso LGA, Kano State; while drugs are distributed every three months in Gwagwarwa community, Nasarawa LGA, Kano state. Also the WDC and OIC distribute the drugs in Gwagwarwa community, Nasarawa LGA, Kano state. In Isuofia community, Aguata LGA, there is recommendation of the use of English drugs and herbs by the bone setter to the patients. In Uyo-Itam, Itu LGA, Akwa Ibom State, there is challenge of the indigenes not accessing free medicals as the refuges enjoy from the United Nations.

It is important to note that in community health, traditional medicines are considered as medical products. In fact, some community people feel the traditional medicines that are usually made from herbs are more effective than orthodox. The herbs are sources from bushes within the communities and may or may not be sold. While some of the informal providers feel that both products (traditional and orthodox) can be taken together, others feel otherwise.

Mimi

***Sources of medical products:*** Medical products were obtained from the traditional healers, PMVs, state government through the pharmacies, politicians, NGOs (UNICEF, WHO, FHI, etc), LGAs, philanthropists and individuals in diaspora, as well as ministry of health.

***Types of medical products:*** Family planning materials and misoprostol, drugs for pregnant women and infants, hand sanitizers, gloves, and other basic supplies for pregnant women. Anti-inflammatory drugs, DTG drugs, condoms, vitamin A for eye, and polio drugs for children were some of the medical products provided.

***Storage:*** Drugs were kept in the pharmacy with appropriate record keeping in one community, while the health committee was responsible for keeping drugs in another community. After sharing the free drugs, the remaining ones were kept in the facility for proper accountability. The community had no power to receive or store medical products.

***Distribution:*** The community was equally not involved in drug distribution but they were distributed by the VCMs in case of ANC medications after their lectures. In another community, they mobilize people that would share the drugs and WDC, In Charge and OIC were responsible for distributing these drugs. DTG drugs were also distributed to children who were underweight as part of a study, in one of the communities. The health supervisor distributes the drugs to all other wards in the LGA. She also monitors the distribution in her own community by delegating people to that effect. In majority of the communities, health facilities were responsible for distributing drugs.

Ugenyi

***Sources of medical products:*** Medical products are mainly sourced from the provider's immediate environment.

*“I use herbs and leaves. .....I get them from the bushes around me, not from a long distance away.”* - 01_AK_RU_IDI_IP_Bonesetter.

***Types of medical products*:** They include roots and leaves from plants (herbs), fat from certain animals and contemporary health products such as mosquito nets, medicines, and condoms.

***Storage*:** Medical products acquired in bulk are stored with the PHC facility.

***Distribution:*** Distribution is planned to reach every member of the community. Sometimes the necessary/required drugs are not available at the community level.

**Constraints:** Sometimes the necessary/required drugs are not available at the community level.

| Leadership and Governance |
| --- |

Aloy -In some cases, management and payment of health providers are done by the government and the NGOs. The OIC, the Igwes and the youth leader were the prominent leaders in various communities. In other cases, the use of register was used by the health provider to update the LG M&E officer. The community voices were not strong enough to move the village heads because the village heads are keen on letting the NGOs take care of everything. Supervision starts from the WDC Chairman to the OIC to the Community leader to the PG and finally, to the Igwe.

One of the formal providers described the WDC as a key part of their community engagement strategy. Beside building health programmes around them, they also rely on them to mobilise other stakeholders:

*I am the officer in charge of the facility, but when it comes to the meeting, chairman is the chairmen, I am the secretary, but when it comes to affairs concerning the facility, I am in charge. We get to the community through them. For example, if I want to meet the Igwe, I normally go through the WDC chairman, I can’t just go like that because whatever I am planning to discuss with him, it has to do with WDC…(please provide source)*

Community leaders for health report that there is hardly any motivation or incentives for their contributions to healthcare. Most of the time, they rely on advocacy to government officials and then philanthropists and other well to do persons to assist in delivering health projects. This can be discouraging when they do not receive the needed help. As a result, some of the members intentionally withdraw their services.

Chinelo

*Community leadership & roles:* Community leadership roles are enormous; summarily, it is about interfacing with state officials and agencies on community health. The program officers are introduced to the village heads by the community leaders (i.e. WDCs). Then the village heads assist in finding locations or halls for the program and coordinate with town criers, who subsequently inform the members of the community. For example:

*First, the community leaders receive them, not the women, and then take them to meet the village head of the community, who will determine where they will stay to hold these programs and also inform the town crier about this program so men, women, and children can attend****.(Source?)***

*Community-based advocacy:* Community advocacy was carried out by community leaders to politicians, local and state governments. In other instances, it could be within the community. The community leaders, village heads, women leaders were the major leadership roles in the community. Without them there would be no head way for the program. When there is an urgent matter that needs to be solved or addressed, community advocacy is within the community. For instance, they write to the government about security and make requests for health infrastructures.

*If I discover that there is something we need that maybe it is difficult to procure, I contribute by going to a politician with a proposal or letter or something to the politician and tell him that the community need him to assist the health immediately and that it should be done immediately. That’s what I myself contribute****.(provide Source?)***

*Community voices:* There are instances where community voices improved health delivery. For example,

*As a team, we went there together with the team of the community group. We went to the chairman and explained our challenges that we have a problem with delivery at night, so he gave us that generator because then our big generator that is taking the entire hospital is faulty.* ***(provide source)***

*Registration, monitoring, supervision & sanctions:* The health facility committee were said to supervise an aspect of the community health programmes. They work closely with the OICs and state governments to monitor support projects.

*Motivations and incentives:* Community leadership structures’ contributions to health are based on passion and the desire to see an improvement in community health. It was learnt that most of them are not paid but participate voluntarily. However, the security situations in some communities are demotivating the leaders from being committed to health issues. For example:

*Who will gather people, is it the PG? he still wants to live or his committee, they still want to live or the Oji and his cabinet, they still want to live, especially those living outside the community. Anyone who hears what is happening will run away, no body want to buy death, so that is where we are now****.(provide source?)***

Okechukwu

The community leadership structure, which include the traditional rulers, chiefs, president generals, the community representatives, religious leaders, town criers, ward development committees, youth leaders, market women leaders and other community group leaders are active players in health service delivery. They are often consulted before the initiation of any health activity or programme in the community. The community leaders are also involved in the mobilization of their community members, coordination and monitoring of health activities at the community level. There is an existence of community-based advocacy in Anambra state, as the community-based organization advocated for the need for the availability of clean water for consumption in Aguata in 2021, and the Ministry of Water Resources was able to provide it for them. Furthermore, the community health programmes were monitored and supervised by the state and the local government. There are also existing structures (like the WDCs and other team members of the community) who ensured that the community health activities are well coordinated.

Chioma

The community leadership structure, which include the president generals, the community representatives, religious leaders, ward development committees, elders in the community, youth leaders, women group leaders and other community group leaders are active players in health. They are often consulted before the initiation of any health activity or programme in the community. The community leaders are also involved in the mobilization of their community members, coordination and monitoring of health activities at the community level. In Anambra state, the leadership of the community health activities among the stakeholders was a rotational one and the community-based health workers were mostly headed by the president generals. Furthermore, before any health programme or activity was initiated in the community, the community representatives and the president generals must be informed and they in turn were involved in the mobilization and sensitization of their community members. In Kanu state, whenever there is any community health programme, the community leaders train the informal health providers like the PMVs on how to efficiently work with the facilitators of the health programmes, and place them under the direction of the district heads who knows the nook and crannies of the communities.

There is an existence of community-based advocacy across the states studied. The study shows that in some of the communities, the community members advocated that the CHIPS should be well appreciated and given better incentives for the work they do in the communities. The communities also advocated for the expansion of the hospital, the increase in health workforce in the various facilities at the community level, government training of informal health providers and the reduction of licensing fee for the PMVs. Furthermore, there was also an advocacy for the inclusion of the stakeholders at the grassroot level in the planning process in order to promote health service delivery in the various communities.

The finding of this study also shows that for the WDCs to be encouraged to continue to promote community health in Akwa Ibom, they were recognized as frontier of government when it comes to community health activities. The WDC chairman was also made signatory to the account.

Enyi

Community leadership and roles : the wards or villages that make up each community are represented in the leadership structure of most communities, by having a member in the executive committee, *“You know there is no way people from the same ward or village will occupy all the leadership positions in a community. It must be somehow distributed that each village or ward will have their own representative in the project” (14_AN_UB_FGD_CGW-R1).* In some communities, in addition to this community health leadership structure, traditional and religous heads ( ward head, district head, Imams and community elders) are also automatically included in the leadership structure. Where health activities are government funded, leadership and governace structures mainly reside in the formal sector, *"The hospital management board is in charge of the medical help and there is a record book that they take the information of the people that benefit from the help, for verification(03_KN_RU_IDI_IP) "*

Some projects are particular about prioritizing community voices, *“All the community groups are meant to be involved in the community group discussion, the men, the women, the youths, the vulnerable are made to discuss what they need. After that, we embark on voting and after that the need is chosen….. We prioritize the voices of the vulnerable among them, like the older adults, women, physically challenged, etc."11_AK_UB_IDI_PM_CSDP.*

Besides this organic community leadership structure, there are also informal provider associations (PMV Association; TBA association) with clearly outlined leadership structure, membership registration and disciplinary guidelines.

Community based advocacy includes the ward head, district head, Imam and the community elders and there is a clear line of communication with local government authorities, although these are usually constrained by poor or lack of feedback from the government.

Community activities are usually monitored by the government, and in some cases, traditional leaders. Compensation, motivations and incentives are occasionally given.

Diuto

Community leadership and roles- They all have their leaders who do that and report to PG. The head is the OIC…”’ and TBAs. The OIC usually leads health activities but communities are heavily involved . The WDC chairman interfaces between the facilty (OIC) and the community. In some cases, the WDC chairman reports to the community PG who reports to the traditional head, *“I work with the village leaders, and I inform them of what is going on in the facility. I interface between the Village and United Nations for this health center. If I notice any problem, I also involve the Village Head. “25_AK_RU_IDI_CL (HFC Chair)*

Community based advocacy- We organize sensitization for people. We go inside the villages and explain to them what we are doing here. Yes, because most of the time, people neglect health centres and focus their attention on hospitals, but we go and meet them and tell them the importance of the health centre...I mobilise and sensitize the members of the community anytime there is any programme, and they obey me. I also represent them in Akwa where all the community representatives meet with the government, there has be no issue..

Clear line of communication with local government authorities- when there is any health programme, the OIC will call me as the WDC Chairman, I will then meet here to discuss, after that I will call the town crier who will announce to the community members and all of us will community to ensure the programme is done....we write a letter to the primary health care management board; through essential drugs; through PHCC for all the theatre; through zonal director; then to the person at the board headquarters for the program; then to the executive secretary, primary healthcare management board. Later when they give us approval, then we come and start work.....The community will write to the local government, even the state government because it is a community issue, they will let them know that the health centre is built, it is now staffing. Then the Local Government will provide that

Community voices- There are community groups that exist in the community; they include: the youths, the age grades, the market women association, PG, etc.

Registration, monitoring, supervision, and sanctions were by the supervisors and the chief nurse

Motivations and incentives include stipends and in some communities, apparatus were given to them

Conflict Management include resolution of conflicts

James (by Tochukwu)

In Akwa-Ibom, the village head decides where programs will be held, and the town crier informs the community about them. In Kano, separate individuals and groups were identified to monitor health in the community. The ‘*ward health*’ and the ‘*ward development committee*’ were identified to coordinate health activities and programs for the community. They are integrated in the events in PHC and then interphase with the community.

Informal providers tended to have some organization and, in some places, there appears to be governance efforts to organize their activities. In Kano, an informal dentist reported *having “traditional dentist organization Kumbotso, and also state level, although I’m not really committed to the organization." [IDI, informal provider, Kano].*

In Anambra, traditional practitioners were reported to be requested to undergo biometric registration at a specific desk in the local government. This was somewhat acceptable to the traditional medicine practitioners,

*“Yes, it will be good so we will know what individuals are doing. Every time they will be accusing the herbal doctors. If possible, after registering they should call for a meeting so as to know what individuals are doing, and it will be a must that everybody will be present at the meeting” [IDI, informal provider, Anambra].*

Tochukwu

The village head demonstrates leadership by supporting the health center, donating land, disseminating health information, and addressing security issues that affect health: “*I personally, about 19 or 20 years ago, donated that plot (of land) where you see the health centre. So now, you know, if I have donated the land, there's nothing in this world that is bigger than land…*”[IDI, community leader, Akwa-Ibom]. Communities also have many health bodies, and they work together to take the health agenda forward. The community's traditional medicine practices and the health center are supported by the village head and the community. They work together to disseminate health information, address security issues, and support the health center through their contributions.

Prince

Some of the informal providers belong to organisations away from their communities, and they attend meetings there. These organisations serve the purpose of regulating practices to be safe, and a point where ideas and information are exchanged. Community leadership plays pivotal roles in community health systems. They are the gatekeepers, and so, they must be aware of health interventions coming into the communities and are responsible for granting or refusing approvals. The leadership conducts advocacy visits to well off community members to contribute to the health of their communities by investing in the health facilities, and they likewise encourage community members to utilize health services provided for them. They participate in ensuring that facilities are functional, paying attention to raising funds, security, availability of drugs and other consumables, etc. The community leadership draws members from various sectors, paying attention to religious and education sectors, especially in Kano.

Facility heads could solicit help from well off community members to improve the infrastructure and equipment of the facilities. They could carry out such advocacy directly, or work with the community leadership. The community leadership could also solicit help for the facilities by contacting well off community members who can grant funds or provide what the facilities need. Another part of advocacy is encouraging community members to accept health interventions.

In Akwa Ibom, respondents hold the view that the local government authority does not listen to them and they do not reasonably involve in the healthcare of the communities. Requests made to the authorities are ignored. Thus, they have lost confidence in the local government authority paying attention to their health needs, forcing them to rely on donors and community contributions to cater to their health needs, including the health facilities. In other communities, the voices of the people were said to be heard. Instances were given where leaders make requests from wealthy community members and donors, and such requests were granted. Also, some programmes made sure to involve the health workers and informal providers working in the communities as a way to offer them a sense of voice and ownership.

There were programmes where the community leadership together with the health workers working in the community health facilities were tasked to identify those that are eligible for certain health interventions. They go ahead to register those they have identified and see to it that registered persons receive the expected and required health services.

Lastly, while the WDCs and HFCs are interested and generous in making sure that the health facilities in their communities are functional, and that the general community health system is monitored, they wished for some financial support from the government, as a way to encourage them to continue their selfless services. Such support can come in form of imprest or free/subsidized health services in the community facilities. In addition, informal providers are of the view that the media should stop condemning them, as they provide useful health services to members of the communities, especially at crucial times.

Casmir

There is a Co-Group supported by an NGO that is external to the community in Kano State. There are strong community leadership and roles in Kano state which are found in community and religious leaders, chairman and secretary of the communities. There was clear line of communication by the chairperson, health facility committee in Uyo-Itam community, Itu LGA, Akwa Ibom state to the local government authorities, though their requests have not received response. There are strong community voices in Kano state in terms of the WDC recommending volunteers and qualified persons to the health facility. In Anambra state, the herbalists and native doctors are registered. In Kano state, there was evidence of supervision and sanctions on an informal health provider. In Kano state, some members are given incentives in the form of allowances. In Akwa Ibom state, there is evidence of free consumables given to informal health providers. Community leadership supervise health interventions. Informal providers also register as unions, usually for knowledge exchange, government recognition, and to go into defence of their members when necessary.

Mimi

***External to the community:*** Leadership and governance external to the community are the state and federal governments, as well as NGOs and companies. In one of the communities, they were of the opinion that both the government and the citizens are in charge of leadership and governance.

***Community leadership & roles:*** Majority of the communities believe in the community leadership and roles. With the village head being the head of the community. Others believe it to be the native healers and they have a good leadership system with the overall leader from another LGA. Other community leaders include Iman, ward head, district head, WDC chairman, and community elders. Some still believe it is both government and citizens of the community that should lead the community. Other leaders are the formal health care providers.

***Clear line of communication with local government authorities:*** The local government authorities work together with the In-charge of the health facilities.

***Community voices:*** An open line of communication between providers and the community was established to lend an ear to the community voices. Some of the community voices are the health influencers who report on the CHS activities. Also, WDCs are tasked with reporting back to the health facility as well.

***Registration, monitoring, supervision & sanctions:*** A few informal providers belonged to organized labor groups and had their activities moderated by these groups. There is regular monitoring and supervision among the traditional healers with appropriate sanctions for defaulters. Among the formal providers, there is also monitoring and supervision. One the PMVs explained that they have an association which gives her permission to do her business because she is registered with them.

***Motivations and incentives:*** Identified sources of motivation ranged from stipends to having a clear ground to practice their professions by virtue of the fact that they belonged to their professional parent body associations. In one of the communities, VCMs receive salary while TBAs don't. Also, stipends are provided for the volunteers. In another community, the mobilizers that worked previously were not paid and no incentive was given to them, while in yet another community, the government pays the workers incentives.

Ugenyi

Community leadership roles for health providers is domiciled with the respective traditional ruler as with every other member of the community. Leadership of providers external to the community involve supervisors/cluster consultant for the health intermediaries and elected union leaders for informal providers such as the herbalist union.

***External to the community:*** Leadership of providers external to the community involve supervisors/ cluster consultant for the health intermediaries and elected union leaders for informal providers such as the herbalist union.

***Community leadership and roles:*** Community leadership roles for health providers is domiciled with the respective traditional ruler as with every other member of the community.

***Community based advocacy:*** Community based advocacy involves the traditional ruler, his council and the ward development committee.

***Clear line of communication with local government authorities*:** The formal providers and intermediaries for the most part have a clear line of communication with the local government authorities. This was however not found to be the case for the informal providers. The local government authorities were seen as serving rather only a punitive role when things go wrong.

***Registration, Monitoring, Supervision and Sanctions:*** While the formal and intermediary providers were found to have some form of registration, monitoring in place, for some of the sub-groups of informal providers this was absent.

***Motivations and incentives:*** Stipends were given in some instances, although not commensurate with the level of activity carried out.

| Health Financing |
| --- |

Aloy-Health financing is by the state government and NGOs like UNICEF, WHO and UNHCR. The community built one of the health facilities in the communities of the respondents, but the community no longer maintain it. They claim that the government should do the job. The community members in most of the northern states were donating money to PMVs for the treatment of the less privileged ones in society. The philanthropic funding recorded here started from the healthcare providers in the healthcare facilities. They tasked themselves to keep the health facility running. An informal provider attested to helping those pregnant women whether they have money to pay her or not. The rotary club also provided also assisted a health facility to *renovate the health centre.*

Chinelo

*Government/Development partner-funded projects:* Agencies under the state government supply HIV kits to community members. Mostly, they target pregnant women who receive them for free. In kano, it was also found that infrastructures in the health centres like benches and the renovation of facilities are bought from the money obtained from the Kano State Contributory Health Management Agency (KSCHMA).

*Community financing (Levies, donations etc.):* Findings show that community members contribute to financing health projects through levies. Community leadership structures also support projects using their own money. It was also found that communities do come together to support the health of young girls through the distribution of pads.

*Yes. The community came together to form that activity, they are the ones that are contributing that money through different people. So, after they contribute the money they will select the school and community and location like a very poor environment or locality where you can see girls that cannot buy pads to prevent themselves or to create hygiene in their community. Rather than using pads during their period they dress themselves using local methods. They receive pads****.(provide source)***

*Private/philanthropic funding:* We found that many private individuals fund healthcare in their communities as a way of promoting community health. It could be in the form of bringing health workers to conduct tests or procuring drugs for community members. The timing and reach of the programmes are mostly the decision of the private individual.

*Pooling and management--*-----

*Purchasing*

Pharmacists receive finances to buy drugs on behalf of community members and sell them to community members at a subsidized rate. However, vulnerable groups like refugees are permitted to collect it for free.

Okechukwu

Health financing at the community level is majorly done by the government. The government has contributed to health financing through the health insurance programme, basic health care provision fund. However, NGOs like WHO, Red Cross etc. has also contributed to health financing. There is an evidence of community financing as members of the communities in the Anambra and Kano states donated money for drugs and treatment of other poor members of the communities. Some philanthropic individuals in the various communities also assisted in the health financing through their generous contributions.

Chioma

Health financing at the community level is majorly done by the government. The government has contributed to health financing through the health insurance programme, basic health care provision fund, immunization programme, expansion of the hospital, supply and distribution of medical products and drugs. However, NGOs (like WHO, Red Cross, UNICEF, International Family health (FHI) etc.) have also contributed to health financing through the TB anti-process control program, the Sessional Malaria Chemoprophylaxis (SMC) program, distribution of food supplements and medical products. There are also charity organizations and philanthropists who were involved in the organization and funding of community health activities. Furthermore, there is evidence of community health financing as members of the communities in the Anambra and Kano states donated money for medical equipment, drugs and treatment of other poor members of the communities.

Enyi

*Revenue generation*: Various sources of mobilising funds for health activities were identified across the three states-government, international donors, philantropists and user fees. In Anambra state, philantropists enrolled community members into the state health insurance scheme, hence greatly reducing user fees. However, out of pocket payment still constitute a significant part of the funds.

*Pooling and management*: The health insurance funds in Anambra state is pooled in government purse and managed according to stated guidelines. Other funds towards community health are usually in the health facility account and managed by the officer in charge (OIC).However, the health facility committee, usually compsing about 10-15 people ( community members and facility staff), do have to agree on how funds are to be disbursed

*Purchasing:* With the health insurance scheme in Anambra stste, the government purchases services, including provider payment. In some communities, the facility managers (OIC) are in charge of purchasing services?

Diuto

*Revenue generation:* Govt., Development agencies (WHO, UNICEF), Donors, Philantorpists, community contributions. Community financing like levies and donations. Revolving funds in another community. The health projects were sponsored by the WHO, UNICEF, ASHIA and there was a time E. O through the Rotary Club sponsored some projects in the community…contribution…levy…registration.

Private/Philanthropic funding- Donors and sponsors

Pooling and Management: Usually in the bank and manged by the facility or programme team lead. The donors, and team lead of each state are responsible.

*Purchasing:* usually by govt,. facilty managers or the program donors/sponsors. The sponsor or agency is responsible

James (by Tochukwu)

In Akwa-Ibom, A WDC chairman mentions the Basic Health Care Provision Fund, which was allocated to the health center. However, the WDC has not received the fund since the first quarter of the year. The interviewee also mentions an NGO that donated a freezer for drug storage, and in the past, the community used to harvest oil palm to make money to support the health center's needs.

In Kano, community leaders reach out to influential community members to help in mobilizing financial resources to support community health. A leader in Kano stated that: “*We go round the town to those in good government position to request for contribution…we contribute within ourselves… we just select those that can afford to pay…”[IDI, community leader, Kano]*

It was common nonetheless to notice that respondents complained about insufficient support from the government or other community members: *"We don’t have any help coming to the village ooh." [IDI, community leader, Akwa-Ibom].* This lethargy was linked to wider concern for common community good. Informal providers rarely report financing from the government or other sources. One birth attendant narrated that: *"Am not receiving any fund from any person or group of people, I fund the maternity by myself."[IDI, informal provider, Anambra].* However, birth attendants are sometimes engaged by government and civil society funded programs to help in implementing health programs.

Tochukwu

There is no explicit discussion of financing in the transcript, although the village head mentions that the government is not doing what they are supposed to do and that they have been doing their work at their own level. Weak funding however does not limit communities as there were reports of volunteering for projects such as cleaning the drainage system every month to prevent the spread of malaria. In Kano community members are well-informed about the importance of maintaining good health and have taken necessary steps to prevent illnesses. WDC mobilizes the community to contribute to the development of the health facility, and also work with the government for the provision of health services in the community. Community health projects in the Akwa Ibom, Anambra and Kano states were partly sponsored by philanthropists; NGOs, Governments and WHO played very important roles in financing health projects in Kano State, but Anambra and Akwa Ibom lacked the sponsorship of communities’ health projects from NGOs, the Government and WHO.

Philanthropists have been so useful in community health projects in the three states under review. They typically organize and finance community health projects: “*The man is the MD/CEO of* ***XXX pharmaceuticals****. So, I feel that it is because of his pharmaceutical industry that made him to reach out to his people through his field…he is the only person financing it.* “ [IDI, CSO, Anambra].” In Kano State, the different communities have specific account numbers set aside for community health projects. However, this was not seen in Anambra and Akwa Ibom.

Prince

Funding from government for community health was described by the participants as insufficient, and in some places, non-existent. Donors such as WHO, UNICEF, Red Cross, etc., were seen as the popular funders of community health. There is evidence that some health programmes could be sponsored by community members either collectively or individually. These programmes could be preventive, diagnostic, or curative. Informal providers rely on fees for services, which they reported were largely used for consumables to treat the clients. We found no evidence of communities pooling financial resources to cater to the health needs of community members. However, community groups or well off community members could undertake payments for health services used by indigent community members.

Casmir

In Kano and Akwa Ibom states, international organizations such as Red Cross and United Nations fund projects. In Gwagwarwa community, Nasarawa LGA, Kano state, the community contributes through the drugs revolving fund in form of levies. There is philanthropic funding through the Church in Anambra state and through politicians in Kano state. A community-based health insurance scheme is running in Anambra and has helped to improve access and utilization of formal health services. Wealthy members commit to making contributions to the Anambra State Health Insurance Agency (ASHIA), and the agency will in turn channel the funds into a health insurance scheme for community people. This is why in some parts of Anambra, healthcare is free.

Mimi

*Government/Development partner-funded projects:* Majority of the CHS activities were government/development partner funded projects through NGOs and state ministry of health.

*Community financing (Levies, donations etc.):* There is no set amount for the contributions, and such funds were accessible by members when the need arose. Some of the communities finance basic needs through incomes generated from sales of drugs, lab tests, etc. Some have a drug fund where they contribute to buy drugs for the community. Some have monthly levies which they pay for each meeting. In another community, they suggested to the Igwe for the community to raise a purse to support the health workers. While in another community, there was nothing like community contributions, donations or levies.

*Private/philanthropic funding:* Philanthropists funding community activities solo is a very common practice. There are individuals that buy and distribute drugs free to the community through the hospital health committee. Some also print hospital cards anonymously.

*Pooling and management:* The heads of the health facilities (are mostly responsible for collecting/keeping the funds). The communities pool funds to help those in need. The PRO in one of the communities is in charge of the management with accurate account being taken, while in another community, it is the IC and hospital committee that manage the funds with the approval of the community committee. However, some communities have no insurance system or pooling management.

*Purchasing:* One of the respondents mentioned that the person in charge of the health facility handles purchasing.

Ugenyi

*Government/Development partner-funded projects:* Some of the community health activities were said to be funded by the government. However, the intermediaries and informal providers received no government funding.

*Community financing (Levies, donations, etc.):* Informal providers did not have any form of organised external financing.

*Private/Philanthropic funding:* Private/Philanthropic funding is a major source of financing of community-based health activities.

*Pooling and management:* The community has some form of community contributory activity present, and donations could be made to member who require support, with the Igwe's approval. There is also the quarterly allocation from the government for the upkeep of the PHCs.

*Purchasing:* The community has some form of community contributory activity present, and donations could be made to member who require support, with the Igwe's approval. There is also the quarterly allocation from the government for the upkeep of the PHCs.

| HMIS (Information and Learning) |
| --- |

Aloy-Data collected in the various communities included number of pregnant women that come to the health care facility to seek care, number of vaccinated children, details of patients that come to the health facility to get treated, those given nets, those given medications, those given condoms. The details for the number of TB patients and the total number of people treated in the health centres were also collected. There is active participation of community-based groups in drug distribution. Data is transmitted from the health care providers to the supervisors and ten to the consultant. And in some wards, it is transmitted to the M&E officer. TBAs are meant to transmit data to the health centres every day. One of the OICs believe that the data stop transmitting at the state level. The actors involved are consultants, the supervisors, PMV, the OICs and their team, NGOs (malaria consultant, global fund…) and community members.

Chinelo

*What data is collected in the community?:* The data are mostly collected from pregnant women and children between the age of 0-5 years. The information collected includes immunization and antenatal care visits.

*Role of community-based groups :* Community-based groups like town criers or community PROs help in disseminating health information to the public. When policymakers are planning health programmes, they are involved and help to disseminate major decisions to the public.

I’m spreading information anytime government or community organization or NGOs are planning for program, health program in the community we are the first people to know because of the information. We are the ones that pass information to the district head, say advocacy is planning for this, in certain time and place it will happen.

*Data transmission and management (from community to LG and vice versa):* Programme managers who work in community settings usually report their findings to their agencies. We learnt from the study that it could be done using online platforms. However, the role of the community in data management is not immediately clear.

*Actors involved in data mgt. and transmission:* The actors included CHEWs and community mobilisers. CHEWs collect data from health seekers at the facility while community mobilisers collect data at the community level.

Okechukwu

The findings of this study showed that data collection was observed across all the states but was seen more in Akwa Ibom. The community-based groups (like the CHEWs, TBAs) played a significant role in health information management as data was mostly collected during the distribution of drugs and medical products, immunization etc. The Monitoring & Evaluation (M&E) officer of the LGA is responsible for the management of the data and he uploads the data to the DHI platform. Data collected was observed to have a clear line of being transmitted to the higher authorities, starting from the community level to the state level. The data was mostly transmitted to the government for advocacy and follow up. The data was also kept for reference purpose. Data collected were personal information about patients, needs of the community, those who were given tracts and sanitary pads, nature of abandoned projects by government, number of people walking around (for the bone-setter), the leaves used for herbal medication (for the bone-setter), those given drugs, how many people given drugs, and sampled opinions of attitude of workers.

Chioma

The findings of this study show that different data were collected during the community health activities/programmes. Data were mostly collected during immunization, counselling, health education, and sensitization of the community members. There were also data collection during the distribution of medical products (treated mosquito nets, condoms, sanitary pads) and drugs. Data was further collected on the number of exclusive breastfeeding, those that gained weight and those that didn't, and number of children that are set on facilities (under 5).

The community-based groups in Anambra played a significant role in health information management by partnering with the federal ministry of health in the collection of data from communities with TB patients, which was immediately transmitted to Abuja for a follow up. The data collected have always been transmitted to the government for policy development and also kept in the archive for reference purpose. The information collected has been very useful to the community-based organization as it enabled the organization to have an explicit knowledge of what is ongoing in many communities, which they used in communicating to the government for further assistance. The actors involved in data management include all the community health work force involved in the community health programmes.

Enyi

*Data types* collected from different communities includes demography data (phone numbers and addresses for health insurance enrolment), births, immunization records, project -specific data, facility utilization data ( ANC, etc.).

The VCMs (intermediary health workers) in Kano state also help to collect data from the community and transmit to the PHC facilities.

Informal providers (TBAs and PMVs) also keep data and records of their clients. A PMV reflected as follows*, "Yes, I still have my patients' information in the record book, including their phone numbers and addresses. I also have records with the types of illnesses. So, if a patient who is already in my books returns with the same illnesses or sends someone to get drugs for him or her, I'll just go to my books, check the name, and then dispense the drugs based on the illness”* (*09_AK_UB_IDI_IP_PMV)*

*Data management (storage and transmission):* In health facilities, the OIC usually supervises data collection and onward transmission to the LGA and state records department. Other actors besides the OIC are involved as stated by this respondednt, *“ In Anambra.., everything about immunization whether children or adults goes to head of immunization at local government level; Everything about surveillance, early detection goes to the head of disease surveillance Officer; Everything about announcement, how did the work go, what about community people did they come out, are they informed, is done by social mobilization officer (SMO); Everything about nutrition services is taken care of by nutrition officer; and everything about family planning, there is officer that is taken charge of that. And with these, any information you are looking for, you know who to meet about that."(26_AN_UB_IDI_PM)*

Diuto

*Type of data:* All health activities, details of patients and services given to them. Community based data collection is also carried out during outreach programmes or when there is a disease outbreak. *-* “*Two out of the respondents did not keep any records/data but the rest had data….data were collected from the community during the activities. The type of data collected include the number of patients and the details of services rendered to them….we mostly collect information from the caretakers for example when there is outreach, we also collect information from our superiors e.g. when there is disease outbreak we are mobilized immediately to create awareness in the community. We have been trained on how to collect data from the community, the data collected is submitted to the VWS who in turn submit to the LGAs”*

*Role of community-* There are community groups that exist in the community; they include: the youths, the age grades, the market women association, PG, etc.

*Data transmission and management -* Usually transmitted to the local government. ”I know the OIC keeps records of all the activities they have carried out in the community. Data collected are sent to Akwa South Local Government M&E officer monthly ….we mostly collect information from the caretakers for example when there is outreach, we also collect information from our superiors e.g. when there is disease outbreak we are mobilized immediately to create awareness in the community. We have been trained on how to collect data from the community, the data collected is submitted to the VWS who in turn submit to the LGAs”. Actors involved in data management and transmission were the workers in the PHC. No data was found in vital registration in all the communities

Challenge with data collection is that there are no specifically trained data collectors attached to facilities/communities . Also, no specialist in data collection and management in the facility

James (by Tochukwu)

In Akwa-Ibom, programs were reported to be often well-received and do not require feedback. In Kano there were reports of outreach programs that go ito communities to spot and report any observed disease outbreaks or unusual health conditions: “*Through that program this outreach, you are to send your people inside the community, especially something that is related to health matters. When they come if they come across any outbreak or certain condition, they will inform the clinic. From there we know and now go ahead…*”[IDI, formal provider, Kano]

Tochukwu

Detailed reports of HMIS were scarce. However, there were sparse reports of data of birth and death being collected by some program officials. The village head mentions that they disseminate health information to the community through schools and other platforms. The WDC provides information to the community on health-related issues such as COVID-19 and the importance of routine checkups and vaccination. It was also reported in Kano that records of births and deaths are kept in their communities' facilities.

Prince

Generally, the informal providers reported poor collection of data. However, there were exceptions of those that work with organisations that demand specific data like disease history or sociodemographic details. Also, some health interventions in the communities make explicit demand for data pertaining to sociodemographic details, baseline assessment before the intervention, and endline assessment after the intervention. The community mobilizers were also encouraged to be on the lookout for new diseases and report to the health facility closest to them, that will then take it up from there to the appropriate quarters. So, health workers in facilities, informal providers, and community mobilizers in the communities were all involved in looking out for data, reporting and managing them.

Casmir

There were evidences of collection of data such as cholera disease outbreak, number of pregnant women, patients and drugs bought over the counter in Anambra and Kano states. The data transmission is done in Kano through the community members. Actors involved in data management were observed in Kano and Anambra states and they include Individuals, NGOs and government.

Mimi

*What data is collected in the community?:* Generally, record keeping activities were poor, but a few formal and informal providers made the effort to capture details of beneficiaries of their health-related activities. In one of the communities, health facility monthly data records are kept for each department like ANC, OPD, etc. As well as data for cases of malaria in a month, which are sent to the national and international record to keep track of the prevalence. The PMVs are not left out, as they also keep records of patient history. One of the respondents mentioned that she is not aware of how health data is managed even though they might be collected in her community. Another community sends monthly health facility data to the M&E officer at the local government. Another PMV stated that she does not collect data like the hospitals but only sells drugs.

*Data transmission and management (from community to LG and vice versa):* Data collected from the community is sent to the M&E officers at the local government for collation, analysis and dissemination. Data transmission is from the records department to the LGA to the state and then to other organizations. Some communities submit to the M&E officer. While in one of the communities, the WDCs and IC of facilities are part of information distribution. *Actors involved in data mgt. and transmission:* Dearth of competent /trained health personnel, hence no designated data managers. Actors involved include the In charge of every unit, the OPD, and everyone in some cases; with one person making sure the data are up to date.

Ugenyi

*What data is collected in the community?:* Most informal providers did not keep any form of records of kept records just for their personal information/utilization. Whereas the formal and intermediary providers keep records that are submitted monthly to the government and/or respective multi-national organizations, such as records of pregnant women living in the community and malaria rapid diagnostic tests conducted.

*Role of community-based groups:* The community-based groups are involved in deliberations on how to carry out health activities and outreaches.

*Data transmission and management (from community to LG and vice versa):* Data management was either carried out by the individual informal provider or there was a stipulated line of data transmission /management as with the intermediaries working with the multi-national organizations. Specific registers were provided for members of the team working in the community to collect and collate information on all pregnant women living in the community. *Actors involved in data management and transmission:* Data management was either carried out by the individual informal provider or there was a stipulated line of data transmission /management as with the intermediaries working with the multi-national organizations.

***Vital registration (births, deaths, etc):*** Specific registers were provided for members of the team working in the community to collect and collate information on all pregnant women living in the community.

| Multisectoral/Multistakeholder Collaboration |
| --- |

Aloy-Agriculture was mentioned in collaboration with the health centre once. There is no formal security personnel mentioned but the community members through the various community organisation provide security during health programs and to also secure the drugs used for campaigns and outreach. The community members are enlightened about various diseases, how to prevent them and how to treat them. Churches, and religious organisation like the Hisba support health. They support by donating to health projects or by creating ground for health providers to sensitize their members. They also announce health programs and activities in various churches. The umuada also does the same thing. WASH ensures there is portable water for living for drinking. They also curb open defecation and fight against resistance worms and skin diseases. Mechanics ensure the maintenance of dysfunctional instruments during practice. Youths are included as part of the health activities in various communities. They are included as mobilizers, announcers, and recorders while those that have health knowledge are included in the immunization team. They are also involved in sanitation.

Chinelo

*Security-*Community security team help secure medical products and health facilities at night.

*Education-*Schools are used as mobilizing spots for community health programmes. Formal health providers do outreach in schools, especially when the programme concerns young people. They also make use of teachers and principals to reach parents and other members of society.

*Religion & culture-*Formal providers sometimes collaborate with religious groups to help spread awareness about health programmes. The religious leaders make announcements in churches and mosques to worshipers who then comply with the health information. For example:

*We have churches here; we have mosques too. Most of the time, we visited them and told them the important activities we are rendering in the facility. So, they used to tell their people all the services that we are rendering. Fortunately, they come to take these services****.(provide source)***

*Youth & Sports-*Programme officers also collaborate with youth groups during the implementation of community health programmes. Additionally, sports counsels in the community help provide health information to their members. For example:

*I think, there’s sport counsel. Most of the community workers, community citizen too some of them are doing sport. So, that sport also we are getting help from the sport counsel for that health activity in the community. Sport counsel sometimes are sending their representative to come and take the community to see what that community need. More especially for health so they are given that support****.(provide source****)*

*Environment****-***Environment officers usually work alongside community leaders during sanitation programmes. Community leaders help mobilise their members to clean and clear drainages. Market leaders were also said to be involved in environmental programmes.

*Transport*

Some communities have transport associations and they assist by covering transportation costs for patients. During emergencies, they also assign their members to run errands such as transporting patients with emergency cases to secondary care. For example:

*And they are also contributing for the transportation of patients from the local health facility to the hospital. So the transport association, they provide in some of the community like here in Gwagwara. So they assign two to three drivers anytime our community people need that help, they are the ones that will go and take the patient to the hospital for free.(****provide source)***

Okechukwu

Findings from the study showed that the highest level of multisectoral collaboration was in the education sector and was found more in Anambra followed by Kano. The study showed that there is evidence of collaboration between the health sector, ministry of education, and ministry of information in the promotion of community health in Anambra state. In Kano state, the NGOs and government always collaborate with the religious leaders to facilitate community health as the religious leaders are reverenced by the community members in the community, hence they are often used to sensitize the people. Other sectors that were collaborated with to improve health were: agriculture (Anambra),), WASH (Anambra) and environment (Anambra).

Chioma

The study shows that there is evidence of collaborations in Kano state, as the NGOs, and parent-teachers association collaborate to promotes community health. The youths also partnered with the higher institutions to improve health in the community. In Akwa Ibom, the government collaborates with the association of private proprietors of schools to ensure that health related programmes are carried out in their various communities. There is an ongoing community health programme in Akwa Ibom that involves the collaboration of the nutrition officers, environmental workers, and the immunization officers. In Akwa Ibom, the Red Cross health programme led to the collaboration of the health formal providers, the works department and WASH. Additionally, there was also evidence of collaboration between the health workers and the environmental workers in the promotion of community health.

Enyi

*Agriculture and Nutrition*:Farmers association are usually involved. In Akwa-Ibom, there is multisectoral collaboration with nutrition

*Security:* Collaboration with the vigilante group in Akwa-Ibom State

*Education:* Involvement of schools either by educating them in schools or use of the teachers. Some vaccinations are usually carried out in schools

*Religion & culture*: Some vaccinations are also carried out in churches. Religous leaders are also highly involved and commitment to community health matters and support the use of churches as an avenue for appropriate health delivery ( health education, seminars, immunisation). In almost all the communities, religious involvement cannot be over emphasized.

*WASH (Water, Sanitation and Hygiene):* Multisectoral collaboration for water and sanitation in Akwa Ibom state

*Other mutlistakeholders:* These include mainly philantropists and politicians in each community., who embark on various charity health activites.

Diuto

*Education*: Training in the community on girl child education

*Religion and culture*: Religous leaders are very useful and greatly involved in community health activities, mainly through awareness creation, sensitzation and encouraging their congragation (community members) to accept health services/programs. As one religous leader reflects, *“...when an issue arises, the government used to seek our help on the enlightenment of the society because we can pass information more than how ordinary people can pass, because we are religious leaders, because whoever come to your mosque to pray he comes there because he likes you, if not he could have gone somewhere else therefore our words mean a lot to people, and we are contributing 100%, and even in the government if they want to pass information peacefully they seek our help to pass the information to the society. ....... We have been invited sometimes to the hospital for a discussion and we do pass the information from the discussion to the community”. 17_KN_RU_IDI_CL (Religous)*

*Environment:* Opening and cleaning of drainages in Kano state, genrala environmental sanitation, Spraying of insecticides.Visits by environmental staff to educate on hygienne and sanitation (Akwa Ibom)

Improvement of Multisectoral collaborations can be done by recruiting more mobilisers and prompt payment of salaries and training allowance

James (by Tochukwu)

Several stakeholders are involved in the conversation, including community members, health workers, community leaders, NGOs, and government representatives. In Akwa-Ibom, women reported that *“Expert doctors occasionally visit the church to lecture us on health and administer immunizations,” [FGD, women, student]*

*Educations* in schools were also noted to improve the hygiene of children: *"The hygiene of the children improve because they learn health education in the school."[IDI, WDC chair, Akwa-Ibom].*

Collaboration between health workers, traditional healers, community leaders, NGOs, and government representatives can increase access to healthcare, integrate traditional medicine practices into the health system, promote community engagement, and support health programs.

In Akwa-Ibom, a lack of multisectoral collaboration between the government and the WDC, leading to issues such as a lack of electricity and security, which affects the availability of health workers and nurses, making it difficult for pregnant women to access the health center. However, the WDC is responsible for ensuring that the BHCPF are used for their intended purpose, and receipts are obtained to account for what the money was used for. The WDC is also exploring other sources of assistance, such as companies within the community, to push the community forward.

Communities also have local associations that support multisectoral activites. In Kano, a community leader reported *"There is one association called, Gyara kayanka, they sometimes participate… they participate in the education sector, in health and other issues in Kumbotso****."(provide source)***

Communities mobilize for WASH-related activities. In Akwa-Ibom, communities were reported to evolve a sanitation routine: *"We keep on cleaning every Saturday…If you look around, the place is very clean… Any person who does not comply with what is required, we punish them ."[IDI, village head, Akwa-Ibom]*

The *media* was reportedly deployed to raise awareness about health programs in communities. In Kano, media was identified as critical and community leaders and mosques targeted to deliver awareness messages for CH programs: “*Through the media now, through the media as well as through the village heads even at times through the mosques. They pass the information through them*”[IDI, formal provider, Kano]

In Kano, schools were reported to send in students to PHCs to get vaccinated, especially during the COVID-19 pandemic: *“The heads of these schools come here because when the issue of this Corona came then they came here sending their student for them to be vaccinated.” [IDI, formal provider, Kano].* Also in Kano, a government funded scheme was noted to have provided water for the PHC” the water management project: “*That one is provided by the government and it’s dealing through all the 44 local government we have even water related issue. They are now building boreholes, this ROWAS…their main or part of their main function is now building of all these born holes where there is a scarcity of water like in this clinic and of course clinic need water. That’s why they came and built water for us*.” [IDI, formal provider, Kano]

Tochukwu

Several arms of the community contributed to health programs in communities. In Anamba, the corganisation of health programs in and with churches was quite common: “*There are lots of programs organized by Igboukwu people in this town. There are those who organize medical outreach that is done at St. Peter’s Anglican Church and other Anglican Churches that is sponsored by XXXXX every December. Even some Churches equally organize this medical outreach*…*They bring Doctors, Nurses and other professionals for them to come at St. Peter’s Anglican Church…for people will come and explain to them what they are suffering from so that they will be given good healthcare*”[IDI, CSO, Anambra]”

Prince

Connections among the health, nutrition, and agricultural sectors were profound. These sectors come together to pay attention to malnourished children and to teach households about using locally available food materials to prepare balanced diet meals for children. They also work together with community people to conduct surveillance across households in communities, scouting for malnourished children.

*School*s are targets for health interventions and are vehicles through which pupils can be taught about hygiene and good health practices. We found that the health sector partners with the Education sector to gain approval to visit schools to organise health teachings and to carry out administration of health interventions.

*Religious leaders* were found to be very influential in determining the organisation and coordination of healthcare and health interventions in the communities. Donors, individuals, and health authorities partner with them to carry out health interventions in the communities. At times, religious organisations could sponsor health programmes for the communities where they are hosted.

In Kano, the agency that oversees water and sanitation was responsible for ensuring potable water facilities in health facilities. Those heading health units pay visits to those heading the WASH units during community health programmes that will be needing information and supplies *on water, sanitation, and hygiene.*

There are persons employed at the local government level into the department of environmental health. They are referred to as environmentalists and are charged with the responsibility of ensuring environmental hygiene in communities in the local government area. They work under the department of health at the local government.

The finance sector is involved in terms of budgeting and appropriation of finance for community health issues. And health information about diseases and health interventions for community people are circulated through the media. New health related findings from the formal and informal providers are also communicated through the media.

Casmir

There were multi sectoral collaboration in Akwa Ibom and Kano states in agriculture. In terms of security, there is rapid response committee which comprises many sectors in Kano state. There were multi sectoral collaboration in Kano state with respect to Education. In Kano, Anambra and Akwa Ibom states, there are strong collaborations with WASH in the communities. There is strong evidence of the roles of youths in Kano State. In Akwa Ibom and Kano States, the environmental personnel do their works. In Kano state, finance is made available through the WDC.

Mimi

*Agriculture:* The women farmers association (WFARM) is an organization that helps the less privileged and children mostly below 5 years. They distribute fertilizers and also train women in various skills. There was multistakeholder collaboration in one of the communities involving the department of nutrition, agriculture, health, education and the policymakers. They also have the rapid response committee, specifically for surveillance. *“They comprise DSM, the primary health care coordinator, the man from veterinary (that’s from Agric) for practicing, from technical, man from education, information officer and the chairman committee, the DPO from the security site. If something or any disease happens all these people will work together and get it solved”.* ­- 30_KN_UB_IDI_PM

*Security:* The youths provide security during house-to-house immunization process. The rapid response committee was also noted for ensuring security.

*Education:* Ministry of education renovated a school in one of the communities. Health providers also go to various schools to sensitize them on personal hygiene and infections such as HIV and how to avoid them.

*Religion & culture:* Some churches collaborate with philanthropists in the communities to provide outreach programs. Islamic chemists and religious leaders also work with informal providers. *“Yes, there are some people that we work together with like the Islamic chemist, we work with them and some other organizations… There are organizations of the midwives, and religious leaders, they help us with advice even though they are not among the organizations....”* – 05_KN_RU_IDI_IP

*Works, Roads and Housing***:** Construction projects attracted by philanthropists and/or development partners include road construction and repairs, and clearing of bushes, as well as other preventive measures. World bank constructed a road for one of the communities.

*Youth & Sports:* The youths' group was very instrumental to providing security within the community.

Ugenyi

*Education:* Notable philanthropists have carried out activities such as renovation of the community primary school.

*Religion and Culture*: The multi-national organization had one religion focal person present in each LGA where they worked.

*WASH (Water, Sanitation and Hygiene):* WASH activities were improved by the intervention of a philanthropist (EVI) who dug a borehole in the community and enrolled most of the community members under the ASHIA health scheme.

*Youths and Sports*: Private-sponsored youth football tournament by a polio ambassador for R****** Club.

*Transport:* The Catholic Church built a filling station (C****) at N. village and the indigenes are considered first during the recruitment of staff.

*Finance:* The Catholic Church also built a plaza where shops and offices were rented out to indigenes at a very cheap rate.

| Accountability / transparency & corruption issues across the expanded building blocks |
| --- |

Aloy-The healthcare providers are accountable to the community because when they are not doing anything right, the community raises the issue. One of the WDC chairman claimed that why he was made the WDC chairman was because of his transparency and he claimed that he does not get involved in shady businesses. The malaria focal person in communities are responsible for drug distribution in communities so health care providers are accountable to them in that regards. The communities on the other hand are not given money so they do not have anything to be transparent about in that *regard.*

Chinelo

*Leadership/Governance*

Community mobilisers are organized to report to their LGA heads who then report to their cluster LGA heads. The leadership structures are quite organized in ways that checkmate the conduct of the volunteers.

*Health workforce*

Community workforce like informal providers have a union and make rules that govern how their members should conduct themselves. Members who disobey these guidelines are sanctioned based on the stipulated rules.

*Medical products*

Agencies that distribute drugs and other medical items to the community usually have a representative that accounts for them. They record quantities that are distributed and the communities they were distributed to. They also have a way of balancing with pharmacists on drugs that were used for treatments.

Okechukwu

It was observed that there was always a leader who oversaw the activities and they were mostly individuals who do not have to account to any other person. Community participation was also observed (only in Anambra) to be monitored through the help of community representatives. Financial decision/accountability was observed to be unanimous between the project heads and community members; the government and finally private individual. There is evidence of corruption in Anambra state as the money meant for community health programme was not utilized for its purpose as it was embezzled by others including a government official. Transparency in medical products delivery was ensured by distributing the nets almost equally in all households and ensuring that each household does not collect in excess, this was observed in Kano. Adequate service delivery was ensured by conducting periodic service delivery assessment. This was done by accessing the quality of service at satisfaction level of those receiving the services.

Chioma

There are some evidences of accountability/ transparency. With regards to accountability in leadership and governance, it was reported that the community has WDC chairman who is in charge over any health activities that is happening at the community level. Also, there are supervisors sent to the local governments from the national level to monitor CHS delivery activities. With regards to accountability in community participation, it was observed that the community leaders monitor any activity they are involved in within the community. The quality assurance team from the ministry of health regularly checks on the health activities of the WDC across the various communities studied to ensure transparency. In the aspect of finance this was handled by heads of organizations and community representatives. In order to maintain transparency also the WDCs contributes money only when there is a need to purchase a drug or other medical equipment. With regards to accountability in partnership, it was observed that partnerships were documented on paper for easy reference in order to ensure that what the partners claim to do is what they are doing. There was accountability for all the medical products distributed during the community health programmes. All health work force involved in the community health programmes accounted for the drugs they distributed as the products were monitored by the government and donor agencies. With regards to accountability in partnership, it was observed that partnerships were documented on paper for easy reference in order to ensure that what the partners claim to do is what they are doing.

However, there was an existence of corruption across the health sector in the communities studied at Akwa Ibom state as the doctors were barely available at the government hospitals. The fees were also highly expensive too. The informal health providers were not exempted in corrupt practices as they constantly sold expired drugs to their clients as they were not supervised on that. The study also show that health information was often manipulated in Akwa Ibom state.

Enyi

There is transparency in leadership in Awka South, Anambra

Community participation: A particular community in Akwa Ibom state resisted political patronage and rather insisted that government built them a school which was done

Medical Supplies: Efforts are made to ensure transparency during drug distribution

There is transparency in the distribution of drugs in Nasarawa, Kano State

Diuto

In Financing, there are politicians’ interferences

In Medical products, there is a supervisor….There is transparency during such distributions, all eligible residents of the area have equal right to receive them

James (by Tochukwu)

The theme of accountability resonates in some of the transcripts. A chairman of the WDC (Ward Development Committee) in a community health center in Akwa-Ibom identified the importance of accountability and ensuring that the funds allocated for the health center are used for their intended purposes. The WDC is responsible for ensuring that the money is used correctly, and receipts are obtained for all purchases: “*Anything that the money is given for must be used for the correct purpose, and the person is held accountable for the use of the funds, so receipts are obtained to account for what the money was used for. There must be no deviation from what the funds are intended to be used for, and we ensure this by keeping track of all purchases at all times*.” [IDI, WDC chair, Akwa-Ibom]

There were shortcomings with regards to health funds expected at the community level. A WDC in Akwa-Ibom indicated not receiving the Basic Healthcare Provision Fund allocated to the PHC in the community in the first quarter of the year: *“We received it last year and were told that we would receive it quarterly, but we haven't received it since the first quarter.” [IDI, WDC, Akwa-Ibom].* The WDC however indicates his focus to get funds coming to the PHC to be adequately utilized. Another community leader in Kano reported that they monitor health facilities to ensure adequate services: *“and we used to come from time to time to see how patients are being attended.”[IDI, community leader, Kano].*

In Akwa-Ibom, a village head advocated for accountability among health workers: “"*When you give somebody something to do, there should be close monitoring… Any person who does not comply with what is required, we punish them. [IDI, village head, Akwa-Ibom]*

Tochukwu

WDC members were identified to be critical in mediating between governance and community structures. They are accountable to the government, which supports them in the provision of health services. The community is accountable for contributing to the development of the health facility, and the government is accountable for ensuring that the health needs of the community are met.

Prince

The community leadership takes some steps to elicit accountability from health facilities and during health interventions. This, they do, by establishing leadership hierarchy that see to effective utilization of health resources and success of health interventions. Leaders from the state primary healthcare board pay unannounced visits to facilities serving communities, to be sure that service packages are delivered as agreed in policies and designs of programmes.

Through the WDC or HFC, allocated resources to the health facilities are monitored. In certain cases, officials of the WDC or HFC could be co-signatories to the facility's account, just to ensure community's oversight on the utilization of designated facility's finances. One accountability issue pertaining to health workforce is absenteeism. Health workers were reported to be absent at crucial times when they are needed, even in facilities supported by donors.

Some of the WDCs and HFCs in some of the communities were minimally or not involved in monitoring facility's finances. To add, direct payment of volunteers by donors was considered an instance of maintaining accountability. Perhaps, there could be diversion of monies due to the volunteers if passed through the authorities, and such experience could have knock-on effects on their efficacy.

Donors that supply medical products for community health programmes or to facilities make efforts to monitor distribution to end users in the communities. They direct that records be taken and ensure that the records are checked. Members of the WDCs or HFCs are encouraged to supervise and make demands for records on received and dispensed medical products. Collected data before and after an intervention helps with accountability as well.

Casmir

There is strong leadership/governance in Gwagwarwa community, Nasarawa LGA, Kano state through the use of OIC and WDC. In Kano state, there were evidence of community participation in running the affairs of the health centres while in Uyo-Itam community, Itu LGA, Akwa Ibom state, there is poor participation in managing the account of the health centre. Also, there is good account keeping by the pharmacy department while the OIC is not open with the account. It was observed in Uyo-Itam, Itu LGA, Akwa Ibom state of the provision of free drugs by the Red Cross to the refugees while the indigenes buy at subsidized rates. Also, there is evidence of service delivery through the efforts of OIC and the WDC. Where we found DRF operational, we always saw the involvement of community leadership. An aspect of dishonesty was found in the programme for refugees in Akwa-Ibom. A leadership was set up to speak for and cater to them, yet the refugees complain of the inability to access donors and those that in one way want to assist them. In the event of assistances for the refugees, the leadership of displaced persons in Nigeria tend not to be sincere. The leader of the refugees said that they are sometimes threatened with deportation.

Mimi

***Leadership/Governance:*** No conflict issues in leadership and governance were identified. The village head /traditional ruler is always visited first and their acceptability of the activity directly influences that of their subjects.

***Community participation:*** Community participation in health-related activities is affected by the involvement of these groups. In one of the communities, the rapid response committee of the community which had initially not been working was just recently revived.

***Health workforce:*** The health workforce is usually made up of people with at least a little knowledge of health-related events and includes both formal and informal providers in every community.

***Financing:*** Accountability in health financing is a very serious issue in one of the communities such that 15 people make up the committee that must sign off before any withdrawal can be approved, no matter how little.:

***Medical products:*** Issue of using expired drugs for free medical care was discovered by the director of health in one of the communities.

*“There is a free medical care I discovered they are using expired drugs. So, if I did not monitor them to do the right thing, how would I know? I had to stop them somewhere when I discovered some brought expired drugs, so the bureaucracy is not for people not to do what they want to do, it is for the right thing to be done.”* – 12_AK_IDI_PM_Dir_of_Health.

***HMIS:*** Generally, record keeping activities were poor, but a few formal and informal providers made the effort to capture details of beneficiaries of their health-related activities.

***Other sectors:*** Sometimes, communities have to pay advocacy to other sectors so that they can carry out health programs in schools, for instance. Also, community can collaborate with other sectors in carrying out their projects.

| Outputs and benefits of identified activities |
| --- |

Aloy-The outputs recorded were reduced malaria mortality rate, increased use of health care facilities, increased disease awareness and community health needs. Utilisation of services at the health centre is on the increase because of reduced service charges. The use of TBAs was also attributed to their kindness. Major benefits of health activities are better handling of patients as a result of health education and there are happier communities. Improved CHS results in patients seeking treatment again from the same facilities.

Chinelo

*Overall output/outcome of Initiative:* There appears to be an increased desire to seek healthcare services in the formal health system.

*Utilization of different services at community level:* It led to an improved demand for healthcare services, especially maternal and child health services like immunization, antenatal care and child delivery. There were remarks that the initiatives reduce the incidence of polio and other childhood diseases like malaria.

*Benefits:* The initiatives succeeded in improving the quality and quantity of manpower in the communities. Moreover, most initiatives that is community driven receives greater acceptance from the people as noted in this quote:

My opinion is that if the community participated fully in rendering the healthcare services, it will bring more assets to the community living in that particular areas because they are seeing their members participating in their own health programme. It will make them to be achievable. You see most of the people in the community, they want to see somebody that is within them, to health educate them, direct them to come to the facility, to health-related services. So, I think that it will improve the health care, in Kano state as well as in Nigeria in general.

Okechukwu

Findings from this study revealed that CHS activities has led to better/improved facilities/services, increases health indices, corrected health misconceptions, increased willingness to access immunization services, patronize government facilities more instead of quacks, help community members become more aware of health-related issues and their rights, put pressure on the government to implement projects in the communities, helped in the restoration of hope of the traumatized and stigmatized members of the communities, provided mini job opportunities to the community members who are paid stipends, procurement of free nets, free blood transfusions, ensured the availability of safer and better drugs, improve and sustain good health among the community members and enabled more persons in the communities to come out and seek treatment.

Specifically, the introduction of the community health activities or programmes have helped to reduce maternal and child mortality rate, the distribution of the HIV drugs have decreased the number of people dying of HIV/AIDS in Kano state and the provision of the freezer and building for the antenatal and immunization have facilitated service delivery at the community health level.

Chioma

In Anambra state, the introduction of the community health activities or programmes have helped to reduced mortality rates and have helped to increase people's awareness of health-related activities. The health programmes have also helped in the prevention of diseases across the various communities studied. Regarding Kano state, the introduction and distribution of the SMC drugs have drastically reduced the high mortality rate of children below 5 years as a result of malaria. Also, the formal health engagement with the TBAs has reduced the number of deaths maternal and child mortality rate in the various communities studied. Furthermore, The Nutrition programme in Akwa Ibom state resulted to the distribution of vitamin A and deworming of the children. The family planning programme too was extremely beneficial as it helped the women control their birth rate.

Some other benefits accruing from the initiatives are: high level of awareness among community members about community needs especially on health-related issues, increased attending to the needs of the communities by government, change in superstitiously held beliefs by the community members, increased willingness to engage in family planning, decrease in the number of malnourished children.

With regards to ways of improving CHS, it was noted that Multisectoral collaborations can be introduced and strengthened by the government by creating a central desk officer who will keep records of the activities that have been done, the ones ongoing, so that different organizations will not carry out the same project in a community. It was reported that it can also be improved through training of PMV and community leaders; provision of financial support, visitation of schools to give students lectures on how they are supposed to look after their health, proper supervision of PMVs by the government, the government sending out questionnaires which would be returned to the source and used to take decisions, including stakeholders at the grassroots as active actors in planning of CHS and creating a relationship between the health system and the community people.

Enyi

Overall output/outcome of Initiative include:

1. Affordability of health, through health insurances
2. Increase in awareness and sensitization of health in the community,
3. Decrease in illnesses and deaths.
4. Increase in health services and uptake of immunizations.

A number of benefits were observed;

1. The insurance scheme in Anambra state has improved financial access to health services
2. Maternal health sercices (ANC, facility delivery. Family planning) greatly improved, as a result of some of the community health activities in both kano and Akwa Ibom States.
3. It’s also believed that the involvement of people in the heath activities has led to the development of many voluntary groups thus creating more awareness in the society. This has also led to reduces death rate and irregular use of traditional medicines.

Diuto

Overall output/outcome of initiative include improvement in overall health and decrease in death, Increased health awareness and utilization of health services,” The rate at which children died those days has reduced drastically because of the health awareness.” 15_AN_UB_IDI_CL_WDC

*..” the members of the community have to trek a very long distance before they could even get a Panadol, but now, the health facility is at their door post.*“*Again, before now, sensitization on health issue was very low, but after the intervention the community’s awareness on health-related issues was boosted.” 23_AN_UB_IDI_IP (Private sector)*

Utilization of different services at community level- “*Yes, the community members before used to go far from the community in search for medical attention, but now the health centre is here. Also, they provide some services free and others at a very cheap amount. Again, before they bought this health centre here, our people are backward in terms of awareness about their health, but the presence of the community health facility has helped to sensitize the members of community about their health needs and many others”.*

Benefits- One interview said there is no benefit attached. Others said there are Increase in health thus increase in income, increase in awareness….increase in sensitization…increase in awareness…more access to health…,improvement in health...decrease in death... full health package….water, borehole

No risks were noted in the different communities

Improvement of CHS- Decrease in death of children…decrease in distance to health…increase access to healthcare. The PMTCT program has also reduced HIV related deaths

Tochukwu

Overall, communities work to improve health in various ways, but there may be challenges related to financing and perceptions of the value of different types of health interventions. There is also some indication that traditional medicine and modern medicine are both important components of the health system in this community. The WDC assists in the provision of health services in the community by working closely with the health facility staff. They assist with drug shortage and outbreak management, and also educate the community on the importance of coming to the hospital for routine checkups and vaccination.

Data from Anambra State suggests that CHS can be improved via more awareness creation, and that the Community health projects be incorporated into the communities' annual calendar. Kano State suggest willingness among community members towards financial assistance and voluntary services in health; increase in staff employment in the community-based facilities; and Akwa Ibom suggests quality involvement of government presence in CHS. The community health projects in Anambra brought about medical relief to the poor who could not afford hospital bills.

Prince

Informal providers bring health services closer to the communities, although quality is the concern. Cascading health services down to the communities by encouraging community participation through community representatives improved healthcare utilization with effects on reduction in morbidity and mortality in Kano. Informal providers help the indigents with credit facilities and could even provide their services for free to such persons. The informal providers, like the bone setters, provided evidence of having remedies to illnesses that proved difficult to handle by orthodox medicine. However, safety and quality of health practices are the concerns for informal providers.

Casmir

There were good overall output/outcome of initiatives in Anambra and Kano states. There were many benefits to community members in Anambra and Kano states through the various health programmes. There were risks associated with the health activities by community members in Akwa Ibom and Kano states. Furthermore, there is improvement to be made in the health activities carried out in Kano and Akwa Ibom states to solve health challenges in the communities.

Mimi
***Overall output/outcome of Initiative:*** Most of the respondents were satisfied with the overall output/outcome of the initiative and the success they have achieved so far. They believe it has yielded positive results in improving health for the community and providing access to medical care.

***Utilization of different services at community level:*** One of the respondents mentioned that if the community members can access the services rendered in the facility, they will hardly come down with chronic health problems. Having provided affordable health care for all, there is no need for people to risk their lives consulting quacks.

***Benefits:*** Some of the benefits mentioned include free healthcare for those who might not have been able to afford healthcare, and mosquito nets which reduced malaria cases.

Ugenyi

***Overall output/outcome of Initiative:*** Members of the community are able to observe that maternal deaths have reduced due to the effects of some of these activities and also the incidence of children developing poliomyelitis disease.

***Benefits*:** In general, the health states of the community members have improved.

***Risk*s:** Sometimes the necessary/required drugs are not available at the community level. Financial incentives and stipends provided are generally low, providers are driven more by the zeal to render help/service to humanity. There is need to improve the state of PHCs to make them more easily accessible and acceptable to members of the community. Their present poor state in most communities serves a s a constraint to members of the community.

***Improvement of CHS:*** Behavioural Change Communication interventions were carried out at community level geared towards immunization, environmental sanitation, health seeking behaviour and appropriate preventive practices against malaria and STIs.

| Ways to improve CHS |
| --- |

Chinelo

*Improvement of CHS:* Community health initiatives encouraged community members to continue promoting health in their communities.

Okechukwu

Findings from this study revealed that CHS can be improved through the provision of water and by everyone coming together to contribute their little effort. The findings revealed that CHS can indeed be improved by the government introducing and strengthening multisectoral actions this they can do by bringing all the sectors together under one umbrella for collaboration and development of community health. There is also need to appoint special assistant to the governor on Civil Society Organization and as well set up a new management team in the CSO to effectively plan on how to collaborate with other sectors. By ensuring that there is enough accommodation in the hospitals and PHCs for the health workers, they will tend to be more available at work.

Also, as a result of limited human resources in the health sector, there is a problem of data collection, so there an advocacy for the government to employ more people specifically for the purpose of data collection.

James (by Tochukwu)

Akwa Ibom suggests that CHS can be improved if community members take the responsibilities of equipping their health centres rather than waiting for government endlessly.

Prince

To improve community health systems, participants recommended more attention to the involvement of local actors in health programmes and interventions, as well as in the affairs of health facilities. They also recommended the need to intensify intersectoral collaborations so as to benefit from comparative advantages in addressing diverse health concerns of communities. Informal providers advocated for incentives, which could be tangible or intangible, as they believe that they play important roles in community health, especially by providing first line and emergency health services, and in handling some conditions that orthodox medicine find too difficult to address. However, regulating and supervising the informal providers for the purpose of safety is crucial. Overall, improving the functionality of health facilities was profound across the communities. This will lead to improved usage of formal health services, as well as provide a credible referral destination for the informal providers.

Casmir

Further, since community health programmes are usually donor funded, there is need to prepare for the exit of donor funding. For instance, the Red Cross Programme in Akwa Ibom is time-bound and the community people are already experiencing reduction in health service coverage. There is the worry that if the donor exits, the refugees might begin to suffer a lot. Again, the local government leadership, as well state government, must devise means to be responsive to the community people. Negligence and weak feet of the government dampens the morale of the community people to involve in community health. We had evidence of community members showing tiredness in the pursuit of community health needs at the level of government, since no one listens to them by replying their letters or be responsive to their demands.

Mimi

Some of the community members expressed areas of improvement in terms of more support towards health activities and programs by organizations, good roads, water, electricity, fumigation of farms, security. Another area of improvement is awareness creation for the health activities so that more people can participate. And community involvement without religious bias. Also, specific dates and times should be allotted to these outreaches. Another respondent mentioned that the community should demand support from their illustrious sons and daughters at home and in diaspora. Support can also be gotten from politicians, and attract collaboration with external organizations by giving their land for development.

| Other Emerging issues |
| --- |

Okechukwu

The findings from this study also pointed out other emerging issues affecting CHS delivery which centered on the high rate of morbidity especially in malaria and people that rely on informal medicines dying more than those that refer themselves to the hospitals; high rate of corruption and the need to sanitize the system; leadership problems on the part of the government, the general insecurity problems in the country, difficulty ascertaining the appropriate prescription for herbal medication used in informal setting and the government taking primary health centers more serious.

Chioma

With regards to other emerging issues, it was noted that the rights of the community members were abused, their needs were neglected and projects in their communities were abandoned. Some of the communities complained that they have been writing to the government but nothing has been done about their problem and they are tired of writing. Poverty/lack of funds for the community members to buy basic care services, communities feeling they are cheated in the distribution of medical products, issues of accommodation for health care workers, high exorbitant fees in the Health centers, unavailability of doctors, not taking monthly sanitations seriously like before, employment of poorly qualified health workers, giving birth to too many children, superstitious belief that when one helps a child she could be trying to use them for witchcraft, the defaulters who don't follow the cleaning rules bribing inspection teams, problem of involving people from different communities in a particular community rather than using those from the community itself, and the problem of selling drugs to indigenous people while they are given free to the refuges were all noted as emerging issues.

Ugenyi

Appeals are being made to the government to improve the availability and quality of community health services.

| Summary/Conclusions |
| --- |

Chinelo

There is need for all hands to be on deck to ensure that health is promoted in communities. The rich should also help the less privileged. Finally, there is need for collaboration between the formal and informal providers because there are some ailments that the traditional healers can cure but the hospital would not know what to do about them.

Okechukwu

The study shows that the health of community members has improved since the strengthening of the community health system through multisectoral collaboration. The collaborations between the ministry of health, NGOs, community members and other sectors of the government (like ministry of education and ministry of information) have created health awareness, and the need to facilitate, improve and sustain good health among the community members.

Chioma

The study shows that the health of community members has improved since the strengthening of the community health system. The collaboration between the government, NGOs and community members has created health awareness, and the need to facilitate, improve and sustain good health among the community members.

Tochukwu

In summary, transcripts show that the community leaders and healthcare providers work together to ensure the provision of healthcare services. Government programs, projects from multinational agencies and civil society and community members provide funds and resources community health projects. Healthcare providers who may come from the grassroot or from the diaspora or hired by the funders educate the community members about the importance of using formal health services. The traditional rulers and TBAs play important roles in the healthcare system, and the hospital activities are checked to ensure that they follow the guidelines. Formal healthcare workers are expected to respond promptly to outbreaks and disease outbreaks in the community. Overall transcripts suggests that communities has some systems in place to promote good health. However, the community faces challenges related to inadequate staffing at the local health facilities, lack of specific allocations for health, water, and sanitation. There is also a need for closer monitoring of staff to ensure that they are doing their jobs effectively.
